# Diagnostic value of combined serum CEA and CA19-9 in colorectal cancer A meta-analysis

**DOI:** 10.1016/j.isci.2026.115639

**Published:** 2026-05-04

**Authors:** Liushaoqiu Zhou, Songlin An, Yuanzhong Zhou, Yonghong Ma, Guanyu Li, Tao Zhang, Daimin Xiao

**Affiliations:** 1 Department of Laboratory Medicine, Affiliated Hospital of Zunyi Medical University, Zunyi 563003, China; 2 School of Laboratory Medicine, Zunyi Medical University, Zunyi 563000, China; 3 Department of Stomatology, Daping Hospital, Army Medical University (The Third Military Medical University), Chongqing 400042, China; 4 School of Public Health, Zunyi Medical University, Zunyi 563000, China; 5 Key Laboratory of Maternal and Child Health and Exposure Science of Guizhou Higher Education Institutes, Guiyang, China; 6 Department of Laboratory Medicine, Guizhou Kweichow Moutai Hospital, Moutai 564501, China

**Keywords:** Oncology

## Abstract

Colorectal cancer (CRC) is a leading cause of cancer-related mortality worldwide, and early detection remains critical for improving patient outcomes. Serum tumor markers carcino-embryonic antigen (CEA) and carbohydrate antigen 19-9 (CA19-9) are widely used in CRC diagnosis, yet their individual performance is limited. This meta-analysis systematically evaluated the diagnostic performance of CEA, CA19-9, and their combination across 95 studies (22,821 subjects). The combined CEA+CA19-9 testing achieved a pooled sensitivity of 0.585, specificity of 0.877, and diagnostic odds ratio of 9.99—representing improvements of 74% and 138% over CEA and CA19-9 alone, respectively. The area under the summary receiver operating characteristic (SROC) curve reached 0.783 for combined testing. Clinical utility analyses using Fagan’s nomogram and likelihood ratio scatterplots confirmed the superiority of the multi-marker strategy. These findings provide evidence-based support for implementing combined tumor marker testing in clinical CRC diagnosis.

## Highlights

• A meta-analysis of the combined detection of colorectal cancer by CEA and CA19-9
• Combination test improves diagnostic efficiency compared to single tumor markers
• No evidence of difference between CEA and CA19-9
• Assay methodology as a source of heterogeneity

## Graphical abstract

**Figure:**
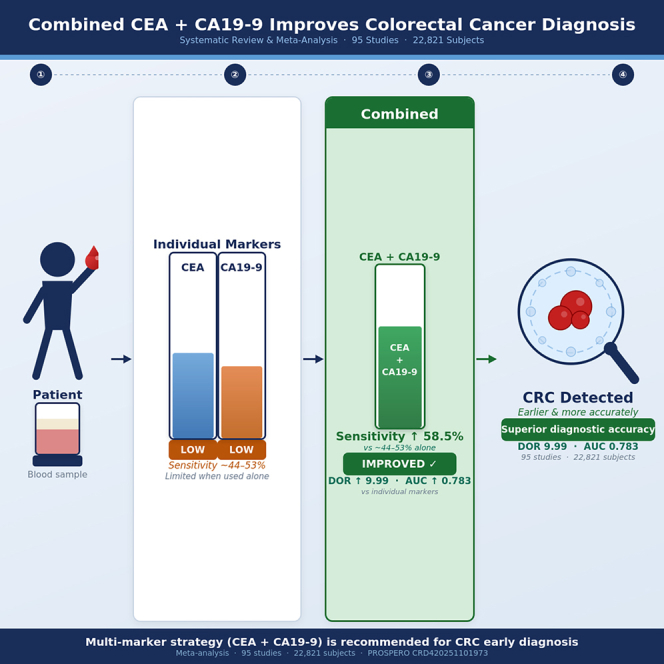

## Introduction

Colorectal cancer (CRC) ranks among the most prevalent cancers globally, with an incidence of 1–2 million new cases and approximately 700,000 fatalities annually. It follows lung, liver, and stomach cancers, thereby positioning itself as the third most common cancer and the fourth leading cause of cancer-related mortality. In terms of gender distribution, CRC is the second most prevalent cancer in women (9.2%) and the third most prevalent in men (10%). CRC encompasses a spectrum of tumors that arise from a confluence of somatic mutations, gene fusions, genetic instability, epigenetic alterations, and various other factors.^1^^,^^2^^,^^3^ Notably, around 70% of metastatic CRC originate from adenomatous polyps, while 25–30% are attributed to sessile serrated lesions.^4^

CRC is influenced by a multitude of risk factors, encompassing environmental and dietary elements, personal habits, and familial genetics.^5^ This complexity renders primary prevention through the elimination of causative factors particularly challenging. Research indicates that CRC typically progresses through a sequence of stages: adenoma, atypical hyperplasia, and carcinoma, with approximately 85% of CRCs originating from adenomas. The transition from adenomas to stage IV invasive cancer generally spans a period of 10–15 years, and it is noteworthy that 75% of CRCs can be averted through the endoscopic removal of adenomas.^6^ However, the invasive nature, intricacy, and challenges associated with bowel endoscopic surgery limit the overall benefits of such procedures for the population in terms of preclinical CRC prevention. Clinicians have observed that the 5-year survival rate for stage I CRC can reach as high as 90%, whereas it plummets to below 8% at stage IV.^7^ Given that the progression from epithelial hyperplasia to carcinoma occurs over a relatively extended time frame, there exists a significant window of opportunity for clinical intervention.^8^^,^^9^^,^^10^ Consequently, early screening, diagnosis, and treatment of CRC are crucial for enhancing the long-term prognosis of affected individuals.

There are several methods available for the detection of CRC, with the most commonly employed screening techniques including fecal testing, imaging, endoscopy, and tumor markers. However, fecal testing is characterized by low specificity, imaging incurs significant costs, and endoscopy often suffers from poor patient compliance. Furthermore, the presence of clinical symptoms indicates that CRC may have progressed to a malignant stage, highlighting the urgent need for effective early detection methods for this disease.^11^ CRC tumor markers are highly predictive and can identify changes in the content of colon tumors prior to their manifestation, thereby providing a foundation for the early diagnosis of colon tumors. Additionally, as a novel biomarker approach, this method is economical, convenient, effective, non-invasive, and possesses other advantages that enhance its acceptability among patients.^12^ Currently, serum tumor markers such as carcino-embryonic antigen (CEA) and carbohydrate antigen 19-9 (CA19-9) are the most frequently utilized laboratory diagnostic tools and have been extensively applied in the early diagnosis and prognosis of CRC^13^^,^^14^^,^^15^ Nevertheless, in clinical practice, reliance on a single serum tumor marker does not suffice for the early detection of CRC.^16^^,^^17^ The pursuit of tumor markers with high specificity and sensitivity, along with their combination with other tumor markers for the early detection of CRC, represents two significant trends in the advancement of diagnostic and therapeutic technologies for this malignancy. However, the clinical diagnostic value of CEA, CA19-9, and their combination for CRC remains inconclusive; some studies^18^^,^^19^^,^^20^ have indicated that these markers do not enhance the sensitivity of diagnostic tests, while others have suggested that they may improve the accuracy and sensitivity of CRC diagnosis.

Therefore, this study aims to perform a meta-analysis to assess the diagnostic efficacy of the combined use of CEA and CA19-9 in the early detection of CRC.

Differences from previous meta-analyses include: (1) this study is the first to simultaneously include 95 studies (the largest previous sample size was 68 studies), covering populations from multiple regions including Asia, Europe, and America; (2) it is the first to combine the HSROC model with clinical utility analysis (Fagan nomogram, decision curve), rather than merely reporting pooled effect sizes.

Therefore, this study aims to perform a meta-analysis to assess the diagnostic efficacy of the combined use of CEA and CA19-9 in the early detection of CRC.

## Results

### Literature search results

**Figure 1:**
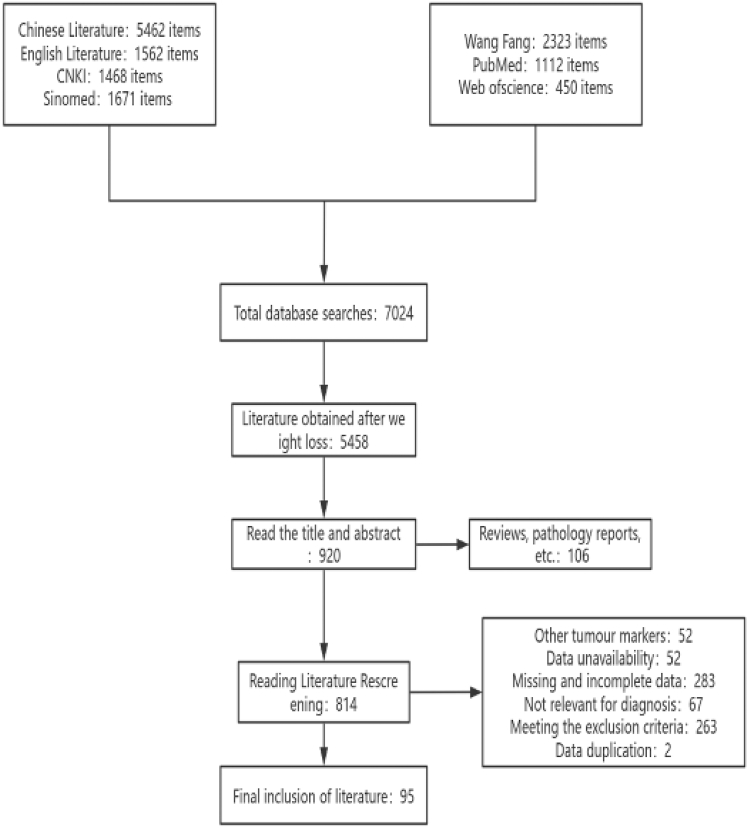
Literature review flowchart

Based on the established search strategy, a total of 7,024 documents were retrieved. Following a systematic screening process in accordance with the specified criteria, 95 documents were selected for final inclusion, all of which were case-control studies. The flowchart illustrating the literature screening process is presented in Figure 1.

### Literature characteristics

The fundamental characteristics of the literature included in this review are presented in Tables 1 and 2. The studies encompassed a total of 22,821 subjects. In total, 95 articles were included, comprising 65 articles in Chinese and 30 articles in English.

**Table 1:** Characteristics of individual tumor marker assays

Literature inclusion	CEA			CA19-9		
	Methods	Threshold (ng/mL)	TP/FP/FN/Tn	Methods	Threshold (ng/mL)	TP/FP/FN/Tn
Bhawna Bagaria et al. 2013^21^	ELISA	3.4	38/0/12/50	ELISA	35	13/0/37/50
P Fan et al. 2017^22^	ELISA	10	45/101/70/239	ELISA	27	55/105/60/235
L.Fermindez-Fermindez et al. 1995^23^	CLIA	5.6	59/4/68/66	CLIA	51	26/4/101/66
Juan-Juan Gao et al. 2022^24^	ELISA	5	56/4/54/87	ELISA	39	17/3/93/88
Yanfeng Gao et al. 2018^25^	ELISA	3.4	130/15/149/59	ELISA	39	41/8/238/66
Cuijuan Hao et al. 2021^26^	CLIA	5	90/12/66/40	CLIA	37	81/11/75/41
László Herszényi et al. 2008^27^	ELISA	4	16/4/40/31	ELISA	37	10/3/46/32
Shenghuai Hou et al. 2023^28^	ELISA	3	509/18/471/852	ELISA	20	343/79/637/791
Zhengyuan Huang et al. 2022^29^	CLIA	5	79/1/148/122	CLIA	37	36/0/191/123
P Kuusela et al. 1991^30^	RIA	3	43/6/34/24	RIA	37	26/0/51/30
Nanyang Li et al. 2023^31^	ECLI	5	21/7/9/23	ECLI	35	21/7/9/23
Pingxia Lu et al. 2022^32^	ECLI	5	270/5/346/117	ECLI	37	92/5/524/117
Franco Lumachi et al. 2012^33^	ELISA	4.9	44/10/58/89	ELISA	32.6	22/72/80/27
Barbara Mroczko et al. 2007^34^	MEIA	5	51/0/81/65	MEIA	30	25/0/107/65
Shufang Ning et al.2018^35^	ECLI	6.5	108/7/59/68	ECLI	37	103/9/64/66
Jianhong Peng et al. 2018^36^	ECLI	5	173/7/253/87	ECLI	35	84/2/341/92
F Safi et al. 1987^37^	RIA	3	61/50/32/186	RIA	37	49/15/44/221
Cheng Shi et al. 2019^38^	ECLI	1.83	174/51/37/52	ECLI	11	132/72/79/31
Swarnima Singh et al. 2020^39^	ECLI	6.5	34/6/19/47	ECLI	37	32/6/21/47
Yanfang Song et al. 2018^40^	ECLI	5	321/0/462/331	ECLI	37	133/0/650/331
Y F Song et al. 2017^41^	ELISA	5	58/7/79/165	ELISA	37	27/4/110/168
Ratko Tomašević et al. 2016^42^	CLIA	5	139/45/42/146	CLIA	35.4	125/51/56/140
Chun-Xiao Wang et al. 2021^43^	ECLI	5.2	60/4/76/46	ECLI	37	19/1/117/49
Jia Wang et al. 2014^44^	ECLI	6.5	40/5/73/84	ECLI	39	25/4/88/85
Li Xie et al. 2018^45^	ECLI	5.2	43/47/80/78	ELISA	39	22/56/101/69
Weili Yang et al. 2018^46^	CLIA	5	20/20/30/30	CLIA	37	27/4/23/46
Dongdong Yu et al. 2021^47^	CLIA	5	49/10/84/269	CLIA	37	16/0/117/279
Yixiang Zhan et al. 2023^48^	ECLI	5	164/385/281/57	ECLI	39	418/18/27/424
Shiyan Zhang et al. 2015^49^	ECLI	5	89/12/49/99	ECLI	37	65/11/73/100
Xianwen Zhao et al. 2005^50^	ELISA	2.38	73/7/61/193	ELISA	27.39	45/15/89/185
Min Ai et al. 2020^51^	ECLI	5	58/37/17/38	ECLI	34	29/30/46/45
Ji Ban et al. 2020^52^	ELISA	5	83/20/17/81	ELISA	35	78/19/22/82
Shangdang Cai et al. 2016^53^	ECLI	5.14	28/4/18/33	ECLI	37.01	17/7/29/30
Lihui Cao et al. 2019^54^	CLIA	5	31/15/19/35	CLIA	37	34/18/16/32
Yaping Cao et al. 2021^55^	ECLI	5	576/89/329/335	ECLI	35	359/52/546/372
Longfei Zeng et al. 2017^56^	ECLI	6	152/69/79/202	ECLI	37	148/28/83/243
Junfeng Chang et al. 2014^57^	CLIA	3.4	45/7/25/63	CLIA	27	24/8/46/62
Bin Chen et al. 2010^58^	ECLI	5	68/2/129/64	ECLI	37	21/2/186/64
Deyu Chen et al. 2008^59^	MEIA	5	35/2/67/38	MEIA	37	38/1/64/39
Feiying Chen et al. 2017^60^	CLIA	10	78/23/79/144	CLIA	37	75/26/82/141
Jie Chen et al. 2005^61^	MEIA	5	22/11/26/21	MEIA	37	18/12/30/20
Jinjin Chen et al. 2021^62^	CLIA	5	57/25/23/140	CLIA	37	48/16/32/149
Kaijie Chen et al. 2005^63^	MEIA	5	21/10/24/20	MEIA	37	17/11/28/19
Liwan Chen et al. 2021^64^	CLIA	5	52/34/53/66	CLIA	35	51/33/54/67
Shuying Chen et al. 2017^65^	ECLI	2.41	64/1/17/29	ECLI	15.05	51/0/30/30
Yanping Chen et al. 2017^66^	CLIA	5	46/6/54/94	CLIA	39	40/15/60/85
Yuxue Deng et al. 2017^67^	ECLI	4.3	23/9/27/62	ECLI	27	19/19/31/52
Yujiao Ding et al. 2023^68^	ECLI	6	24/11/6/19	ECLI	37	21/9/9/21
Jinlang Du et al.2014^69^	ECLI	5	43/4/48/29	ECLI	35	59/6/32/27
Ping Fan et al. 2017^70^	ECLI	10	45/101/70/239	ECLI	27	55/105/60/235
Xiaojing Fan et al. 2019^71^	ECLI	5	26/29/24/76	ECLI	37	23/32/27/73
Chenxi Feng et al. 2022^72^	ECLI	5	48/12/27/29	ECLI	35	48/18/27/23
Yixi Feng et al.2017^73^	CLIA	5	21/2/29/48	CLIA	35	13/2/37/48
Qunhao Gu et al. 2012^74^	MEIA	5	18/4/27/26	MEIA	37	15/3/30/27
Yahui Guo et al. 2022^75^	ECLI	5	74/38/23/66	ECLI	35	42/13/55/91
Sui He et al. 2004^76^	ECLI	4.6	26/2/30/28	ECLI	27	20/1/36/29
Hualin He et al. 2019^77^	ECLI	5	119/9/60/98	ECLI	39	114/18/65/89
Ying Huang et al. 2017^78^	CLIA	5	39/39/21/21	CLIA	35	36/18/24/42
Mengying Jiang et al. 2023^79^	ELISA	5	24/13/18/55	ELISA	27	17/0/25/68
Kai Lei et al. 2019^80^	ECLI	10	40/34/33/36	ECLI	37	30/27/43/43
Xiong Lei et al. 2011^81^	ECLI	6.5	23/6/22/48	ECLI	27	18/4/27/41
Chunguang Li et al. 2020^82^	CLIA	5	29/2/13/38	CLIA	37	24/3/18/37
Peng Li et al. 2022^83^	ECLI	10	60/21/40/29	ECLI	37	59/20/41/30
Shijun Li et al. 2010^84^	MEIA	5	60/26/98/134	MEIA	37	43/38/115/122
Zhiguo Li et al. 2021^85^	CLIA	10	35/8/19/38	CLIA	37	33/7/21/39
Yan Liang et al. 2006^86^	ECLI	3.4	32/9/45/25	ECLI	22	34/11/43/23
Li Lin et al. 2020^87^	CLIA	10	32/4/28/96	CLIA	35	31/7/29/93
Chanrong Lu et al. 2012^88^	ECLI	5	52/5/39/53	ECLI	37	59/20/32/38
Shaolei Luo et al. 2017^89^	Protein chip	5	51/3/83/290	Protein chip	35	37/5/97/288
Zhongchuan Lv et al. 2013^90^	CLIA	5.9	88/17/72/83	CLIA	37	59/22/101/78
Junkong Ma et al. 2022^91^	ECLI	5	26/4/29/51	ECLI	27	35/10/20/45
Lijuan Pan et al. 2020^92^	ELISA	5	22/45/23/78	ELISA	37	21/72/24/51
Fenghua Pei et al. 2010^93^	ELISA	4	13/0/23/183	ELISA	37	6/0/30/183
Zhengjie Qu et al. 2021^94^	ECLI	2.805	52/5/28/45	ECLI	39	28/10/52/40
Mengzhen Shi et al. 2020^95^	ECLI	3.4	14/6/20/44	ECLI	27	13/7/21/43
Guo Wang et al. 1998^96^	RIA	15	19/21/23/171	RIA	37	26/14/16/178
Kun Wang et al. 2017^97^	CLIA	7.2	46/3/55/76	CLIA	27	41/3/60/76
Yanyan Wang et al. 2019^98^	CLIA	5	49/27/29/72	CLIA	37	46/21/32/78
Yisheng Wei et al. 2006^99^	Protein chip	5	25/1/57/40	Protein chip	35	28/1/54/40
Yi Wu et al. 2022^100^	ECLI	5	25/4/40/65	ECLI	27	33/6/32/63
Mingyue Xiang.2007^101^	MEIA	5	37/1/42/49	MEIA	37	39/2/40/48
Qiong Xu et al. 2022^102^	CLIA	5	40/11/32/61	CLIA	35	27/12/45/60
Qinghui Yan et al. 2016^103^	CLIA	5	24/1/12/29	CLIA	32.4	25/2/11/28
Liping Yang et al. 2020^104^	CLIA	3.73	74/2/64/48	CLIA	14.40	67/8/71/42
Maowen Yu et al. 2018^105^	ECLI	5	133/30/59/50	ECLI	39	131/26/61/54
Guoliang Zhang et al. 2023^106^	CLIA	3.13	42/8/33/43	ECLI	12.83	34/0/41/51
Guo Yu et al. 2011^107^	ECLI	5	17/4/55/31	ECLI	27	25/2/47/33
Hua Zhang et al. 2020^108^	MEIA	5.96	59/3/71/47	MEIA	21.1	63/10/67/40
Wanzhong Zhang et al. 2007^109^	RIA	5	23/2/7/28	RIA	37	16/0/14/30
Ming Zhao et al. 2005^110^	RIA	15	23/1/26/37	RIA	37	27/2/22/36
Ming Zhao et al. 2006^111^	RIA	15	45/8/11/79	RIA	37	39/9/27/78
Aiqing Zhou et al. 2007^112^	ECLI	3.4	53/16/36/91	ECLI	37	51/10/38/97
Xujun Zhou et al. 2014^113^	ECLI	3.4	29/3/15/27	ECLI	27	31/2/13/28
Meiiqin Zhu et al. 2016^114^	CLIA	5	33/13/28/104	CLIA	37	28/10/33/107
Zili Zhu et al. 2006^115^	RIA	20	32/10/35/45	RIA	37	36/11/31/44

CLIA: chemiluminescence immunoassay; ECLI: electrochemiluminescence immunoassay; ELISA: enzyme-linked immunosorbent assay; RIA: radioimmunoassay; MEIA: microbial enzyme immunoassay; protein chip; TP: true positive; FP: false positive; FN: false negative; Tn: true negative.

**Table 2:** Characteristics of combined tumor marker assays

Study inclusion	Vintages	CEA+CA19-9(TP/FP/FN/Tn)
Bhawna Bagaria.et a^21^	2013	36/0/14/50
P Fan et al.^22^	2017	66/95/49/245
L.Fernández-Fernández et al.^23^	1995	65/7/62/63
Yanfeng Gao et al.^25^	2018	138/16/141/58
László Herszényi et al.^27^	2008	18/2/38/33
Shenghuai Hou et al.^28^	2023	656/140/324/730
Zhengyuan Huang et al.^29^	2022	169/21/58/102
Pingxia Lu et al.^32^	2022	343/10/273/112
Barbara Mroczko et al.^34^	2007	61/0/71/65
F Safi et al.^37^	1987	70/22/23/214
Yanfang Song et al.^40^	2018	392/10/391/321
Y F Song et al.^41^	2017	121/68/16/104
Ratko Tomašević et al.^42^	2016	133/32/48/159
Dongdong Yu et al.^47^	2021	52/10/81/269
Shi-Yan Zhang et al.^49^	2015	98/19/40/92
Xian-wen Zhao et al.^50^	2005	79/17/55/183
Yaping Cao et al.^55^	2021	560/72/345/352
Longfei Zeng et al.^56^	2017	164/31/67/240
Bin Chen et al.^58^	2010	74/2/120/64
Deyu Chen et al.^59^	2008	49/2/53/38
Jinjin Chen et al.^62^	2021	61/25/19/140
Yanping Chen et al.^66^	2017	57/18/43/82
Yuxue Deng et al.^67^	2017	19/11/31/60
Ping Fan et al.^70^	2017	66/95/49/245
Xiaojing Fan et al.^71^	2019	41/16/9/89
Sui He et al.^76^	2004	30/3/26/27
Hualin He et al.^77^	2019	117/4/62/103
Xiong Lei et al.^81^	2011	32/4/13/50
Yan Liang et al.^86^	2006	34/11/43/23
Shaolei Luo et al.^89^	2017	68/8/66/285
Fenghua Pei et al.^93^	2010	14/0/22/183
Yisheng Wei et al.^99^	2006	39/2/43/39
Mingyue Xiang et al.^101^	2007	46/3/33/47
Guo Yu et al.^107^	2011	23/6/49/29
Ming Zhao et al.^110^	2005	38/3/11/35
Aiqing Zhou et al.^112^	2007	66/21/23/86

TP: true positive; FP: false positive; FN: false negative; Tn: true negative.

### Risk of bias assessment

Risk of bias assessment was performed using the QUADAS-2 tool across four domains: patient selection, index test, reference standard, and flow and timing. The results are presented in Table 3. The majority of studies were rated as low risk across all domains. High risk of bias in patient selection was noted in 13 studies, while unclear risk was noted in the gold standard domain for several earlier publications. All studies were consistently rated as low risk for the index test domain.

**Table 3:** Risk of bias assessment of included studies

Study inclusion	Assessment of bias				Suitability		
	Case selection	Tests to be evaluated	Gold Standard	Case flow and progress	Case selection	Tests to be evaluated	Gold Standard
Bhawna Bagaria et al. 2013^21^	low risk	low risk	low risk	low risk	low risk	low risk	low risk
P Fan et al. 2017^22^	low risk	low risk	low risk	low risk	low risk	low risk	low risk
L.Fermindez-Fermindez et al. 1995^23^	low risk	low risk	unknown	low risk	low risk	low risk	unknown
Juan-Juan Gao et al. 2022^24^	low risk	low risk	low risk	low risk	low risk	low risk	low risk
Yanfeng Gao et al. 2018^25^	unknown	low risk	unknown	unknown	unknown	low risk	unknown
Cuijuan Hao et al. 2021^26^	low risk	low risk	low risk	unknown	low risk	low risk	low risk
László Herszényi et al. 2008^27^	high risk	low risk	low risk	unknown	low risk	low risk	low risk
Shenghuai Hou et al. 2023^28^	low risk	low risk	unknown	unknown	low risk	low risk	unknown
Zhengyuan Huang et al. 2022^29^	low risk	low risk	unknown	unknown	low risk	low risk	unknown
P Kuusela et al. 1991^30^	high risk	low risk	unknown	unknown	high risk	low risk	unknown
Nanyang Li et al.2023^31^	low risk	low risk	low risk	low risk	low risk	low risk	low risk
Pingxia Lu et al. 2022^32^	low risk	low risk	low risk	low risk	low risk	low risk	low risk
Franco Lumachi et al. 2012^33^	unknown	low risk	unknown	unknown	low risk	low risk	unknown
Barbara Mroczko et al. 2007^34^	unknown	low risk	unknown	unknown	unknown	low risk	unknown
Shufang Ning et al. 2018^35^	low risk	low risk	unknown	unknown	low risk	low risk	unknown
Jianhong Peng et al. 2018^36^	unknown	low risk	unknown	unknown	unknown	low risk	unknown
F Safi et al. 1987^37^	unknown	low risk	unknown	unknown	unknown	low risk	unknown
Cheng Shi et al. 2019^38^	low risk	low risk	low risk	low risk	low risk	low risk	low risk
Swarnima Singh et al. 2020^39^	unknown	low risk	unknown	unknown	unknown	low risk	unknown
Yanfang Song et al. 2018^40^	low risk	low risk	unknown	unknown	low risk	low risk	unknown
Y F Song et al. 2017^41^	high risk	low risk	unknown	unknown	unknown	low risk	unknown
Ratko Tomašević et al. 2016^42^	low risk	low risk	low risk	low risk	low risk	low risk	low risk
Chun-Xiao Wang et al. 2021^43^	unknown	low risk	unknown	unknown	low risk	low risk	unknown
Jia Wang et al. 2014^44^	low risk	low risk	low risk	low risk	low risk	low risk	low risk
Li Xie et al. 2018^45^	low risk	low risk	unknown	unknown	low risk	low risk	unknown
Weili Yang et al. 2018^46^	low risk	low risk	low risk	low risk	low risk	low risk	low risk
Dongdong Yu et al. 2021^47^	low risk	low risk	low risk	low risk	low risk	low risk	low risk
Yixiang Zhan et al. 2023^48^	unknown	low risk	low risk	low risk	low risk	low risk	low risk
Shi-Yan Zhang et al. 2015^49^	low risk	low risk	high risk	low risk	low risk	low risk	High risk
Xian-wen Zhao et al. 2005^50^	low risk	low risk	unknown	unknown	low risk	low risk	unknown
Min Ai et al. 2020^51^	low risk	low risk	low risk	low risk	low risk	low risk	low risk
Ji Ban et al. 2020^52^	unknown	low risk	unknown	unknown	low risk	low risk	unknown
Shangdang Cai et al. 2016^53^	low risk	low risk	unknown	unknown	low risk	low risk	unknown
Lihui Cao et al. 2019^54^	low risk	low risk	unknown	unknown	low risk	low risk	unknown
Yaping Cao et al. 2021^55^	unknown	low risk	low risk	low risk	low risk	low risk	low risk
Longfei Zeng et al. 2017^56^	unknown	low risk	low risk	low risk	low risk	low risk	low risk
Junfeng Chang et al. 2014^57^	low risk	low risk	low risk	low risk	low risk	low risk	low risk
Bin Chen et al. 2010^58^	low risk	low risk	low risk	low risk	low risk	low risk	low risk
DeYu Chen et al. 2008^59^	unknown	low risk	low risk	low risk	low risk	low risk	low risk
Feiying Chen et al. 2017^60^	high risk	low risk	low risk	low risk	low risk	low risk	low risk
Jie Chen et al. 2005^61^	unknown	low risk	low risk	low risk	low risk	low risk	low risk
Jinjin Chen et al. 2021^62^	unknown	low risk	low risk	low risk	low risk	low risk	low risk
Kaijie Chen et al. 2005^63^	low risk	low risk	low risk	low risk	low risk	low risk	low risk
Liwan Chen et al. 2021^64^	low risk	low risk	high risk	unknown	low risk	low risk	high risk
Shuying Chen et al. 2017^65^	low risk	low risk	low risk	low risk	low risk	low risk	low risk
Yanping Chen et al. 2017^66^	low risk	low risk	low risk	low risk	low risk	low risk	low risk
Yuxue Deng et al. 2017^67^	low risk	low risk	low risk	low risk	low risk	low risk	low risk
Yujiao Ding et al. 2023^68^	low risk	low risk	low risk	low risk	low risk	low risk	low risk
Jinlang Du et al. 2014^69^	low risk	low risk	low risk	low risk	low risk	low risk	low risk
Ping Fan et al. 2017^70^	low risk	low risk	low risk	low risk	low risk	low risk	low risk
Xiaojing Fan et al. 2019^71^	low risk	low risk	low risk	low risk	low risk	low risk	low risk
Chenxi Feng et al. 2022^72^	low risk	low risk	low risk	low risk	low risk	low risk	low risk
Yixi Feng et al. 2017^73^	low risk	low risk	unknown	unknown	low risk	low risk	unknown
Qunhao Gu et al. 2012^74^	low risk	low risk	low risk	low risk	low risk	low risk	low risk
Yahui Guo et al. 2022^75^	low risk	low risk	low risk	low risk	low risk	low risk	low risk
Sui He et al. 2004^76^	low risk	low risk	low risk	low risk	low risk	low risk	low risk
Hualin He et al. 2019^77^	low risk	low risk	low risk	low risk	low risk	low risk	low risk
Ying Huang et al. 2017^78^	low risk	low risk	low risk	low risk	low risk	low risk	low risk
Mengying Jiang et al. 2023^79^	low risk	low risk	low risk	low risk	low risk	low risk	low risk
Kai Lei et al. 2019^80^	low risk	low risk	low risk	low risk	low risk	low risk	low risk
Xiong Lei et al. 2011^81^	low risk	low risk	low risk	low risk	low risk	low risk	low risk
Chunguang Li et al. 2020^82^	low risk	low risk	low risk	low risk	low risk	low risk	low risk
Peng Li et al. 2022^83^	low risk	low risk	low risk	low risk	low risk	low risk	low risk
Shijun Li et al. 2010^84^	unknown	low risk	low risk	low risk	low risk	low risk	low risk
Zhiguo Li et al. 2021^85^	low risk	low risk	low risk	low risk	low risk	low risk	low risk
Yan Liang et al. 2006^86^	high risk	low risk	low risk	low risk	low risk	low risk	low risk
Li Lin et al. 2020^87^	low risk	low risk	low risk	low risk	low risk	low risk	low risk
Chanrong Lu et al. 2012^88^	low risk	low risk	low risk	low risk	low risk	low risk	low risk
Shaolei Luo et al. 2017^89^	low risk	low risk	low risk	low risk	low risk	low risk	low risk
Zhongchuan Lv et al. 2013^90^	low risk	low risk	low risk	low risk	low risk	low risk	low risk
Junkong Ma et al. 2022^91^	low risk	low risk	unknown	unknown	low risk	low risk	unknown
Lijuan Pan et al. 2020^92^	low risk	low risk	low risk	low risk	low risk	low risk	low risk
Fenghua Pei et al. 2010^93^	low risk	low risk	low risk	low risk	low risk	low risk	low risk
Zhengjie Qu et al. 2021^94^	high risk	low risk	low risk	low risk	low risk	low risk	low risk
Mengzhen Shi et al. 2020^95^	low risk	low risk	low risk	low risk	low risk	low risk	low risk
Guo Wang et al. 1998^96^	high risk	low risk	low risk	low risk	low risk	low risk	low risk
Kun Wang et al. 2017^97^	low risk	low risk	low risk	low risk	low risk	low risk	low risk
Yanyan Wang et al. 2019^98^	low risk	low risk	low risk	low risk	low risk	low risk	low risk
Yisheng Wei et al. 2006^99^	low risk	low risk	low risk	low risk	low risk	low risk	low risk
Yi Wu et al. 2022^100^	high risk	low risk	low risk	low risk	low risk	low risk	low risk
Mingyue Xiang.2007^101^	low risk	low risk	low risk	low risk	low risk	low risk	low risk
Qiong Xu et al. 2022^102^	low risk	low risk	low risk	low risk	low risk	low risk	low risk
Qinghui Yan et al. 2016^103^	low risk	low risk	low risk	low risk	low risk	low risk	low risk
Liping Yang et al. 2020^104^	low risk	low risk	low risk	low risk	low risk	low risk	low risk
Maowen Yu et al. 2018^105^	low risk	low risk	low risk	low risk	low risk	low risk	low risk
Guoliang Zhang et al. 2023^106^	low risk	low risk	low risk	low risk	low risk	low risk	low risk
Guo Yu et al. 2011^107^	low risk	low risk	low risk	low risk	low risk	low risk	low risk
Hua Zhang et al. 2020^108^	low risk	low risk	low risk	low risk	low risk	low risk	low risk
Wanzhong Zhang et al. 2007^109^	high risk	low risk	low risk	low risk	low risk	low risk	low risk
Ming Zhao et al. 2005^110^	high risk	low risk	unknown	unknown	low risk	low risk	unknown
Ming Zhao et al. 2006^111^	high risk	low risk	low risk	low risk	low risk	low risk	low risk
Aiqing Zhou et al. 2007^112^	high risk	low risk	low risk	low risk	low risk	low risk	low risk
Xujun Zhou et al. 2014^113^	low risk	low risk	low risk	low risk	low risk	low risk	low risk
Meiqin Zhu et al. 2016^114^	high risk	low risk	low risk	low risk	low risk	low risk	low risk
Zili Zhu et al. 2006^115^	low risk	low risk	low risk	low risk	low risk	low risk	low risk

### Main research findings

Quantitative analysis indicated that the diagnostic odds ratio (DOR) for the combined test reached 9.99, reflecting significant increases of 74% and 138% compared to CEA alone (5.73) and CA19-9 alone (4.19), respectively.

The results of the heterogeneity assessment consistently demonstrated high heterogeneity characteristics among the studies included in the meta-analysis. The inter-study heterogeneity indicators for all three testing strategies significantly surpassed critical thresholds: I^2^ values were recorded at 91.2% for CEA testing (95 studies), 91.4% for CA19-9 testing (also 95 studies), and 91.2% for the combined CEA+CA19-9 testing (36 studies).

**Figure 2:**
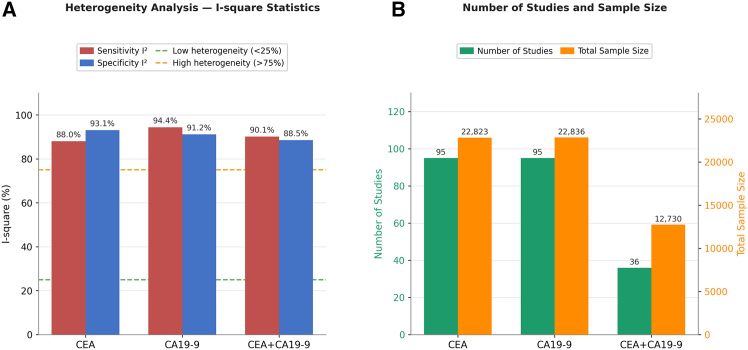
Heterogeneity analysis

Analysis of sample characteristics revealed that individual studies on CEA and CA19-9 testing had substantial sample sizes, with case groups comprising 12,514 and 12,533 subjects, and control groups consisting of 10,307 and 10,298 subjects, respectively. In contrast, studies involving combined detection exhibited relatively smaller sample sizes, with 6,906 cases and 5,823 controls (Figure 2).

An in-depth analysis of the performance characteristics across multiple diagnostic dimensions revealed that, in terms of sensitivity, the combination of CEA and CA19-9 exhibited optimal performance, with a pooled sensitivity of 0.585 (95% CI: 0.539–0.634). This represents a 10.0% improvement over CEA alone (0.532, 95% CI: 0.506–0.558) and a substantial 32.7% enhancement compared to CA19-9 alone (0.441, 95% CI: 0.408–0.476). The evaluation of specificity similarly demonstrated the advantages of combined testing, yielding a specificity of 0.877 (95% CI: 0.845–0.908). This indicates a 5.2% improvement over CEA alone (0.834, 95% CI: 0.801–0.862) and a 4.3% enhancement compared to CA19-9 alone (0.841, 95% CI: 0.809–0.871).

Analyses of the likelihood ratio and DOR further substantiated the clinical value of the combined testing strategy. The positive likelihood ratio of the CEA and CA19-9 combination reached 4.73, significantly surpassing that of CEA alone (3.21) and CA19-9 alone (2.78), indicating that a positive result from the combined test provides stronger confirmatory power for disease diagnosis. Concurrently, the negative likelihood ratio of the combined test decreased to 0.47, lower than that of CEA (0.56) and CA19-9 (0.66), demonstrating a higher reliability of negative results in ruling out the disease.

The DOR, serving as a comprehensive indicator of diagnostic performance, clearly illustrates the significant advantages of combined testing. The DOR for the CEA+CA19-9 combination reached 9.99, indicating a 74.3% improvement over CEA alone (5.73) and a 138.4% enhancement compared to CA19-9 alone (4.19).

The area under the receiver operating characteristic curve (AUC) analysis indicated that the combined testing exhibited an AUC of 0.581 (95% CI: 0.500–0.581), representing an improvement relative to CEA alone (0.482, 95% CI: 0.462–0.503) and CA19-9 alone (0.444, 95% CI: 0.391–0.444), further validating the efficacy of the multi-marker combination strategy in enhancing diagnostic performance (Table 4).

**Table 4:** Pooled diagnostic performance of the three testing strategies

Testing Strategy	CEA	CA19-9	CEA+CA19-9
Number of Studies	95	95	36
Total Cases	12514	12533	6906
Total Controls	10307	10298	5823
Pooled Sensitivity	0.532	0.441	0.585
Sensitivity 95% CI	[0.506,0.558]	[0.408,0.476]	[0.539,0.634]
Pooled Specificity	0.834	0.841	0.877
Specificity 95% CI	[0.801,0.862]	[0.809,0.871]	[0.845,0.908]
Positive Likelihood Ratio	3.21	2.78	4.73
Negative Likelihood Ratio	0.56	0.66	0.47
Diagnostic Odds Ratio	5.73	4.19	9.99
AUC 95%CI	[0.462,0.503]	[0.391,0.444]	[0.500,0.581]

### Forest plot analysis

**Figure 3:**
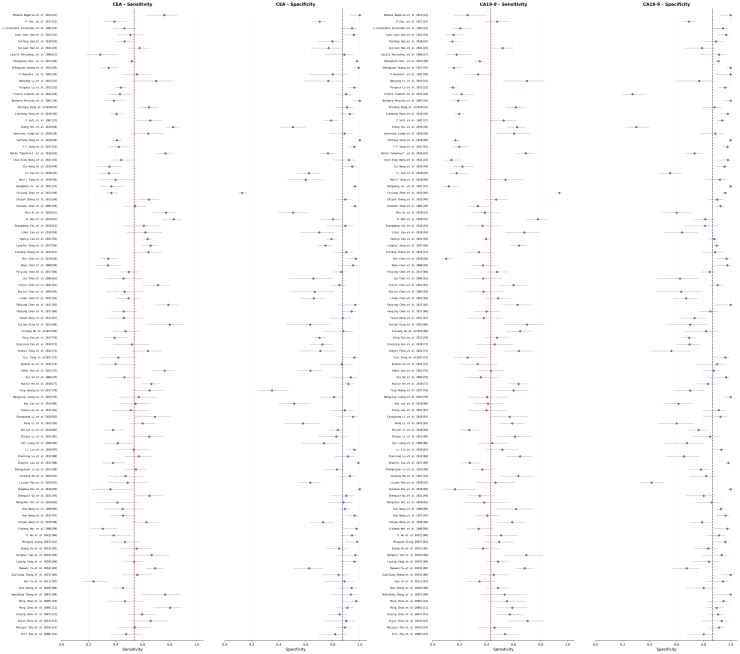
Forest plots for CEA and CA19-9

The forest plot analysis of CA19-9 testing (Figure 3) elucidates the intricate performance characteristics of this biomarker in the diagnosis of CRC. In terms of sensitivity, the 95 included studies exhibit significant inter-study variability, with individual study sensitivity estimates ranging from approximately 0.15 to 0.85. The pooled sensitivity is calculated at 0.565 (95% CI); however, the degree of overlap in the confidence intervals suggests statistical inconsistency among certain study results. Notably, approximately 15–20% of the studies report CA19-9 sensitivity below 0.3, which may be attributable to variations in patient tumor staging, histological types, or detection threshold settings across different investigations. In regard to specificity, CA19-9 testing demonstrates relatively better consistency, with the majority of studies’ specificity estimates concentrated within the 0.7–0.95 range, yielding a pooled specificity of 0.877 (95% CI).

The forest plot for CEA testing (Figure 3) reveals distribution characteristics that are similar to yet distinct from those of CA19-9. In the sensitivity analysis, the individual estimates from the 95 studies also exhibit considerable variation, ranging from approximately 0.2 to 0.8; however, the overall distribution appears more concentrated compared to CA19-9, with a pooled sensitivity of 0.585 (95% CI). The proportion of studies reporting low CEA sensitivity (<0.3) is relatively smaller, accounting for approximately 10–12% of the total, which may suggest that CEA, as a traditional biomarker for CRC, maintains relatively better stability across diverse research settings. In terms of specificity, CEA testing demonstrates good inter-study consistency, with the majority of studies’ specificity estimates distributed within the 0.75–0.95 range, resulting in a pooled specificity of 0.834 (95% CI).

**Figure 4:**
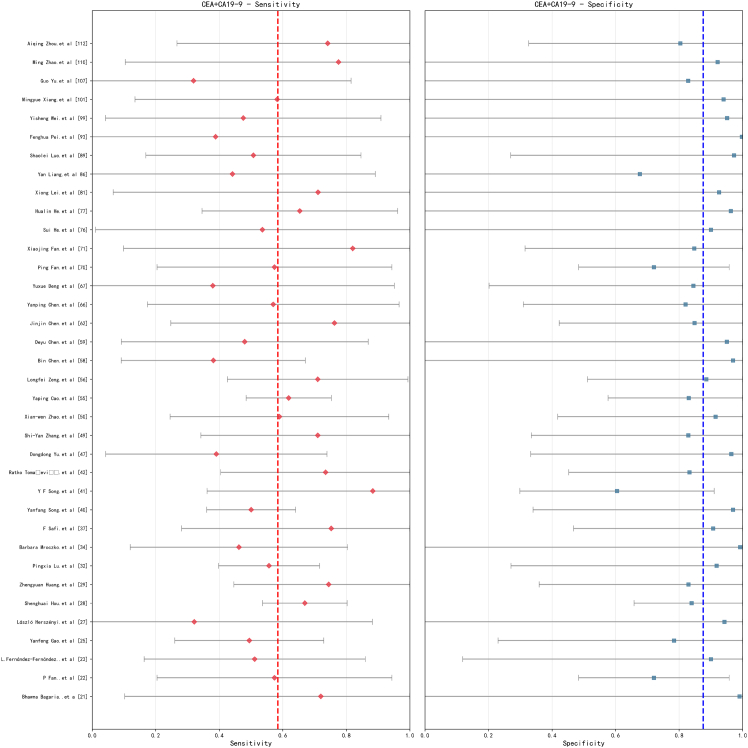
Forest plot for the combined detection of CEA and CA19-9

The forest plot analysis of the combined testing strategy (Figure 4) provides compelling visual evidence for the synergistic effects of multiple biomarkers. An analysis based on 36 studies demonstrates that combined testing exhibits a significant improvement in both sensitivity and specificity compared to individual tests. The sensitivity forest plot reveals that the individual study estimates for combined testing are predominantly distributed within the 0.45–0.75 range, yielding a pooled sensitivity of 0.585 (95% CI). Notably, the proportion of studies with low sensitivity (<0.4) decreases significantly to below 5%. In the evaluation of specificity, combined testing demonstrates optimal performance, with individual study specificity estimates primarily concentrated in the 0.8–0.95 range, and a pooled specificity increasing to 0.877 (95% CI).

### Summary receiver operating characteristic curve analysis

**Figure 5:**
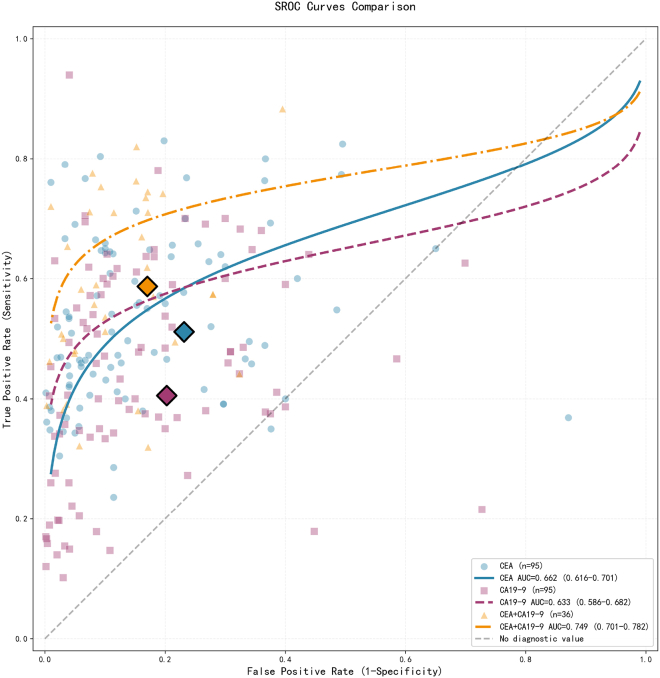
ROC curves

The summary receiver operating characteristic (SROC) curve for CEA testing indicates a moderate-to-low level of diagnostic accuracy, with an AUC of 0.662 (95% CI: 0.618–0.763). In contrast, CA19-9 testing exhibits relatively superior SROC performance, with the AUC rising to 0.725 (95% CI: 0.670–0.780). Furthermore, the SROC curve for the combined testing of CEA and CA19-9 demonstrates optimal diagnostic performance characteristics, achieving an AUC of 0.783 (95% CI: 0.760–0.807), which represents a substantial improvement over individual marker testing (Figure 5).

### Clinical utility assessment

Fagan’s nomogram analysis elucidates the clinical conversion efficiency of three testing strategies across various pre-test probability scenarios. The nomogram for CA19-9 testing indicates that in typical CRC screening settings (with an approximate pre-test probability of 10%), a positive result can elevate the post-test probability to approximately 25–30%, whereas a negative result can diminish the probability to around 5–7%. CEA testing exhibits clinical utility characteristics that are similar to, yet slightly enhanced over, those of CA19-9. Under identical pre-test probability conditions, a positive CEA result can increase the post-test probability to about 30–35%, while a negative result can reduce the probability to approximately 6–8%.

**Figure 6:**
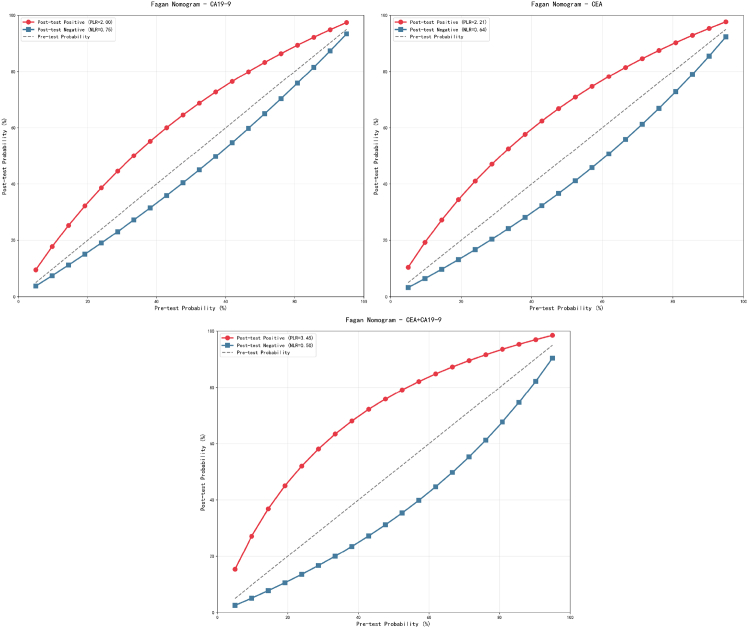
Fagan’s nomogram

The Fagan’s nomogram for the combined CEA+CA19-9 testing illustrates a significantly enhanced clinical utility. Under the same pre-test probability conditions, a positive combined test result can elevate the post-test probability to approximately 40–45%, thereby reaching the clinical threshold for high suspicion, while a negative result can reduce the probability to about 3–5%, thereby approaching the clinical standard for effectively ruling out the condition (Figure 6).

**Figure 7:**
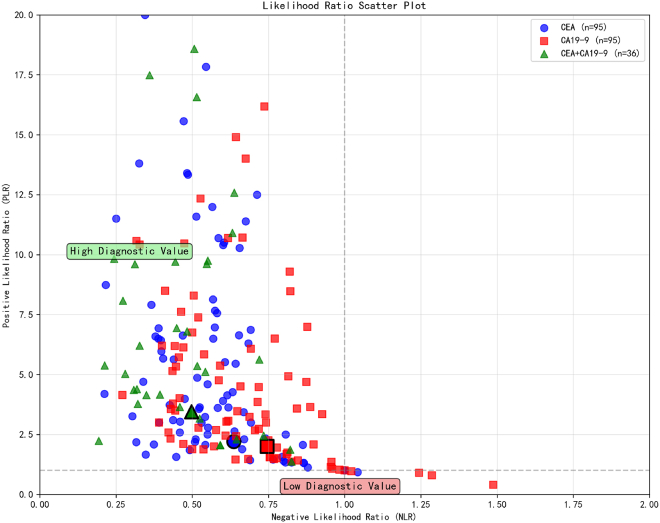
Likelihood ratio scatterplot

The likelihood ratio scatterplot (Figure 7) serves as an intuitive visualization tool for evaluating the clinical discriminative value of the three testing strategies. The analysis indicates that the majority of study data points are situated within the moderate diagnostic value zone (PLR 2–10, NLR 0.1–0.5). Data points pertaining to CEA testing are predominantly clustered within the PLR 2–6 and NLR 0.3–0.7 range, reflecting a moderate-to-low clinical discriminative value. CA19-9 testing exhibits a relatively favorable distribution of likelihood ratio characteristics, with the majority of data points situated within the ranges of PLR 3–8 and NLR 0.2–0.5. The combined testing of CEA and CA19-9 reveals optimal distribution characteristics, with data points predominantly concentrated in the high-value region of PLR 4–10 and NLR 0.1–0.4. Notably, approximately 30–40% of studies utilizing combined testing fulfilled the criteria of PLR>6 and NLR<0.3, in contrast to only 10–15% observed in single-marker testing.

### Quality assessment

**Figure 8:**
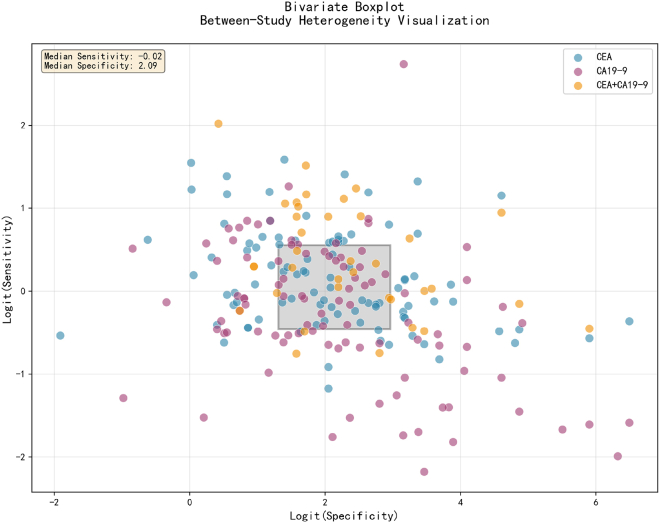
Bivariate boxplot

CEA testing revealed the broadest distribution span along the horizontal axis (ranging from −2 to +7), indicating a significant degree of inter-study variability. The dispersed distribution of data points suggests that CEA test results are influenced by multiple study design factors, thereby necessitating careful consideration of specific testing conditions and patient characteristics in clinical applications. The distribution of CA19-9 testing is relatively concentrated in the lower right quadrant of the central area; however, it exhibits a broad vertical distribution range (from −2 to +3), indicating considerable inter-study variability in the specificity dimension. The distribution of the combined CEA+CA19-9 testing lies between those of the individual biomarkers, demonstrating a relatively more concentrated distribution pattern (Figure 8).

**Figure 9:**
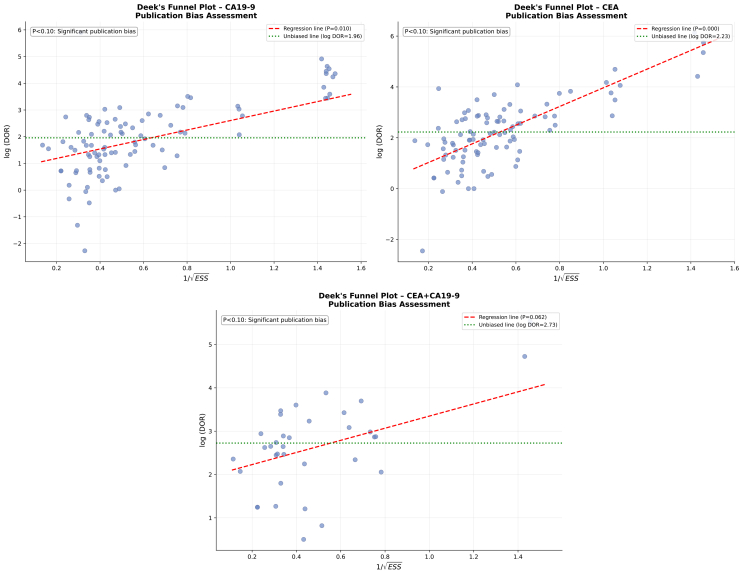
Deeks’ funnel plot

Deeks’ funnel plot analysis (Figure 9) provided statistical evidence for evaluating the risk of publication bias across the three testing strategies. The funnel plot for CA19-9 testing exhibited a generally symmetrical distribution of data points around the regression line, with the slope test P-value suggesting a relatively low risk of publication bias. The funnel plot for CEA testing demonstrates comparable distribution characteristics, with the majority of studies aligning along the regression line. However, a slight asymmetry is noted in the lower region, which may be linked to the selective publication of studies with smaller effect sizes.

### Sensitivity analysis

**Figure 10:**
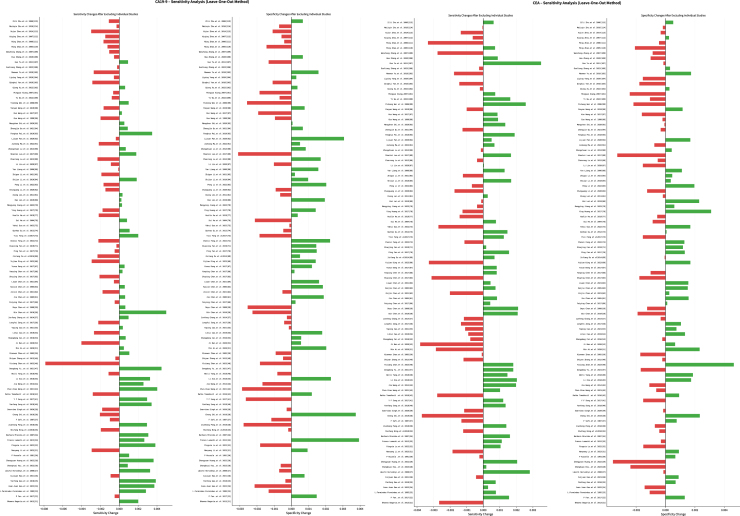
The sensitivity analysis of CA19-9 and CEA

The leave-one-out sensitivity analysis (Figure 10) conducted in this study demonstrated that the pooled sensitivity and specificity estimates for all three testing strategies exhibited considerable robustness, with the exclusion of individual studies showing no substantial impact on the overall conclusions. Notably, the combination of CEA and CA19-9 testing displayed the highest stability in the sensitivity analysis, exhibiting the narrowest range of variation in pooled estimates, thereby providing further support for the reliability of conclusions regarding the combined testing strategy.

The sensitivity analysis of CA19-9 testing demonstrated robust stability in results. Following the sequential removal of any individual study from the 95 included studies, the variation range of the pooled sensitivity estimates remained relatively narrow (approximately 0.01–0.02). The sensitivity analysis for CEA testing further confirmed the reliability of its diagnostic performance estimates. In the sensitivity dimension, the leave-one-out analysis demonstrated that the variation range of the pooled estimates was maintained within 0.008–0.018. In terms of specificity analysis, CEA testing exhibited superior stability characteristics, with the variation range of the pooled specificity estimates being only 0.003–0.012.

**Figure 11:**
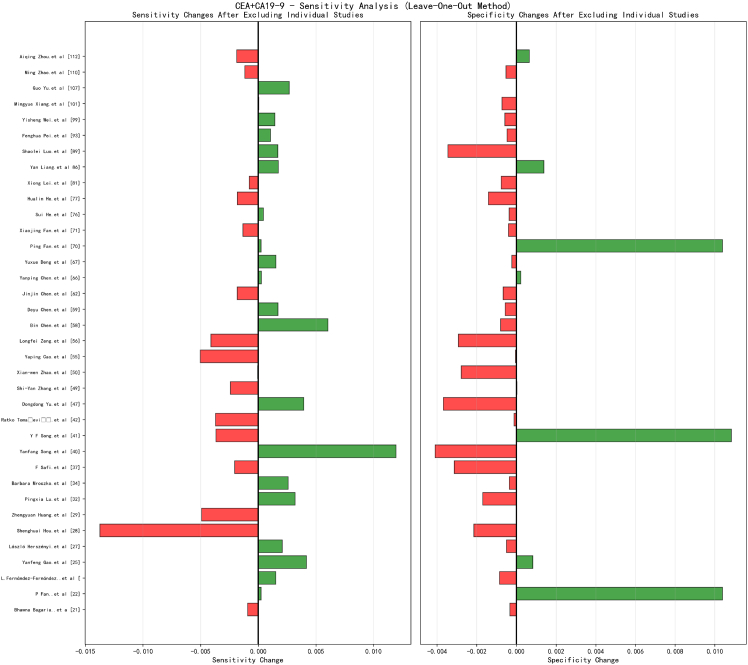
The sensitivity analysis results for the combined CEA+CA19-9 testing

The sensitivity analysis results for the combined CEA+CA19-9 testing (Figure 11) are particularly encouraging, demonstrating the most optimal robustness characteristics among the three testing strategies. Although the number of included studies is relatively limited (36 studies), the leave-one-out analysis revealed excellent stability in both pooled sensitivity and specificity estimates. The variation range for sensitivity estimates was only 0.005 to 0.015, while the variation range for specificity estimates was even more constrained within the narrow interval of 0.002–0.008.

## Discussion

This study systematically evaluates the performance of individual CEA, CA19-9, and their combined testing in the diagnosis of CRC through a meta-analysis. The results clearly demonstrate that the combined testing of CEA and CA19-9 represents the optimal diagnostic strategy among the three approaches, exhibiting significant advantages over individual biomarker tests across key metrics, including sensitivity, specificity, DOR, and AUC. These findings provide robust evidence-based support for clinical decision-making in the diagnosis of CRC.

Quantitative results indicate that the diagnostic advantage of combined testing possesses clear statistical and clinical significance. In terms of sensitivity, the pooled sensitivity of the combined test reached 0.585 (95% CI: 0.539–0.634), representing a 10.0% improvement over CEA alone (0.532) and a 32.7% increase over CA19-9 alone (0.441). This enhancement directly correlates with a reduction in the risk of missed diagnoses in clinical practice, suggesting that the combined test can identify a greater number of early-stage or occult CRC cases. Particularly for patients with earlier tumor stages or low expression of individual markers, the synergistic effect of multiple biomarkers aids in mitigating diagnostic delays caused by false negatives.

Regarding specificity, although the improvement of the combined test (0.877, 95% CI: 0.845–0.908) over individual tests is relatively modest (a 5.2% increase over CEA and 4.3% over CA19-9), its significance is substantial in large-scale population screening scenarios. For instance, in screening high-risk populations for CRC, each 1-percentage-point increase in specificity can prevent thousands of false-positive results, thereby avoiding unnecessary invasive procedures such as colonoscopy, reducing both the healthcare burden on patients and psychological anxiety, while conserving medical resources.

In terms of comprehensive performance metrics, the combined test achieved a DOR of 9.99, reflecting a 74.3% improvement over CEA (DOR 5.73) and a 138.4% increase over CA19-9 (DOR 4.19). This indicates a significantly enhanced capability to differentiate CRC patients from healthy controls. The SROC curve analysis revealed an AUC of 0.783 (95% CI: 0.760–0.807) for the combined test, which is substantially higher than that of CEA (0.662) and CA19-9 (0.725). Furthermore, the curve approaches the ideal diagnostic zone, maintaining a sensitivity greater than 0.6 even in high-specificity regions (>0.8), effectively mitigating the risk of over-diagnosis while ensuring comprehensive diagnostic coverage.

All three testing strategies employed in this study exhibited high heterogeneity, with I^2^ values of 91.2%, 91.4%, and 91.2% for CEA, CA19-9, and combined testing, respectively. This observation is consistent with the prevalent scenario in diagnostic biomarker research pertaining to CRC. The primary sources of heterogeneity can be categorized into three key aspects. First, variations in study design and patient selection: among the 95 individual testing studies and 36 combined testing studies included in this analysis, inconsistencies were noted in the inclusion criteria concerning patient tumor staging (early versus advanced), histological types (adenocarcinoma versus other subtypes), and comorbidities (such as inflammatory bowel disease and hepatobiliary diseases). Second, lack of standardization in detection methods and thresholds: variations were observed across studies regarding the detection platforms (e.g., chemiluminescence immunoassay versus enzyme-linked immunosorbent assay), reagent brands, and diagnostic thresholds (e.g., CEA cut-off values of 2.5 ng/mL versus 5 ng/mL). Third, heterogeneity in control group definitions: certain studies employed healthy populations as controls, while others included patients with benign bowel diseases (e.g., intestinal polyps or enteritis).

Despite the considerable heterogeneity, the large sample sizes (12,514 cases and 10,307 controls for CEA; 6,906 cases and 5,823 controls for combined testing) provided adequate statistical power for the random-effects model. Sensitivity analysis demonstrated that after sequentially excluding any individual study, the variation ranges of pooled sensitivity and specificity remained minimal (sensitivity variation range of 0.005–0.015 and specificity variation range of 0.002–0.008 for combined testing), indicating that the core conclusions are robust and not significantly influenced by individual outlier studies.

Likelihood ratio and Fagan’s nomogram analyses further validate the clinical utility of combined testing. The PLR of 4.73 for the combined test was significantly higher than that of CEA (3.21) and CA19-9 alone, indicating that its positive results possess greater confirmatory value for CRC. Conversely, its NLR of 0.47 was lower than that of the individual tests, demonstrating a higher reliability of negative results in ruling out the disease. In a moderate-risk population for CRC (pre-test probability approximately 10%), a positive combined test increased the post-test probability to 40–45%, thereby reaching the high suspicion threshold. Conversely, a negative result reduced the probability to 3–5%, approaching the effectively ruled out criterion.

In conclusion, this study confirms that the combined CEA+CA19-9 testing demonstrates significant advantages in the diagnosis of CRC and can be recommended as a preferred clinical strategy. However, the observed high heterogeneity and limitations of the studies indicate the necessity for further refinement through standardized, large-scale studies to better facilitate its application in precision diagnosis.

### Limitations of the study

Several limitations should be acknowledged when interpreting these findings. First, high heterogeneity was observed across all three testing strategies (I^2^ > 91%), which may be attributed to variations in patient populations, tumor staging, detection platforms, and diagnostic thresholds among included studies. Second, the number of studies available for combined CEA+CA19-9 analysis (*n* = 36) was substantially smaller than for individual markers (*n* = 95), which may limit the statistical power of subgroup analyses. Third, the majority of included studies were conducted in Asian populations, particularly Chinese cohorts, which may restrict the generalizability of findings to other ethnic groups. Fourth, differences in control group definitions (healthy individuals versus patients with benign bowel disease) across studies may have introduced spectrum bias. Fifth, although Deeks’ test suggested low publication bias, selective reporting of positive results cannot be entirely excluded. Future prospective, multicenter studies with standardized detection protocols are needed to validate these findings.

## Resource availability

### Lead contact

Further information and requests for resources and reagents should be directed to and will be fulfilled by the lead contact, Tao Zhang (zhangt760@zmu.edu.cn).

### Materials availability

This study did not generate new unique reagents.

### Data and code availability

• All data supporting the findings of this study are available within the paper and its supplemental information. The individual study-level 2 × 2 contingency table data extracted from included studies are provided in Tables 1 and 2.
• This study did not generate original code. Statistical analyses were performed using STATA 12 (StataCorp, College Station, TX, USA). No custom code was developed.
• Any additional information required to reanalyze the data reported in this paper is available from the lead contact upon request.

## Acknowledgments

This work was supported by the 10.13039/501100005329Guizhou Provincial Basic Research Program (Natural Science) (Qiankehe JichuZK2024yiban295), Zunyi City Science and Technology Innovation Team Construction Project (KCTD(2025)61), and the Program for High-Level Innovative “Thousand Level” Talents in Guizhou Province (Thousand Level).

This meta-analysis demonstrates an association between colorectal cancer and the co-testing of CEA and CA19-9. Although this meta-analysis is classified as a secondary study and the validity of its findings is contingent upon the quality of the original studies, it possesses certain limitations. Nevertheless, it offers a foundational reference for predicting precursors to colorectal cancer. Furthermore, it serves as a guide for research into the pathogenesis of CRC and provides evidence-based medical insights for the diagnosis and treatment of CRC.

## Author contributions

Conceptualization, L.Z., S.A., T.Z., and D.X.; methodology, L.Z.; formal analysis, L.Z.; writing – original draft, L.Z.; writing – review and editing, S.A., T.Z., and D.X.; visualization, Y.M. and G.L.; supervision, Y.Z., Y.M., and G.L.; project administration, S.A.; validation, Y.Z.

## Declaration of interests

The authors declare that they have no competing interests.

## Declaration of generative AI and AI-assisted technologies in the writing process

The authors confirm that the use of generative AI and AI-assisted technologies was explicitly excludedfrom all stages of this research and manuscript preparation. The ideas, analyses, and conclusions presented are entirely human-generated. The authors assume full responsibility for the originality and integrity of the work.

## STAR★Methods

### Key resources table

**Table:** 

REAGENT or RESOURCE	SOURCE	IDENTIFIER
**Deposited data**		

PubMed	https://pubmed.ncbi.nim.nih.gov/	N/A
EMBASE	https://www.embase.com/	N/A
Web of Science	https://www.webofscience.com/wos/author/search	N/A
CNKI	https://www.cnki.net/	N/A
Wanfang	https://www.wanfangdata.com.cn/	N/A

**Software and algorithms**		

Stata software Version 12.0	Downloaded STATA software	https://www.stata.com/products/
Review Manager 5.4	The Cochrane Collaboration	https://revman.cochrane.org/info
EndNote	Clarivate Analytics	http://www.myendnoteweb.com/

### Experimental model and study participant details

This study is a systematic review and meta-analysis of previously published studies. No new patient data were collected. Inclusion criteria required studies to enroll patients suspected of having colorectal cancer, with histopathological confirmation as the gold standard. Details of sex, age, and other participant characteristics were not uniformly reported across included studies; where available, these are summarized in Tables 1 and 2. The influence of sex and age on diagnostic performance could not be assessed due to insufficient reporting in primary studies, which represents a limitation acknowledged in the limitations of the study section.

### Method details

#### Search strategy and data sources

Computer searches were conducted to retrieve relevant literature published from the establishment of the China Knowledge Network (CNKI), Wanfang, China Medical Library (CMCC), SinoMed, Web of Science, and PubMed databases up to January 20, 2024. Search terms were identified using the Subject Search feature within SinoMed for Chinese MeSH equivalents and the MeSH search functionality in PubMed for English synonyms. Boolean operators (AND, OR) were used to connect keywords. Retrieved literature records were exported to EndNote software for duplicate removal.

#### Inclusion and exclusion criteria

Literature inclusion criteria: (1) study subjects comprised patients suspected of having colorectal cancer; (2) sufficient data were available to construct a 2×2 contingency table (TP, FP, TN, FN); (3) the study design was clearly described, including inclusion/exclusion criteria and baseline characteristics; (4) colonoscopic biopsy histopathology was explicitly defined as the gold standard for CRC diagnosis; (5) threshold values for relevant tumor markers were established within the study. Literature exclusion criteria: (1) studies with follow-up periods exceeding one year; (2) studies with sample sizes smaller than 30; (3) studies that did not clearly specify detection methods; (4) studies that did not establish threshold values for tumor markers; (5) studies lacking a clear histopathological diagnosis as the reference standard; (6) studies reporting only sensitivity/specificity without raw 2×2 data.

#### Data extraction

A standardized data extraction form was used to collect the following information: (1) basic research information: authors, year of publication, study design; (2) basic characteristics of tumor markers: method of detection, threshold value; (3) results: TP, FP, TN, and FN values, derived directly from the original literature or calculated from reported sensitivity and specificity. Two independent reviewers screened the literature and extracted data. Discrepancies were resolved by consultation with a third reviewer.

#### Quality assessment

The methodological quality of included studies was assessed using the QUADAS-2 tool, which evaluates risk of bias and applicability concerns across four domains: patient selection, index test, reference standard, and flow and timing. Two reviewers independently assessed each study, and disagreements were resolved through consensus.

### Quantification and statistical analysis

Pooled sensitivity and specificity for the three testing strategies were calculated using a bivariate random-effects model implemented in STATA 12 (Stata Corp, College Station, TX, USA). This model accounts for potential correlations between sensitivity and specificity, avoiding biases inherent in single-indicator analyses. Heterogeneity was quantified using the I^2^ statistic (I^2^ > 75% indicating high heterogeneity) and visualized using bivariate box plots. Summary receiver operating characteristic (SROC) curves were constructed using the HSROC model, with pooled AUC and 95% CI calculated and reported for each testing strategy. Clinical utility was assessed using Fagan’s nomogram and likelihood ratio scatter plots. Publication bias was assessed using Deeks’ test in conjunction with funnel plots (P < 0.05 indicating significant publication bias). Sensitivity analysis was conducted using the leave-one-out method. All data are presented as pooled estimates with 95% confidence intervals.

### Additional resources

PROSPERO registration number: CRD420251101973.

## Supplemental information

Document S1. Data S1 and S2

Data S3. PRISMA 2020 checklist: where to report each item in a systematic review and relevant descriptions
