# Supplementary material for: Diagnostic value of combined serum CEA and CA19-9 in colorectal cancer A meta-analysis
Source: iScience. 2026 May 4;29(5):115639. doi: 10.1016/j.isci.2026.115639 (PMC13157001; doi:10.1016/j.isci.2026.115639)
Supplement: Document S1. Data S1 and S2 [file mmc1.pdf]

iScience, Volume *29*

Supplemental information

Diagnostic value of combined serum CEA and CA19-9 in colorectal cancer A meta-analysis

Liushaoqiu Zhou, Songlin An, Yuanzhong Zhou, Yonghong Ma, Guanyu Li, Tao Zhang, and Daimin Xiao

Data S1：Strategy for Colorectal Cancer-Related Tumor Marker Research

1、Combined plasma CEA and associated protein levels have higher sensitivity than plasma CEA alone in colorectal cancer patients-Wang W(2014)-No data available(in China).

2、Role of serum Metadherin mRNA expression in the diagnosis and prediction of survival in patients with colorectal cancer-Muhammad Tarek Abdel Ghafar(2020)-No data available.

3、Serum Tumor Marker Use in Patients With Advanced Solid Tumors-Melissa K. Accordino (2015)-No data available.

4、Use of Tumor Markers in Gastrointestinal Cancers: Surgeon Perceptions and Cost-Benefit Trade-Off Analysis-Amish Acharya (2016)-Data incompleteness.

5、Assessment of Serum Levels of the Adipocytokine Chemerin in Colorectal Cancer Patients-J Med Biochem(2018)-Other tumor markers described in the literature.

6、Sensitivity of monoclonal antibodies to carcinoembryonic antigen, tissue polypeptide antigen, alpha-fetoprotein, carbohydrate antigen 50, and carbohydrate antigen 19-9 in the diagnosis of colorectal adenocarcinoma-J A Alvarez(1995)-Data incompleteness.

7、Determination and significance of a new carbohydrate antigen CA19-9 in digestive system cancers-Y Arakawa(1985)-Exclusion criteria met; sample size less than 30.

8、Clinical value of a diagnostic score for colon cancer based on serum CEA, CA19-9, cytokeratin-1 and mucin-1-A M Attallah(2018)-Other tumor markers described in the literature.

9、Normal and modified urinary nucleosides represent novel biomarkers for colorectal cancer diagnosis and surgery monitoring.-Bo Feng (2005)-Other tumor markers described in the literature.

10、Role of CEA, TPA, and Ca 19-9 in the early detection of localized and diffuse recurrent rectal cancer-P Barillari（1992）-The content of this literature is not relevant to the diagnosis of colorectal cancer.

11、Value of CT, FDG PET-CT and serum tumor markers in staging recurrent colorectal cancer-Meltem Caglar (2015)-The content of this literature is not relevant to the diagnosis of colorectal cancer.

12、Serum CA724 has no diagnostic value for gastrointestinal tumors-Huiru Cao(2023)-The content of this literature is not relevant to the diagnosis of colorectal cancer.

13、Estimating the probability of cancer with several tumor markers in patients with colorectal disease-Monika Carpelan-Holmström (2004)-No data available.

14、CEA, CA 242, CA 19-9, CA 72-4 and hCGbeta in the diagnosis of recurrent colorectal cancer-Monika Carpelan-Holmström (2004)-The content of this literature is not relevant to the diagnosis of colorectal cancer.

15、Serum macrophage inhibitory cytokine-1 serves as a novel diagnostic biomarker of early-stage colorectal cancer-Chunyang Dai (2021)-Other tumor markers described in the literature.

16、Clinical Performance of CEA, CA19-9, CA15-3, CA125 and AFP in Gastrointestinal Cancer Using LOCI™-based Assays-Ramona C Dolscheid-Pommerich (2017)-Exclusion criteria met; sample size less than 30.

17、Identification of Serum Periostin as a Potential Diagnostic and Prognostic Marker for Colorectal Cancer-Dong Dong (2018)-Exclusion criteria met; sample size less than 30.

18、Serum levels of matrix metalloproteinase-2 and -9 and conventional tumor markers (CEA and CA 19-9) in patients with colorectal and gastric cancers-Marwan Emara (2009)-Exclusion criteria met; sample size less than 30.
19、Circulating Cell Free DNA Integrity Index as a Biomarker for Response to Chemotherapy in Patients with Metastatic Colorectal Carcinoma-Nancy Samir Eskander (2022)-Other tumor markers described in the literature.
20、Normal and modified urinary nucleosides represent novel biomarkers for colorectal cancer diagnosis and surgery monitoring-Bo Feng (2005)-Other tumor markers described in the literature.
21、[Application of urinary nucleosides in the diagnosis and surgical monitoring of colorectal cancer]-Bo Feng (2005)-Data incompleteness.
22、Prognostic value of CA 19.9 levels in colorectal cancer-X Filella (1992)-Exclusion criteria met; sample size less than 30.
23、Use of CA 19-9 in the early detection of recurrences in colorectal cancer: comparison with CEA-X Filella (1994)-The content of this literature is not relevant to the diagnosis of colorectal cancer.
24、Diagnostic performances of leucine-rich α-2-glycoprotein 1 and stem cell factor for diagnosis and follow-up of colorectal cancer-Manar S Fouda (2021)-Exclusion criteria met; sample size less than 30.
25、The diagnostic value of CA 27-29, CA 15-3, mucin-like carcinoma antigen, carcinoembryonic antigen and CA 19-9 in breast and gastrointestinal malignancies-P S Frenette (1994)-Data incompleteness.
26、Follow-up of colorectal cancer resected for cure. An experience with CEA, TPA, Ca 19-9 analysis and second-look surgery-C Fucini (1987)-The content of this literature is not relevant to the diagnosis of colorectal cancer.
27、A new approach to tumor marker assessment by perioperative determination in breast and colorectal cancer-M Gion (1993)-Data incompleteness.
28、Do pre-treatment serum levels of CEA and Ca19-9 predict prognosis in patients with colorectal cancer? - Hao Huang (2019)-The content of this literature is not relevant to the diagnosis of colorectal cancer.
29、TM9SF4 expression in tumor tissues: a novel diagnostic biomarker for gastrointestinal tumors-Paolo Guazzi (2020)-Other tumor markers described in the literature.
30、Inflammation-based markers can predict the prognosis of geriatric patients with metastatic colorectal cancer receiving first-line chemotherapy-Guifang Guo (2019)-The content of this literature is not relevant to the diagnosis of colorectal cancer.
31、Tumor marker CA 125 in patients with digestive tract malignancies-C Haglund (1991)-Data incompleteness.
32、Tumor markers CA 19-9 and CA 50 in digestive tract malignancies-C Haglund (1992)-Other tumor markers described in the literature.
33、Cellular fibronectin concentration in the plasma of patients with malignant and benign diseases: a comparison with CA 19-9 and CEA-C. Haglund (1997)-Other tumor markers described in the literature.
34、Tumor markers and screening for gastrointestinal cancer: a follow up study in Finland-M Hakama (1994)-Data incompleteness.
35、Accuracy of monitoring serum carcinoembryonic antigen levels in postoperative stage III

colorectal cancer patients is limited to only the first postoperative year-MASAYASU HARA (2011)-The content of this literature is not relevant to the diagnosis of colorectal cancer.
36、Clinical value of circulating tumor cells and hematological parameters in 617 Chinese patients with colorectal cancer: retrospective analysis-Yuhao He (2023)-Data incompleteness.
37、Comparison of CA 72-4 with CA 19-9 and carcinoembryonic antigen in the serodiagnostics of gastrointestinal malignancies-G Heptner (1989)-Other tumor markers described in the literature.
38、[Comparison of the tumor markers CEA and CA 19-9 in colorectal diagnosis]-H Putzki (1987)-Data incompleteness.
39、N (1), N (12)-Diacetylspermine as a sensitive and specific novel marker for early- and late-stage colorectal and breast cancers-Kyoko Hiramatsu (2005)-Other tumor markers described in the literature.
40、Cytokeratin serum biomarkers in patients with colorectal cancer-S Holdenrieder (2012)-Exclusion criteria met; sample size less than 30.
41、Clinical Value of Combined Detection of Serum sTim-3 and CEA or CA19-9 for Postoperative Recurrence of Colorectal Cancer Diagnosis-Jianfeng Hong (2023)-The content of this literature is not relevant to the diagnosis of colorectal cancer.
42、Meprin α combined with CEA and CA19-9 improves prognostic prediction for surgically treated colorectal cancer patients-Hongfa Hou (2017)-The content of this literature is not relevant to the diagnosis of colorectal cancer.
43、Diagnostic value of combined detection of multiple tumor markers and blood lipid indexes in colorectal cancer and its prediction on adverse reactions of chemotherapy-Mingzhe Jiang (2021)-Data incompleteness.
44、First alert for recurrence during follow-up after potentially curative resection for colorectal carcinoma: CA 19-9 should be included in surveillance programs-Yutaka J Kawamura (2010)-The content of this literature is not relevant to the diagnosis of colorectal cancer.
45、Early Screening of Colorectal Precancerous Lesions Based on Combined Measurement of Multiple Serum Tumor Markers Using Artificial Neural Network Analysis-Xing Ke (2023)-The content of this literature is not relevant to the diagnosis of colorectal cancer.
46、Usefulness of carcinoembryonic antigen for monitoring tumor progression during palliative chemotherapy in metastatic colorectal cancer-Gangmi Kim (2013)-Exclusion criteria met; sample size less than 30.
47、Tissue polypeptide-specific antigen (TPS) in monitoring palliative treatment response of patients with gastrointestinal tumors-G Kornek (1995)-Data incompleteness.
48、Elevated CA19-9 as the most significant prognostic factor in advanced colorectal carcinoma-Lu-Ning Zhang (2015)-The content of this literature is not relevant to the diagnosis of colorectal cancer.
49、Clinical significance of serum anti-p53 antibody expression following curative surgery for colorectal cancer-Kensuke Kumamoto (2017)-Other tumor markers described in the literature.
50、A comparison of tumor M2-PK with carcinoembryonic antigen and CA19-9 in patients undergoing liver resection for colorectal metastases-Yogesh Kumar (2008)-The content of this literature is not relevant to the diagnosis of colorectal cancer.
51、Clinical Value of Serum p53 Antibody in the Diagnosis and Prognosis of Colorectal Cancer-Masaki Kunizaki (2016)-Other tumor markers described in the literature.
52、Comparison of CA 19-9 and carcinoembryonic antigen (CEA) levels in the serum of patients

with colorectal diseases-P Kuusela (1984)-Data incompleteness.
53、Lipid peroxidation as additional marker in patients with colorectal cancer. Results of a preliminary study-H Lauschke (2002)-Data incompleteness.
54、Circulating methylated septin 9 nucleic Acid in the plasma of patients with gastrointestinal cancer in the stomach and colon-Hye Seung Lee (2013)-Other tumor markers described in the literature.
55、Clinical Usefulness of Serum CYFRA 21-1 in Patients with Colorectal Cancer-Jai Hyuen Lee (2013)-Data incompleteness.
56、Chemiluminescent dual-enzyme immunoassays capable of simultaneously quantifying carbohydrate antigen 19-9 and carcinoma embryonic antigen in a sample-Yujung Lee (2019)-The content of this literature is not relevant to the diagnosis of colorectal cancer.
57、Methylated Septin9 identified patients with colorectal carcinoma and showed higher sensitivity than conventional biomarkers in detecting tumor-Sabine Leerhoff (2023)-Other tumor markers described in the literature.
58、Revisiting ab initio carcinoembryonic antigen and CA19-9 tumor markers in colorectal carcinoma in association with anatomotopographic location and staging of disease-Milan Lerch (2023)-The content of this literature is not relevant to the diagnosis of colorectal cancer.
59、Prediction models of colorectal cancer prognosis incorporating perioperative longitudinal serum tumor markers: a retrospective longitudinal cohort study-Chunxia Li (2023)-The content of this literature is not relevant to the diagnosis of colorectal cancer.
60、The correlation between pre-operative serum tumor markers and lymph node metastasis in gastric cancer patients undergoing curative treatment-Fangxuan Li (2013)-The content of this literature is not relevant to the diagnosis of colorectal cancer.
61、Early detection of colorectal cancer based on circular DNA and common clinical detection indicators-Jian Li (2022)-Data incompleteness.
62、Hsa_circ_0124554 may serve as a biomarker for the diagnosis of colorectal cancer: An observational study-Kexin Li (2023)-Other tumor markers described in the literature.
63、Study on specificity of colon carcinoma-associated serum markers and establishment of SVM prediction model-Lu Li (2017)-The content of this literature is not relevant to the diagnosis of colorectal cancer.
64、Serum Chemokine CXCL7 as a Diagnostic Biomarker for Colorectal Cancer-Longhai Li (2021)-Other tumor markers described in the literature.
65、Nomograms for predicting the prognostic value of serological tumor biomarkers in colorectal cancer patients after radical resection-Qingguo Li (2017)-The content of this literature is not relevant to the diagnosis of colorectal cancer.
66、Combination of serum lipids and cancer antigens as a novel marker for colon cancer diagnosis-Tong Li (2018)-Data incompleteness.
67、Plasma circular RNA panel acts as a novel diagnostic biomarker for colorectal cancer-Jie Lin (2019)-Other tumor markers described in the literature.
68、Serum microRNA signatures and metabolomics have high diagnostic value in colorectal cancer using two novel methods-Hai-Ning Liu (2018)-Other tumor markers described in the literature.
69、Diagnostic and economic value of carcinoembryonic antigen, carbohydrate antigen 19-9, and carbohydrate antigen 72-4 in gastrointestinal cancers-Hai-Ning Liu (2023)-Data incompleteness.

70、CA 72-4 compared with CEA and CA 19-9 as a marker of some gastrointestinal malignancies-J B Lopez (1999)-Exclusion criteria met; sample size less than 30.
71、Tumor markers, liver function tests and symptoms in 115 patients with isolated colorectal liver metastases-M Lorenz (1989)-Data incompleteness.
72、Serum HCG beta, CA 72-4 and CEA are independent prognostic factors in colorectal cancer-Johanna Louhimo (2022)-Data incompleteness.
73、Methylated Septin9 has moderate diagnostic value in colorectal cancer detection in Chinese population: a multicenter study-Dong-Cheng Lu (2022)-Other tumor markers described in the literature.
74、Optimal Strategy for Colorectal Cancer Patients' Diagnosis Based on Circulating Tumor Cells and Circulating Tumor Endothelial Cells by Subtraction Enrichment and Immunostaining-Fluorescence in Situ Hybridization Combining with CEA and CA19-9-Shimu Luo (2021)-Exclusion criteria met; sample size less than 30.
75、Gamma-amino-butyric acid immunoreactivity in intramucosal colonic tumors-Kentaro Maemura (2003)-The content of this literature is not relevant to the diagnosis of colorectal cancer.
76、Clinical utility of serum tumor markers in the diagnosis of malignant intestinal occlusion. A prospective observational study-Daniele Marrelli (2011)-Exclusion criteria met; sample size less than 30.
77、Does serum CA19-9 play a practical role in the management of patients with colorectal cancer? - Shunji Morita (2004)-The content of this literature is not relevant to the diagnosis of colorectal cancer.
78、Diagnostic usefulness of serum interleukin 6 (IL-6) and C-reactive protein (CRP) in the differentiation between pancreatic cancer and chronic pancreatitis-Barbara Mroczko (2010)-The content of this literature is not relevant to the diagnosis of colorectal cancer.
79、The diagnostic value of matrix metalloproteinase 9 (MMP-9) and tissue inhibitor of matrix metalloproteinases 1 (TIMP-1) determination in the sera of colorectal adenoma and cancer patients-Barbara Mroczko (2010)-Other tumor markers described in the literature.
80、The diagnostic value of G-CSF measurement in the sera of colorectal cancer and adenoma patients-Barbara Mroczko (2006)-Data incompleteness.
81、Granulocyte-colony stimulating factor (G-CSF) and macrophagecolony stimulating factor (M-CSF) in colorectal cancer patients-Barbara Mroczko (2002)-Other tumor markers described in the literature.
82、Hematopoietic growth factors in colorectal cancer patients-Barbara Mroczko (2003)-Other tumor markers described in the literature.
83、Stem cell factor (SCF) and interleukin 3 (IL-3) in the sera of patients with colorectal cancer-Barbara Mroczko (2005)-Other tumor markers described in the literature.
84、Prognostic values of serum CA19-9 and CEA levels for colorectal cancer-T Nakayama (1997)-The content of this literature is not relevant to the diagnosis of colorectal cancer.
85、Intensive risk-adjusted follow-up with the CEA, TPA, CA19.9, and CA72.4 tumor marker panel and abdominal ultrasonography to diagnose operable colorectal cancer recurrences: effect on survival-Andrea Nicolini (2010)-The content of this literature is not relevant to the diagnosis of colorectal cancer.
86、Increase in both CEA and CA19-9 in sera is an independent prognostic indicator in colorectal carcinoma-Tadahiro Nozoe (2006)-No data available.

87、Serum CA19-9 is significantly upregulated up to 2 years before diagnosis with pancreatic cancer: implications for early disease detection-Darragh P O'Brien (2015)-The content of this literature is not relevant to the diagnosis of colorectal cancer.

88、Prognostic Value of Preoperative Carcinoembryonic Antigen and Carbohydrate Antigen 19-9 Levels for Adjuvant Chemotherapy in Stage II Colorectal Cancer: A Nationwide Multicenter Retrospective Study-Suguru Ogata (2022)-The content of this literature is not relevant to the diagnosis of colorectal cancer.

89、The role of periodic serum CA19-9 test in surveillance after colorectal cancer surgery-Ryosuke Okamura (2017)-The content of this literature is not relevant to the diagnosis of colorectal cancer.

90、Serum levels of CA 125 in patients with gastrointestinal cancers-Y T Omar (1989)-The content of this literature is not relevant to the diagnosis of colorectal cancer.

91、Clinical Significance of Preoperative and Postoperative Serum CEA and Carbohydrate Antigen 19-9 Levels in Patients Undergoing Curative Resection of Locally Recurrent Rectal Cancer-Masakatsu Paku (2022)-The content of this literature is not relevant to the diagnosis of colorectal cancer.

92、Clinical significance and prognostic value of serum sHER-2/neu levels in patients with solid tumors-C Papila-(2009)-Other tumor markers described in the literature.

93、Postoperative CEA is a better prognostic marker than CA19-9, hCGβ or TATI after resection of colorectal liver metastases-Reetta Peltonen (2018)-The content of this literature is not relevant to the diagnosis of colorectal cancer.

94、Multiplexed cytokine profiling of serum for detection of colorectal cancer-Zhang Pengjun (2013)-Other tumor markers described in the literature.

95、Serum TEM5 and TEM7 concentrations correlate with clinicopathologic features and poor prognosis of colorectal cancer patients-Łukasz Pietrzyk (2019)-The content of this literature is not relevant to the diagnosis of colorectal cancer.

96、Serum tumor markers in colorectal cancer staging, grading, and follow-up-M Plebani (1996)-Data incompleteness.

97、Tumor markers carcinoembryonic antigen, tissue polypeptide antigen, and carbohydrate antigen 19/9 in liver diseases-H Putzki (1988)-The content of this literature is not relevant to the diagnosis of colorectal cancer.

98、Cardiac, Hepatic and Renal Dysfunction and IL-18 Polymorphism in Breast, Colorectal, and Prostate Cancer Patients-Govand Qader (2021)-Data incompleteness.

99、Alu-based cell-free DNA: a potential complementary biomarker for diagnosis of colorectal cancer-Jing Qi (2013)-Other tumor markers described in the literature.

100、LncRNA HCP5: A Potential Biomarker for Diagnosing Gastric Cancer-Shiyi Qin(2021)-The content of this literature is not relevant to the diagnosis of colorectal cancer.

101、Colorectal Cancer (CRC): Investigating the Expression of the Suppressor of Fused (SuFu) Gene and Its Relationship with Several Inflammatory Blood-Based Biomarkers-Tahseen Bilal Rather (2023)-The content of this literature is not relevant to the diagnosis of colorectal cancer.

102、Tumor markers in colorectal cancer-P J Roberts-(1988)-Data incompleteness.

103、Comparison of CA 72-4, CA 19-9 and CEA in the diagnosis and monitoring of gastric cancer-F Safi (1995)-The content of this literature is not relevant to the diagnosis of colorectal cancer.

104、A clinical evaluation of carbohydrate antigen 19-9 and carcinoembryonic antigen in patients with pancreatic carcinoma-K Satake (1985)-The content of this literature is not relevant to the diagnosis of colorectal cancer.
105、Limited usefulness of serum carcinoembryonic antigen and carbohydrate antigen 19-9 levels for gastrointestinal and whole-body cancer screening-Masau Sekiguchi (2020)-Data incompleteness.
106、[Study on clinicopathological correlations between metabolic syndrome and colorectal carcinoma]-Zhan-Long Shen (2008)-No data available.
107、Significance of CEA and CA19-9 combination as a prognostic indicator and for recurrence monitoring in patients with stage II colorectal cancer-Masatsune Shibutani (2014)-Data incompleteness.
108、Serum leucine-rich alpha-2-glycoprotein-1 with fucosylated triantennary N-glycan: a novel colorectal cancer marker-Eiji Shinozaki (2018)-Other tumor markers described in the literature.
109、Predictive value of carcinoembryonic antigen and carbohydrate antigen 19-9 related to downstaging to stage 0-I after neoadjuvant chemoradiotherapy in locally advanced rectal cancer-Jianyuan Song (2018)-No data available.
110、[Clinical significance of the circulating tumor-associated antigen CA 19-9 in cancers of the digestive tract]-H J Staab (1984)-The content of this literature is not relevant to the diagnosis of colorectal cancer.
111、Comparison of the sensitivity and specificity of the CA19-9 and carcinoembryonic antigen assays in detecting cancer of the pancreas-W M Steinberg (1986)-The content of this literature is not relevant to the diagnosis of colorectal cancer.
112、[Clinical significance of the circulating tumor-associated antigen CA 19-9 in cancers of the digestive tract]-H J Staab (1984)-The content of this literature is not relevant to the diagnosis of colorectal cancer.
113、Comparison of the sensitivity and specificity of the CA19-9 and carcinoembryonic antigen assays in detecting cancer of the pancreas-W M Steinberg (1986)-The content of this literature is not relevant to the diagnosis of colorectal cancer.
114、Can preoperative CEA and CA19-9 serum concentrations suggest metastatic disease in colorectal cancer patients? - Milica Stojkovic Lalosevic (2017)-Data incompleteness.
115、The role of (m)SEPT9 in screening, diagnosis, and recurrence monitoring of colorectal cancer-Jie Sun (2019)-Other tumor markers described in the literature.
116、Serum haptoglobin as a novel molecular biomarker predicting colorectal cancer hepatic metastasis-Lichao Sun (2016)-The content of this literature is not relevant to the diagnosis of colorectal cancer.
117、Increase in both CEA and CA19-9 in sera is an independent prognostic indicator in colorectal carcinoma. %J Journal of Surgical Oncology %J-Tadahiro Nozoe (2006)-No data available.
118、Clinical significance of tumor markers in patients with type 2 diabetes: a retrospective observational study-Maho Taguchi (2022)-The content of this literature is not relevant to the diagnosis of colorectal cancer.
119、Relationship between serum ELAM-1 and metastasis among patients with colon cancer
-Y Takahashi (1998)-The content of this literature is not relevant to the diagnosis of colorectal cancer.
120、The utility of tumor marker combination, including serum P53 antibody, in colorectal cancer

treatment-Ryuma Tokunaga(2017)-Data incompleteness.

121、Carcinoembryonic antigen (CEA), alpha-fetoprotein, CA 19.9 and CA 125 in advanced colorectal cancer (ACC)-N Tsavaris (1993)-Data incompleteness.

122、Evaluating the utility of N1,N12-diacetylspermine and N1,N8-diacetylspermidine in urine as tumor markers for breast and colorectal cancers-Yoshifumi Umemori (2010)-Other tumor markers described in the literature.

123、Comparison of four serum tumor markers in the diagnosis of colorectal carcinoma-Y T van der Schouw(1992)-Data incompleteness.

124、Design, expression and evaluation of novel chimeric protein constructed from colorectal tumor-associated antigen-Ziba Veisi Malekshahi (2018)-No data available.

125、The application of current diagnostic protocols of patients with colon cancer in preparation for therapy-Zora Vukobrat-Bijedic (2010)-Data incompleteness.

126、Combined detection of preoperative serum CEA, CA19-9 and CA242 improve prognostic prediction of surgically treated colorectal cancer patients-Jingtao Wang(2015)-Data incompleteness.

127、[Clinical significance of plasma fibrinogen level in patients with colorectal cancer]-Qiong Wang(2005)-Other tumor markers described in the literature.

128、Leukocyte immunoglobulin-like receptor B2 overexpression as a promising therapeutic target and noninvasive screening biomarker for colorectal cancer-Qian-Qian Wang(2023)-Other tumor markers described in the literature.

129、Evaluating the significance of expression of CEA mRNA and levels of CEA and its related proteins in colorectal cancer patients-Wenyuan Wang(2014)-Data incompleteness.

130、Evaluation of the clinical application of multiple tumor marker protein chip in the diagnostic of lung cancer-Xiaochuan Wang(2018)-Data incompleteness.

131、Efficiency of the combination of ¹⁸F-FDG PET/CT, CEA, and CA199 in detection of colorectal cancer and monitoring postoperative tumor metastasis-Ruohua Chen(2022)-Other tumor markers described in the literature.

132、Detection and Clinical Significance of DLC1 Gene Methylation in Serum DNA from Colorectal Cancer Patients-Ping-Ping Wu(2011)-Other tumor markers described in the literature.

133、New Assay System Elecsys Anti-p53 to Detect Serum Anti-p53 Antibodies in Esophageal Cancer Patients and Colorectal Cancer Patients: Multi-institutional Study-Satoshi Yajima (2021)-Other tumor markers described in the literature.

134、Clinical significance of CEA and CA19-9 in postoperative follow-up of colorectal cancer-Tomomi Yakabe (2010)-Data incompleteness.

135、Carbohydrate antigen 242 highly consists with carbohydrate antigen 19-9 in diagnosis and prognosis of colorectal cancer: study on 185 cases-Xue-Qin Yang (2012)-Other tumor markers described in the literature.

136、Identification of Cystatin SN as a novel tumor marker for colorectal cancer-Kyoko Yoneda(2009)-Other tumor markers described in the literature.

137、[Evaluation of combined CA-19-9 and CEA assay in monitoring recurrences and metastases of colorectal cancer]-B M Yu (1992)-The content of this literature is not relevant to the diagnosis of colorectal cancer.

138、Significant diagnostic value of circulating tumor cells in colorectal cancer-Haijiao Yu(2020)-Other tumor markers described in the literature.

139、The clinical significance of preoperative serum levels of carbohydrate antigen 19-9 in colorectal cancer-Hyeon Yu(2013)-No data available.
140、Tumor biomarkers: help or mislead in the diagnosis of xanthogranulomatous cholecystitis? -analysis of serum CA 19-9, carcinoembryonic antigen, and CA 12-5-Hong Yu (2013)-The content of this literature is not relevant to the diagnosis of colorectal cancer.
141、Serum circular RNA hsa_circ_0000702 as a novel biomarker for diagnosis of gastric cancer-Wentao Yuan(2023)-The content of this literature is not relevant to the diagnosis of colorectal cancer.
142、Eotaxins and Their Receptor as Biomarkers of Colorectal Cancer-Monika Zajkowska(2021)-The content of this literature is not relevant to the diagnosis of colorectal cancer.
143、Models of logistic regression analysis, support vector machine, and back-propagation neural network based on serum tumor markers in colorectal cancer diagnosis-B Zhang(2016)-Other tumor markers described in the literature.
144、Ultrasound/CT combined with serum CEA/CA19.9 in the diagnosis and prognosis of rectal cancer-Beibei Zhang(2018)-Data incompleteness.
145、Combining Serum DNA Methylation Biomarkers and Protein Tumor Markers Improved Clinical Sensitivity for Early Detection of Colorectal Cancer-Guoying Zhang(2021)-Data incompleteness.
146、Improved diagnostic value by combining plasma PON1 level with tumor biomarkers in Colorectal Cancer patients-Jingdan Zhang(2020)-Exclusion criteria met; no threshold values for tumor markers given.
147、Accuracy evaluation of combining gastroscopy, multi-slice spiral CT, Her-2, and tumor markers in gastric cancer staging diagnosis-Songbo Zhao(2022)-The content of this literature is not relevant to the diagnosis of colorectal cancer.
148、The prognostic value of preoperative serum levels of CEA, CA19-9 and CA72-4 in patients with colorectal cancer-C X Zheng(2001)-Data incompleteness.
149、Prognostic significance of carcinoembryonic antigen combined with carbohydrate antigen 19-9 following neoadjuvant chemoradiotherapy in patients with locally advanced rectal cancer-Zhifang Zheng(2021)-Data incompleteness.
150、Serum GROβ: a potential tumor-associated biomarker for colorectal cancer-Zhaoxu Zheng (2015)-Data incompleteness.
151、Association of serum levels of CEA, CA199, CA125, CYFRA21-1 and CA72-4 and disease characteristics in colorectal cancer-Wa Zhong(2015)-Data incompleteness.
152、Combination of preoperative CEA and CACA19-9 improves prediction outcomes in patients with resectable pancreatic adenocarcinoma: results from a large follow-up cohort-Guofeng Zhou (2017-The content of this literature is not relevant to the diagnosis of colorectal cancer.
153、Clinical value of combining serum tumor marker detection with fecal occult blood testing in diagnosing colorectal cancer-H Zhou(2022)-Data incompleteness.
154、The Significance of Serum S100A9 and TNC Levels as Biomarkers in Colorectal Cancer-Minze Zhou(2019)-Exclusion criteria met; no threshold values for tumor markers given.
155、Diagnostic value of an enhanced MRI combined with serum CEA, CA19-9, CA125 and CA72-4 in the liver metastasis of colorectal cancer-Hua-Qiang Zhu(2022)-The content of this literature is not relevant to the diagnosis of colorectal cancer.
156、Correction: Diagnostic value of an enhanced MRI combined with serum CEA, CA19-9,

CA125 and CA72-4 in the liver metastasis of colorectal cancer-Hua-Qiang Zhu (2023)-Data incompleteness.
157、Simultaneous detection of four biomarkers with one sensing surface based on redox probe tagging strategy-Qiang Zhu(2013)-The content of this literature is not relevant to the diagnosis of colorectal cancer.
158、The value of CA19-9, AFP and CEA co-testing in the diagnosis of malignant tumors of the digestive tract-Bao Cuixia(2006)-No data available(in China).
159、The value of serum CEA, CA199, CA724 combined with mucosal telomerase assay in the diagnosis and prognostic evaluation of colorectal cancer-Bi Chao(2008)-Data incompleteness(in China).
160、Diagnostic value of serum CEA, CA50, CA19-9 and SA combination test for rectal cancer -Bu Jing Yun(2012)-Data incompleteness(in China).
161、The value of combined CA19-9 and CEA testing in colorectal cancer-Huifang Cao(1993)-Data incompleteness(in China).
162、Study on the value of serum tumor marker combination test applied to the clinical diagnosis of colorectal cancer- Xianbo Cao (2018)-Data incompleteness(in China).
163、Expression and clinical significance of plasma circHIPK3 in colorectal cancer patients-Yu Cao(2021)-Data incompleteness(in China).
164、Clinical study of combined and dynamic serum tumor marker assays for disease monitoring in colorectal cancer patients-Yujuan Cao(2018)-Data incompleteness(in China).
165、Analysis of the diagnostic value of combined stool and tumor marker testing in colorectal cancer-Zhihua Cao(2017)--Data incompleteness(in China).
166、Logistic regression and PLS-DA model to assess the diagnostic value of tumor markers in colorectal cancer -Longfei Zeng(2017)-Data duplication(in China).
167、Diagnostic value of combined detection of tumor markers in colorectal cancer-Qinghai Zeng(2015)-Exclusion criteria met; no threshold values for tumor markers given (in China).
168、Relationship between serum CEA and CA19-9 levels and liver metastases in patients with colorectal cancer-Yan Zeng(2019)-Data incompleteness(in China).
169、The value of serum CRP and tumor marker testing in the diagnosis of gastrointestinal malignancies-Ahong Chen(2022)-The content of this literature is not relevant to the diagnosis of colorectal cancer(in China).
170、Adjuvant diagnostic value of abnormal prothrombin in combination with CA50, CA19-9 and CEA in colorectal cancer liver metastases-Fangbing Chen(2019)-The content of this literature is not relevant to the diagnosis of colorectal cancer(in China).
171、Evidence on the value of combined stool and tumor marker testing in the diagnosis and management of colorectal cancer-Huajian Chen(2020)-Data incompleteness(in China).
172、Exploring the clinical application of protein microarray technology for the combined detection of multiple tumor markers-Huidan Chen(2011)-Data incompleteness(in China).
173、Clinical significance of serum glycoantigen 199, glycoantigen 50 and carcinoembryonic antigen in combination with faecal testing in the diagnosis of rectal cancer-Jintu Chen(2021)-Data incompleteness(in China).
174、Clinical significance of combined serum IL-6, CEA and CA19-9 testing in colorectal cancer

patients-Jingjing Chen(2013)-Data incompleteness(in China).
175、Clinical use of pelvic enhanced CT in combination with serum indices in the comprehensive management of rectal cancer-Lei Chen(2023)-Exclusion criteria met; study participants followed for more than 1 year.
176、The prognostic value of preoperative detection of carcinoembryonic antigen and glycocalyx antigen 199 in patients with stage II-III colorectal cancer-Lei Chen(2015)-Data incompleteness(in China).
177、Study of the use of combined serum tumor marker testing in the clinical diagnosis of colorectal cancer-Li Chen(2019)-The content of this literature is not relevant to the diagnosis of colorectal cancer(in China).
178、Evaluation of the performance of the carcinoembryonic antigen-glycoconjugate antigen 19-9-glycoconjugate antigen 724 combination test for the early detection of colorectal cancer-Meihong Chen(2021)-Data incompleteness(in China).
179、Study of the value of combined serum CA153, CA199, CA125 and CEA testing in the diagnosis of cancer-Min Chen(2015)-The content of this literature is not relevant to the diagnosis of colorectal cancer(in China).
180、Clinical significance of the combined detection of the tumor markers CA19-9, CEA and CA50 in the diagnosis of gastrointestinal tumors-Ning Chen(2013)-Exclusion criteria met; study participants followed for more than 1 year (in China).
181、Value of using TSGF to diagnose colorectal cancer and monitor post-operative treatment effects-Rong Chen(2016)-Data incompleteness(in China).
182、Clinical significance of the tumor markers carcinoembryonic antigen and the combination of cancer antigen 19-9 and cancer antigen 242 in patients with malignant gastrointestinal tumors-Shuping Chen(2011)-Exclusion criteria met; study participants followed for more than 1 year.
183、Clinical value of combined serum HER-2 and CD44 testing in colorectal cancer-Si Chen(2022)-Exclusion criteria met; no tumor marker thresholds specified (in China).
184、Value of serum tumor markers CEA, CA125 and CA199 in predicting clinical response to targeted therapy in patients with advanced colorectal cancer-Suhua Chen(2023)-The content of this literature is not relevant to the diagnosis of colorectal cancer(in China).
185、Clinical value of tumor markers and inflammatory markers in the diagnosis of colorectal cancer and prediction of adverse effects of chemotherapy-Wei Chen(2018)-Data incompleteness(in China).

186、The value of combined CA724, CEA and CA199 testing in the diagnosis of colorectal cancer-Wenping Chen(2020)-Exclusion criteria met; no tumor marker thresholds specified (in China).
187、Clinical value of combined serum PDGF, CEA and CA125 testing in patients with colorectal cancer-Wenqi Chen(2017)-Data incompleteness(in China).
188、Tumor marker co-testing in the diagnosis of gastric and colorectal cancer-Wenzhang Chen(2007)-Exclusion criteria met; study participants followed for more than 1 year (in China).
189、Diagnostic value of combined detection of BAG-1 protein, CA242 and CEA in colorectal cancer-Xiaobo Chen(2016)-Other tumor markers described in the literature(in China).
190、Diagnostic value of a 5-component combination test for digestive tract cancers-Xiaolong

Chen(2013)-Exclusion criteria met; study participants followed for more than 1 year (in China).
191、Serum carcinoembryonic antigen, glycan antigen 199 and glycan antigen 125 with faecal occult blood test in colorectal cancer screening-Xiaoxia Chen(2023)-Data incompleteness(in China).
192、Combined serum macrophage inhibitory factor-1 carcinoembryonic antigen and glycan antigen assay for diagnosis and prognosis of colorectal cancer in the elderly-Shangdang Cai(2016)-Exclusion criteria met; study participants followed for more than 1 year (in China).
193、Clinical application and study of tumor markers in colorectal cancer-Yumei Chen(2005)-No data available(in China).
194、Significance of the tumor markers CEA, CA50 and CA19-9 in the diagnosis of colorectal cancer-Yumei Chen(2005)-Exclusion criteria met; study participants followed for more than 1 year (in China).
195、Diagnostic Value of Combined Neutrophil/Lymphocyte Ratio, Fibrinogen, CA199 and CEA in the Diagnosis of Colorectal Cancer -Yu Chen(2021)-Exclusion criteria met; no tumor marker thresholds specified (in China).
196、The value of combined CEA, CA19-9, CA724 and CA242 testing in colorectal cancer -Zhihong Chen(2013)-Exclusion criteria met; study participants followed for more than 1 year(in China).
197、CT Enhancement in Staging of Colorectal Cancer and the Diagnostic Value of Combined Serum CEA, CA199, CA724 and microRNA-21 in Benign and Malignant Lesions-Zhilin Chen(2023)-Exclusion criteria met; study participants followed for more than 1 year (in China).
198、Clinical significance of serum Golgi phosphorylated protein 3 levels in patients with colorectal cancer-Zhixiong Chen(2018)-Other tumor markers described in the literature(in China).
199、Evaluation of serum CEA and CA199 tests in predicting colorectal cancer recurrence-Zihua Chen(2003)-No data available(in China).
200、Clinical application of combined detection of gastrointestinal tumor markers-Hui Cheng(2006)No data available(in China).
201、A study of the clinical significance of the combined detection of serum CEA and CA724 in colorectal cancer-Jibing Cheng(2016)-Exclusion criteria met; study participants followed for more than 1 year (in China).
202、The Value of Microparticle Enzyme Immunoassay for the Detection of Five Serum Tumor Markers in the Differential Diagnosis of Tumors and Benign Diseases of the Digestive System-Jianping Cheng(2016)-Data incompleteness(in China).
203、The value of combined detection of CEA, CA19-9, CA242 and CA72-4 for the early detection of rectal cancer-Jinling Cheng(2014)-Exclusion criteria met; study participants followed for more than 1 year (in China).
204、An investigation into the value and accuracy of serum tumor marker testing in the adjuvant diagnosis of colorectal cancer-Yao Cheng(2021）-Data incompleteness(in China).

205、Relationship between serum Hcy, CEA and CA199 levels and the development of colorectal

cancer-Zhoujing Cheng(2021)-Meets exclusion criteria; sample size less than 30(in China).
206、Predictive value of serum NLR combined with serum tumor marker testing in postoperative recurrence of colorectal cancer-Yujing Chu(2023)-The content of this literature is not relevant to the diagnosis of colorectal cancer(in China).

207、The value of combined tumor marker testing in the diagnosis of gastrointestinal tumors-Lin Cui(2005)-Meets exclusion criteria; sample size less than 30(in China).
208、Exploring the significance of the tumor-related group of substances (TSGF) assay in aiding the diagnosis of malignant tumors-Guokui Dai(2008)-Other tumor markers described in the literature(in China).

209、Importance of serum tumor marker detection in colorectal cancer-Peng Dai(2008)-Exclusion criteria met; study participants followed for more than 1 year (in China).
210、Clinical significance of CA125, CA199 and CEA in the detection of lung cancer and gastrointestinal tumors-Yu Dai(2001)-Data incompleteness(in China).
211、Detection of tumor markers in colorectal cancer patients using protein microarray technology-Anmei Deng(2002)-Data incompleteness(in China).

212、Role of CEA and CA19-9 in predicting the likelihood of liver metastases in patients with gastrointestinal tumors-Neng Deng(2016)-Data incompleteness(in China).

213、The value of combined TSGF, CEA and CA199 testing in the diagnosis and prognosis of colorectal cancer-Ruilian Deng(2005)-Exclusion criteria met; study participants followed for more than 1 year (in China).
214、Analysis of the value of serum CEA in combination with CA199 in the diagnosis and prognosis of colorectal cancer-Wanfang Deng(2019)Exclusion criteria met; study participants followed for more than 1 year (in China).
215、Diagnostic value of CEA, CA19-9 and CA72-4 tests for gastrointestinal tumors-Dongmei Dong(2006)-Exclusion criteria met; study participants followed for more than 1 year (in China).
216、Combined detection of serum CEA, CA19-9 and CA724 in colorectal cancer patients and its clinical significance-Jinlang Dong(2009)-Exclusion criteria met; no tumor marker thresholds specified.
217、Exploring the value of CEA, CA19-9, CA242 and CA72-4 in colorectal cancer-Xinghui Dong(2005)-Exclusion criteria met; study participants followed for more than 1 year (in China).
218、The value of combined serum tumor marker testing in the diagnosis of colorectal cancer-Biao Du(2021)-Exclusion criteria met; no tumor marker thresholds specified.
219、Analysis of the diagnostic value of serum carcinoembryonic antigen in malignant tumors based on multi-tumor marker protein chip detection-Jia Du(2012)-Data incompleteness(in China).
220、Analysis of the clinical value of a combined serum tumor marker test for the diagnosis of colorectal cancer-Peng Du(2022)-Exclusion criteria met; study participants followed for more than 1 year (in China).
221、Clinical trials for the combined measurement of serum tumor markers in colorectal cancer-Weidong Du(2003)-Exclusion criteria met; study participants followed for more than 1 year (in China).
222、Investigating the role of combined tumor marker testing in the diagnosis of gastrointestinal malignancies-Yun Du(2010)-Data incompleteness(in China).
223、Diagnostic value of serum tumor markers in combination with liver function test for liver metastases from colorectal cancer-Lining Duan(2022)-Exclusion criteria met; study participants followed for more than 1 year (in China).

224、Significance of serum CEA, CA242, CA50 and CA19-9 in the diagnosis and biological characterisation of colorectal cancer-Zexing Duan(2014)-Exclusion criteria met; study participants followed for more than 1 year (in China).
225、The value of four serum tumor markers in monitoring metastasis and recurrence in colorectal cancer-Chenghong Fan(2019)-Exclusion criteria met; no tumor marker thresholds specified.
226、Clinical value of preoperative combined detection of serum tumor markers for the diagnosis of gastrointestinal tumors-Xiaolian Fan (2006)-Data incompleteness (in China).
227、Significance of combined detection of serum TSGF, CA242 and CEA in the diagnosis of colorectal cancer-Chunhua Fang (2007)-No data available (in China).
228、The role of combined tumor marker testing in screening for common malignancies-Ling Fang (2008)-Exclusion criteria met; study participants followed for more than 1 year(in China).
229、Analysis of the clinical value of a combined serum tumor marker test for the diagnosis of colorectal cancer-Ruiming Fang (2021)-Data incompleteness (in China).
230、Analysis of the value of combined faecal occult blood testing and tumor markers in colorectal cancer-Ruiming Fang (2021)-Exclusion criteria met; study participants followed for more than 1 year (in China).
231、The value of combined CEA, CA199 and CA242 testing in early recurrence surveillance after colorectal cancer surgery-Xiuping Fang (2016)-Exclusion criteria met; study participants followed for more than 1 year (in China).
232、Evaluation of the diagnostic value of carcinoembryonic antigen and ferritin in sigmoid colon cancer-Jie Feng (2018)-Exclusion criteria met; study participants followed for more than 1 year (in China).
233、The value of combined serum carcinoembryonic antigen, glycan antigen 199 and tumor M2-type pyruvate kinase testing in the diagnosis of colorectal cancer-Ni Feng (2018)-Exclusion criteria met; study participants followed for more than 1 year (in China).
234、Changes and clinical significance of serum insulin-like growth factor II in elderly patients with colorectal cancer-Xiaoying Feng (2007)-Other tumor markers described in the literature (in China).
235、An investigation into the value of using combined serum tumor marker tests in the clinical diagnosis of colorectal cancer-Shuying Du (2020)-Data incompleteness (in China).
236、Study of the predictive value of faecal occult blood testing in combination with serum CEA and CA199 testing for colorectal cancer recurrence after surgery-Xiaoyun Feng (2023)-Exclusion criteria met; study participants followed for more than 1 year (in China).
237、Evaluation of the diagnostic value of CEA, CA19-9 and CA72-4 coupled tests in differentiating colorectal cancer from non-cancerous disease-Fenghua Fu (2015)-Exclusion criteria met; study participants followed for more than 1 year (in China).
238、Analysis of the clinical value of serum tumor markers in the diagnosis of gastrointestinal tumors-Dan Fu (2012)-Data incompleteness (in China).
239、The value of combined detection of serum dermokine-beta and carcinoembryonic antigen in the diagnosis of colorectal cancer-Handong Fu (2013)-Exclusion criteria met; study participants followed for more than 1 year (in China).
240、Combined detection of tumor markers in colorectal cancer-Hongbing Fu (2012)-Exclusion criteria met; study participants followed for more than 1 year (in China).
241、The value of combined detection of ~ (18)F-FDG PET/CT, CEA and CA199 in the

prognostic assessment of colorectal cancer-Changxia Fu (2020)-No data available (in China).

242、Analysis of the clinical value of Array ELISA for the diagnosis of colorectal cancer using six tumor markers-Guixia Fu (2017)-Meets exclusion criteria; sample size less than 30.

243、Clinical value of microarray enzyme immunoassay for the detection of multiple tumor markers in the diagnosis of colorectal cancer-Chun Gao (2011)-Exclusion criteria met; study participants followed for more than 1 year (in China).

244、Diagnostic value of combined serum carcinoembryonic antigen, glycosylated chain antigen 19-9 and C-reactive protein in colorectal cancer-Jianjun Gao (2014)-Exclusion criteria met; study participants followed for more than 1 year (in China).

245、The value of combined serum tumor marker testing in colorectal cancer screening-LIna Gao (2022)-Data incompleteness (in China).

246、Clinical significance of combined testing of five tumor markers in the diagnosis and follow-up of gastric and colorectal cancer-Zhihai Gao (2012)-Data incompletenesss (in China).

247、Importance of CA19-9 and CEA testing in patients with gastrointestinal malignancies-Zhongdu Gao (2000)-No data available (in China).

248、Diagnostic value of serum leukocyte immunoglobulin-like receptor subfamily B2 levels in patients with colorectal cancer-Yan Geng (2023)-Exclusion criteria met; study participants followed for more than 1 year (in China).

249、Diagnostic value of tumor markers in colorectal cancer assessed by ROC curve and logistic regression-Youquan Geng (2015)-Exclusion criteria met; study participants followed for more than 1 year (in China).

250、The value of SEPT9 gene methylation testing in combination with CA199 and CEA in the diagnosis of colorectal cancer-Xiaohong Gong (2022)-Exclusion criteria met; no tumor marker thresholds specified.

251、Diagnostic test and assessment of five markers of gastrointestinal tumors using electrochemiluminescence detection-Xinjian Gong (2017)-Exclusion criteria met; study participants followed for more than 1 year (in China).

252、The value of faecal SDC2 gene methylation testing in the adjuvant diagnosis of colorectal cancer-Zhiyun Gong (2022)-Exclusion criteria met; study participants followed for more than 1 year (in China).

253、Clinical value of serum CA125 in combination with CA19-9 and CEA testing in the diagnosis of colorectal cancer-Xuejun Gu (2012)-Exclusion criteria met; study participants followed for more than 1 year (in China).

254、Diagnostic value of multiple tumor markers in colorectal cancer analysed by logistic regression and ROC curves-Ping Gu (2007)-Exclusion criteria met; study participants followed for more than 1 year (in China).

255、Changes and clinical significance of serum AFP, CEA, CA19-9 and CA125 in patients with malignant tumors of the digestive system-Yong Gu (2006)-Exclusion criteria met; study participants followed for more than 1 year (in China).

256、Analysis of the clinical validity of combined serum tumor marker tests for the diagnosis of colorectal cancer-Fangfei Guo (2020)-Data incompleteness (in China).

257、Importance of serum free tumor marker co-testing in the diagnosis and management of colorectal cancer-Huiling Guo (2015)-Data incompleteness (in China).

258、Clinical Value of Combined Neutrophil/Lymphocyte Ratio, CEA, and CA19-9 Testing for the

Diagnosis of Rectal Cancer-Jianhui Guo (2021)-Exclusion criteria met; study participants followed for more than 1 year (in China).

259、Study of the association between serum CEA, combined CA19-9 test and colorectal cancer-Juan Guo (2011)-Exclusion criteria met; study participants followed for more than 1 year (in China).

260、Detection and clinical significance of CEA, CA199 and CA242 in colorectal cancer patients-Lin Guo (2010)-The content of this literature is not relevant to the diagnosis of colorectal cancer (in China).

261、The value of combined CEA, CA199 and CA72-4 testing in the diagnosis of colorectal cancer-Qinghua Guo (2013)-Exclusion criteria met; study participants followed for more than 1 year (in China).

262、The value of serum tumor markers in the diagnosis of malignant tumors of the digestive system-Tianli Guo (2010-Data incompleteness (in China).

263、The experience of seven tumor marker co-tests in the diagnosis of colorectal cancer-Wei Guo (2013)-Exclusion criteria met; study participants followed for more than 1 year (in China).

264、Analysis of the clinical value of serum CEA, CA50, CA199 and CA724 in colorectal cancer-Xiaojun Guo (2019)-Data incompleteness (in China).

265、Clinical value of serum tumor markers in combination with serum CA50 and AFP in predicting liver metastases in colorectal cancer-Yanfeng Guo (2021)-Exclusion criteria met; no tumor marker thresholds specified.

266、Analysis of the value of combined alpha-fetoprotein and carcinoembryonic antigen and glycan antigen testing in the diagnosis and management of colorectal cancer-Caihua Ha (2021)-Exclusion criteria met; study participants followed for more than 1 year (in China).

267、Clinical significance of combined detection of four serum tumor markers in colorectal cancer-Hongbing Han (2011)-Exclusion criteria met; study participants followed for more than 1 year (in China).

268、Investigating the diagnostic value of tumor markers in patients with colorectal cancer-Jianrong Han (2013)-Data incompleteness (in China).

269、Expression and clinical significance of CEA and CA19-9 in colorectal cancer tissue-Lu Han (2015)-Data incompletenesss (in China).

270、Clinical significance of combined preoperative serum CEA and CA19-9 testing for prognosis in patients with stage II-III colorectal cancer-Wenfeng Han(2019)-No data available（in China).

271、The role of combined tumor marker testing in the diagnosis of colorectal cancer-Zhongbin Han (2017)-Exclusion criteria met; study participants followed for more than 1 year (in China).

272、Analysis of the correlation between transcolonoscopic diagnosis of colorectal cancer and pathological findings with CEA and CA199 levels-Lin Hao (2019)-Data incompleteness (in China).

273、Analysis of the Diagnostic Value of Serum CEA and CA199 Levels in Gastrointestinal Tumors-Xuehuan He (2018)-Exclusion criteria met; study participants followed for more than 1 year (in China).

274、Investigating the diagnostic value of serum tumor markers TSGF, CEA and CA199 co-testing in colorectal cancer-Yangdong He (2010)-Exclusion criteria met; study participants followed for more than 1 year (in China).

275、Tumor marker tests and their significance in patients with rectal cancer-Faying Han)2011)-Data incompleteness (in China).

276、Tumor marker co-testing in the diagnosis of gastrointestinal tumors-Wangjiao He (2013)-Data incompleteness (in China).
277、Diagnosis of colorectal cancer using medical test analysis of gastrointestinal tumor markers-Wenjing Hou (2022)-Exclusion criteria met; no tumor marker thresholds specified.
278、Clinical applications of combined detection of the tumor markers CEA, CA19-9, CA12-5 and ferritin-Yingnan Hou (2016)-No data available (in China).
279、Co-testing of tumor markers CEA, CA19-9, CA242 in the diagnosis of patients with colorectal cancer-Hongyong Hu (2010)-Exclusion criteria met; study participants followed for more than 1 year (in China).
280、The role of serum tumor markers in the diagnosis of colorectal cancer-Huijing Hu (2019)-Data incompleteness (in China).
281、The value of serum tumor marker testing in the adjuvant diagnosis of colorectal cancer-Shunlin Hu (2019)-Exclusion criteria met; study participants followed for more than 1 year (in China).
282、Diagnostic value of combined serum AFP, CEA, CA199 and CA724 tests for digestive tract malignancies-Siqin Hu (2020)-Exclusion criteria met; study participants followed for more than 1 year (in China).
283、Combined detection of CEA and CA19-9 in the diagnosis of colorectal cancer-Xuefeng Hu (2009)-Meets exclusion criteria; sample size less than 30.
284、Diagnostic value of serum CEA, CA242, CA19-9 and their combination tests in colorectal cancer-Yi Hu (2004)-Meets exclusion criteria; sample size less than 30.
285、Diagnostic value of serum fibrin degradation product DR-70 in combination with conventional tumor markers in colorectal cancer-Nanni Hua (2022)-Exclusion criteria met; study participants followed for more than 1 year (in China).
286、Clinical significance of serum thymidine kinase 1 in the diagnosis of malignant gastrointestinal tumors-Xiaoli Hua (2014)-Meets exclusion criteria; sample size less than 30.
287、Diagnostic value of serum CEA, CA50 and CA19-9 testing in gastrointestinal tumors-Deping Huang (2002)-No data available (in China).
288、Effectiveness of combined serum tumor marker testing in the diagnosis of colorectal cancer-Dengju Huang (2018)-No data available (in China).
289、Analysis of the diagnostic value of combined tumor marker testing in colorectal cancer-Bingxing Huang (2017)-Exclusion criteria met; no tumor marker thresholds specified.
290、Analysis of the combined serum CEA, CA19-9 and CA72-4 test for the diagnosis of colorectal cancer-Ji Huang (2011)-Data incompleteness (in China).
291、Analysis of the effectiveness and detection rate of the combined faecal occult blood and tumor marker test in the diagnosis of colorectal cancer-Jianming Huang (2021)-Meets exclusion criteria; sample size less than 30(in China).
292、The value of tumor markers CEA and CA19-9 in detecting malignant tumors of the digestive system-Kai Huang (2018)-Exclusion criteria met; study participants followed for more than 1 year (in China).
293、Diagnostic value of combined CA19-9 and CEA testing in colorectal cancer-Lijuan Huang (2001)-Data incompleteness (in China).
294、Expression and significance of CD276 and CD133 in combination with CEA and CA199 in colorectal cancer and its precursors-Lina Huang (2018)-Exclusion criteria met; study participants

followed for more than 1 year (in China).

295、Study of the relationship between serum CEA, CA19-9, LN and prognosis in patients with colorectal cancer-Lijuan Huang (2010)-Data incompleteness (in China).

296、Expression and clinical significance of the combined CEA, CA199 assay in colorectal cancer-Qing Huang (2012)-Data incompleteness (in China).

297、The value of combined testing for CEA, CA19-9 and CA72-4 in the diagnosis of colorectal cancer-Qiufang Huang (2007)-Data incompleteness (in China).

298、Investigating the diagnostic value of peripheral blood Septin9 methylation testing in colorectal cancer-Shaoqun Huang (2020)-Exclusion criteria met; study participants followed for more than 1 year (in China).

299、Combined detection of serum CEA, CA125 and CA19-9 in patients with colorectal cancer-Xiuling Huang (2008)-Data incompleteness (in China).

300、How is bowel cancer diagnosed? -Yuqing Huang(2021)-Data incompleteness(in China).

301、Diagnostic value of combined CEA, CA19-9 and CA50 testing in gastrointestinal cancers-Zhongwei Huang (1998)-Meets exclusion criteria; sample size less than 30(in China).

302、The value of the CA724 test in the diagnosis of colorectal cancer-Jinlin Hui (2013)-Exclusion criteria met; study participants followed for more than 1 year (in China).

303、A study of the value of TK1 in colorectal cancer patients-Ying Huo (2015)-Exclusion criteria met; study participants followed for more than 1 year (in China).

304、The role of combined tumor marker testing in the diagnosis of gastrointestinal tumors-Tingting Ji (2004)-Data incompleteness (in China).

305、Clinical significance of combined serum 4 indices in colorectal cancer-Tianxing Ji (2015)-Exclusion criteria met; study participants followed for more than 1 year (in China).

306、The value of combined tumor marker testing in the diagnosis and follow-up of colorectal cancer-Zizhong Ji (2003)-Exclusion criteria met; study participants followed for more than 1 year (in China).

307、Analysis of the application value of introducing serum tumor markers for combined diagnosis in the diagnosis and treatment of early rectal cancer-Leiming Jia (2022)-Exclusion criteria met; study participants followed for more than 1 year (in China).

308、Clinical value of serum CA724 and CA199 in combination with CEA for the diagnosis of colorectal cancer-Peijie Jia (2022.05)-Exclusion criteria met; study participants followed for more than 1 year (in China).

309、Correlation of TK1 with tumor markers in the diagnosis and pathological features of gastrointestinal tumors-Beilei Jiang (2018)-Exclusion criteria met; study participants followed for more than 1 year (in China).

310、Combined detection of serum CEA, CA19-9 and CA242 in colorectal cancer and its clinical significance-Bo Jiang (2006)-Exclusion criteria met; study participants followed for more than 1 year (in China).

311、The value of combined erythrocyte width, platelet width, carcinoembryonic antigen and glycan antigen 199 in the diagnosis and staging of colorectal cancer-Jingbing Jiang (2021)-Data incompleteness (in China).

312、Early warning study of colorectal cancer incidence in high-risk groups using data mining techniques-Yongping Jiang (2020)-Exclusion criteria met; study participants followed for more than 1 year (in China).

313、Three serum tumor markers in colorectal cancer metastasis and recurrence-Nanyan Jiang (2000)-Data incompleteness (in China).

314、Laboratory medicine study of gastrointestinal tumor markers for the diagnosis of colorectal cancer-Tao Jiang (2018)-Exclusion criteria met; study participants followed for more than 1 year (in China).

315、The value of combined serum tumor marker testing in the diagnosis of colorectal cancer-Xiaoting Jiang (2009)-No data available (in China).

316、Diagnostic value of combined serum CEA, CA19-9 and CA242 testing in colorectal cancer and distant organ metastases-Yushi Jiang (2017)-Exclusion criteria met; study participants followed for more than 1 year (in China).

317、Diagnostic value of combined serum tumor marker testing in 30 cases of colorectal cancer-Chunyan Jin (2022)-Data incompleteness (in China).

318、Clinical significance of combined serum CRP, CEA and CA19-9 testing in patients with colorectal cancer-Jinjin Jin (2022)-Exclusion criteria met; study participants followed for more than 1 year (in China).

319、Correlation study between colorectal cancer and tumor markers in Xinjiang-Wuyong Jin (2017)-Exclusion criteria met; study participants followed for more than 1 year (in China).

320、Clinical evaluation of single test and combined test CA199, CEA in colorectal cancer-Xiaoyan Jin (2008)-Data incompleteness (in China).

321、Clinical value of serum TuM2-PK and TSGF in combination with CEA, CA19-9 and CA242 in the diagnosis of colorectal cancer-Weijuan Jin (2015)-Exclusion criteria met; study participants followed for more than 1 year (in China).

322、Serum carcinoembryonic antigen, alpha-fetoprotein and glycan antigen 19-9 levels and clinical significance in patients with colorectal cancer-Xiuping Jing (2020)-Exclusion criteria met; study participants followed for more than 1 year (in China).

323、Value and prognosis of serum microRNA-183, microRNA-141 and hepatocyte growth factor levels in the clinical diagnosis of patients with colorectal cancer-Lihua Ke (2021)-Exclusion criteria met; study participants followed for more than 1 year (in China).

324、Serum tumor markers in the diagnosis of rectal cancer-Diya Ke (2013)-Exclusion criteria met; study participants followed for more than 1 year (in China).

325、Clinical diagnostic value of serum osteopontin levels in patients with colorectal cancer-Chenguang Kou (2022)-Exclusion criteria met; study participants followed for more than 1 year (in China).

326、Analysis of the diagnostic value of gastrointestinal tumor markers in colorectal cancer-Qinglu Kuang (2015)-Data incompleteness (in China).

327、Clinical significance of lipid metabolism indices combined with serum CEA and CA19-9 testing in the diagnosis of colorectal cancer-Yuling Lai (2017)-Data incompleteness (in China).

328、Diagnostic value of tumor markers in colorectal cancer-Yunxing Lai (2012)-Exclusion criteria met; study participants followed for more than 1 year (in China).

329、Diagnostic value of immunohistochemical staining and tumor marker detection in patients with colorectal cancer-Wenzhen Lei (2019)-Meets exclusion criteria; sample size less than 30(in China).

330、Diagnostic value of changes in carcinoembryonic antigen, glycoconjugate antigen 199 and glycoconjugate antigen 724 levels in colorectal cancer-Xing Lei (2018)-Data incompletenesss (in

China).

331、A study of transcolonoscopic sampling and the relationship between CEA and CA125 levels and colorectal cancer-Dongyan Li (2019)-Exclusion criteria met; study participants followed for more than 1 year (in China).

332、Comparative analysis of M_2-PK with CEA, CA19-9 and CA72-4 for the diagnosis of gastrointestinal tumors-Dongmei Li (2007)-Data incompleteness (in China).

333、Analysis of levels of six tumor markers in colorectal cancer patients-Ying Li(2011)-Exclusion criteria met; study participants followed for more than 1 year (in China).

334、Clinical trial and significance of vascular endothelial growth factor detection in perioperative colorectal cancer-Hai Li (2006)-Data incompleteness (in China).

335、The role of CA-19-9, CA-50 and CEA co-testing in monitoring response before and after radiotherapy for rectal cancer-Hanying Li (1996)-Data incompleteness (in China).

336、Colon tissue tumor marker tests for the early detection of colorectal cancer-Jianying Li (2022)-Data incompleteness (in China).

337、Analysis of the diagnostic value of a combined serum multiple tumor marker test for colorectal cancer-Jinkui Li (2018)-Data incompleteness (in China).

338、Construction of a faecal protein Luminex liquid microarray detection system and its value in the early detection of colorectal cancer-Jing Li (2023)-Exclusion criteria met; study participants followed for more than 1 year (in China).

339、Clinical significance of CA242, CA724, CEA single and combined tests in serum of colorectal cancer patients in Qinghai (Xining) region-Juan Li (2011)-Other tumor markers described in the literature (in China).

340、Combined detection of tumor markers in colorectal cancer-Junfeng Li (2016)-Exclusion criteria met; study participants followed for more than 1 year (in China).

341、New research advances the use of tumor markers in the diagnosis of colorectal cancer-Lei li (2019)-No data available (in China).

342、Analysis of the diagnostic value of combined serum CEA, CA199 and CA724 in the diagnosis of colorectal cancer-Lixin Li (2013)-Data incompleteness (in China).

343、Analysis of the application value of a serum tumor marker combination test in the clinical diagnosis of colorectal cancer-Li Li (2019)-Data incompleteness (in China).

344、Diagnostic value and correlation of combined tumor marker and coagulation function tests in colorectal cancer-Longying Li (2022)-Exclusion criteria met; no tumor marker thresholds specified (in China).

345、Clinical value of combined serum CEA, CA19-9 and CRP testing in the diagnosis of colorectal cancer-Ming Li (2010)-Data incompleteness (in China).

346、Analysis of the value of serum CEA, CA199 and CA153 tests in predicting the development of colorectal cancer-Na Li (2016)-Data incompleteness (in China).

347、Expression and clinical significance of thymidine kinase 1, carcinoembryonic antigen and glycan antigen in colorectal cancer-Penghao Li (2023)-Exclusion criteria met; study participants followed for more than 1 year (in China).

348、Diagnostic value of combined serum multiple tumor markers in the diagnosis of colorectal cancer-Qianying Li (2023)-Data incompleteness (in China).

349、Preoperative combined detection of multiple serum tumor markers in the prediction of colorectal cancer liver metastases-Qianying Li (2023-Data incompleteness (in China).

350、Clinical use of serum thymidine kinase assay in patients with colorectal cancer-Qingfeng Li(2012)-Data incompleteness (in China).

351、Correlation of preoperative CEA, CA199, NLR and PLR with the diagnosis and clinicopathological features of colorectal cancer-Shaodong Li (2021)-Exclusion criteria met; study participants followed for more than 1 year (in China).

352、The value of combined serum carcinoembryonic antigen, glycan antigen 199 and reactive protein testing in the diagnosis and prognosis of colorectal cancer-Shaolan Li (2016)-Exclusion criteria met; study participants followed for more than 1 year (in China).

353、To investigate the diagnostic value of serum ESM-1, CEA and CA199 tests in the diagnosis of colorectal cancer-Shilong LI (2019)-Exclusion criteria met; no tumor marker thresholds specified (in China).

354、Combined test for the tumor markers CEA, CA50, CA19-9, CA72-4, CA242 and HSP60 in the diagnosis of colorectal cancer-Shilong Li (2015)-Exclusion criteria met; study participants followed for more than 1 year (in China).

355、Diagnostic value of combined tumor marker testing in colorectal cancer-Suwen Li (2011)-Data incompleteness (in China).

356、Combined detection of tumor markers in malignant gastrointestinal tumors-Wenbing Li (2014)-Exclusion criteria met; study participants followed for more than 1 year (in China).

357、Tumor marker co-testing in gastrointestinal malignancies-Xiangying Li (2012)-Exclusion criteria met; no tumor marker thresholds specified (in China).

358、Role of combined serum tumor marker testing in the diagnosis of colorectal cancer-Xiaoliang Li (2003)-Data incompleteness (in China).

359、Diagnostic value of combined serum CA199 and CEA testing in gastrointestinal cancers-Xiaoyan Li (2010)-Exclusion criteria met; study participants followed for more than 1 year (in China).

360、The value of combined serum tumor marker testing in the adjuvant diagnosis of colorectal cancer-Xin Li (2021)-Exclusion criteria met; no tumor marker thresholds specified (in China).

361、Expression of TPS, CA199 and CEA in the serum of patients with colorectal cancer and its clinical significance-Xuexiang Li (2008)-Exclusion criteria met; study participants followed for more than 1 year (in China).

362、Diagnostic value of Modis-enhanced MRI in combination with serum CEA and CA19-9 in colorectal cancer liver metastases-Yalin Li (2021)-Exclusion criteria met; study participants followed for more than 1 year (in China).

363、Diagnostic value of combined detection of serum CEA, AFP and CA19-9 in colorectal cancer-Yandong Li (2015)-Data incompleteness (in China).

364、Highlighting the clinical value of serum tumor markers in gastric and colorectal cancer-Yan Li (2008)-No data available (in China).

365、Carcinoembryonic antigen, alpha-fetoprotein and glycan antigen 199 in colorectal cancer-Yanxun Li (2019)-Exclusion criteria met; study participants followed for more than 1 year (in China).

366、Use of combined testing for TSGF, IGF-1, CEA and CA19-9 in the diagnosis and prognosis of colorectal cancer-Yanyan Li (2014)-Exclusion criteria met; no information on how the test was performed (in China).

367、Traditional Chinese Medicine Pathogenesis of Colorectal Cancer and Diagnostic Value of

Combined Serum CEA, CA199 and SF Tests in Colorectal Tumors-Yonggang Li (2014)-Exclusion criteria met; study participants followed for more than 1 year (in China).

368、Clinical value of faecal occult blood testing combined with tumor marker detection in colorectal cancer-Zeyong Li (2020)-Data incompleteness (in China).

369、Analysis of the value of the combined detection of alpha-fetoprotein, carcinoembryonic antigen and glycosylated chain antigen 19-9 in the diagnosis of malignant tumors of the digestive system-Zheng Li (2017)-Exclusion criteria met; study participants followed for more than 1 year (in China).

370、A study of the clinical value of combined serum-associated tumor markers in colorectal cancer tumors-Zijian li (2019)-Exclusion criteria met; study participants followed for more than 1 year (in China).

371、Gastrointestinal tumor markers for the diagnosis of colorectal cancer in clinical practice-Jianzhi Liang (2014)-Exclusion criteria met; no tumor marker thresholds specified (in China).

372、Combined detection of Hsp90α, CEA and CA19-9 in the diagnosis of colorectal cancer-Yongming Liang (2018)-Other tumor markers described in the literature (in China).

373、Clinical use of combined CEA, CA19-9 and CA72-4 testing in colorectal cancer-Lin Liao (2013)-Exclusion criteria met; study participants followed for more than 1 year (in China).

374、Value of the combined serum CEA, CA199, CA724 assay in TNM staging of colorectal cancer patients-Pingyue Lin (2015)-Exclusion criteria met; study participants followed for more than 1 year (in China).

375、Investigating the clinical value of combined detection of serum CEA, CA125, CA19-9 and CA72-4 in colorectal cancer-Yunzhi Ling (2019)-Data incompleteness (in China).

376、Study of the diagnostic significance of combined serum multiple tumor markers in the diagnosis of colorectal cancer-Yunzhi Ling (2020)-Exclusion criteria met; study participants followed for more than 1 year (in China).

377、Clinical significance of combined detection of serum CEA, AFP, CA199 and diagnosis of colorectal cancer liver metastases-Hongzhang Liu （2009)-No data available (in China).

378、Diagnostic value of serum CEA and CA19-9 in colorectal cancer with liver metastases-Hu Liu (2019)-Data incompleteness (in China).

379、Clinical significance of tumor markers CEA and CA19-9 in the detection of malignant gastrointestinal tumors-Jin Liu (2008)-Data duplication.

380、Diagnostic value of tumor markers in gastrointestinal tumors evaluated by ROC curve and logistic regression-Qinbo Liu (2008)-Data incompleteness (in China).

381、Clinical value of four serum markers in the diagnosis of colorectal cancer-Liang Liu (2017)-Data incompleteness (in China).

382、Diagnostic value of combined serum multiple tumor markers in the diagnosis of colorectal cancer-Lin Liu (2016)-Exclusion criteria met; study participants followed for more than 1 year (in China).

383、Predictive value of high-resolution T2WI imaging histograms for concurrent liver metastases in rectal cancer-Minglu Liu (2021)-Data incompleteness (in China).

384、Clinical value of serum CA125 in combination with CA19-9 and CEA testing in the diagnosis of colorectal cancer-Xiaolan Liu (2018)-Data incompleteness (in China).

385、Diagnostic analysis of preoperative staging of rectal cancer using intracavitary ultrasound in

combination with CEA and CA199-Xuemei Liu (2016)-Data incompleteness (in China).

386、Expression and significance of serum glycan antigen 199 and carcinoembryonic antigen in patients with liver metastases from colorectal cancer-Yanhang Liu (2018)-Data incompleteness (in China).

387、Analysis of the results of six tumor markers in patients with colorectal cancer-Yiling Liu (2011)-Data incompleteness (in China).

388、Comparison of the diagnostic value of SEPT9 methylation and various serum tumor markers in colorectal cancer-Li Liu (2020)-Data incompleteness (in China).

389、A Study of the Diagnostic Value of Serum Glycoantigen 199, Glycoantigen 50 and Carcinoembryonic Antigen in Combination with Faecal Examination in the Diagnosis of Rectal Cancer-Yi Liu (2019)-Data incompleteness (in China).

390、Significance of TK1 and HSP90α in combination with CEA and CA199 testing in the clinical diagnosis of colorectal cancer-Yuanfang Liu (2019)-Data incompleteness (in China).

391、Clinical value of combined AFP, CEA and CA19-9 testing in the diagnosis of gastrointestinal malignancies-Zhihua Liu (2019)-Data incompleteness (in China).

392、Combined tumor marker testing improves diagnostic positivity in gastrointestinal cancers-Zhixiao Liu (2005)-Data incompleteness (in China).

393、Comparison of CA242 and CA19-9 with CEA co-testing in the overall detection rate of gastrointestinal cancers-Zhuoran Liu (2009)-Data incompleteness (in China).

394、Diagnostic value of HSP90α, CEA and CA-199 in colorectal cancer staging and its clinical significance-Chengjiang Liu (2019)-Data incompleteness (in China).

395、Clinical value of serum tumor markers in the diagnosis of gastrointestinal tumors-Zhaofeng Long (2014)-Data incompleteness (in China).

396、Combined detection of multiple serum markers for the diagnosis of colorectal cancer-Yanhong Lou (2010)-Exclusion criteria met; no tumor marker thresholds specified (in China).

397、Clinical significance of combined detection of serum tumor markers for disease monitoring in colorectal cancer patients-Hongxia Lu (2012)-The content of this literature is not relevant to the diagnosis of colorectal cancer (in China).

398、Diagnostic value of combined serum CEA, CA125, CA199 and CA724 testing in colorectal cancer-Xia Lu (2019)-Data incompleteness (in China).

399、Clinical significance of combined serum CA199 and CEA testing for the early detection of colorectal cancer-Xinqi Lu (2017)-Data incompleteness (in China).

400、Diagnostic value of combined serum multiple tumor marker testing in colorectal cancer-Xumei Lu (2018)-Meets exclusion criteria; sample size less than 30 (in China).

401、Diagnostic analysis of colorectal cancer by combined hydroxybutyrate dehydrogenase, carcinoembryonic antigen and glycan antigen 125 assays-Wan Lu (2023)-Exclusion criteria met; study participants followed for more than 1 year (in China).

402、Study to assess the value of a plasma Septin9 gene methylation test in the diagnosis of colorectal cancer-Feiyan Lu (2023)-Exclusion criteria met; study participants followed for more than 1 year (in China).

403、Diagnostic value of serum PCAT-1 in combination with CEA and CA199 testing in patients with colorectal cancer-Wangfeng Lu (2021)-Exclusion criteria met; study participants followed for more than 1 year (in China).

404、The value of combined serum amino acid and CEA/CA19-9 measurement in the diagnosis of colorectal cancer-Lijuan Lu (2018)-Data incompleteness (in China).

405、Clinical significance and diagnostic value of serum neuron-specific enolase in gastric and colorectal cancer-Hai Luo (2020)-Exclusion criteria met; study participants followed for more than 1 year (in China).

406、The value of serum CEA, CA19-9 in combination with ALP in predicting liver metastases in colorectal cancer-Wei Luo (2020)-Exclusion criteria met; study participants followed for more than 1 year (in China).

407、Clinical value of combined detection of serum CEA and CA19-9 in rectal cancer-Guangdong Lv (2010)-Exclusion criteria met; study participants followed for more than 1 year (in China).

408、Investigating the diagnostic value of combined serum tumor marker testing for gastrointestinal tumors-Jingyang Lv (2017)-Exclusion criteria met; no tumor marker thresholds specified (in China).

409、Diagnostic value of combined detection of tumor markers and inflammatory indicators in colorectal cancer-Zhihao Lv (2019)-Exclusion criteria met; study participants followed for more than 1 year (in China).

410、Correlation Study of the Diagnostic Value and Staging of Colorectal Cancer by Serum Hcy, CEA and CA199-Chenru Ma (2019)-Exclusion criteria met; study participants followed for more than 1 year (in China).

411、Effectiveness of combined serum tumor marker testing in the diagnosis of colorectal cancer-Chi Ma (2022)-Data incompleteness (in China).

412、The value of combined CA19-9, CA242 and CEA testing in the diagnosis of gastrointestinal cancers-Daoxin Ma (2002)-Data incompleteness (in China).

413、The value of non-invasive laboratory tests in the early detection of colorectal cancer-Dejia Ma (2012)-Exclusion criteria met; study participants followed for more than 1 year (in China).

414、The value of the tumor markers CEA, CA199, CA242 and CA50 in the diagnosis of colorectal cancer-Hong Ma (2009)-Exclusion criteria met; study participants followed for more than 1 year (in China).

415、Protein microarray-based combined detection of multiple tumor markers in the diagnosis of gastrointestinal tumors-Jingye Ma (2008)-Data incompleteness (in China).

416、Differences in the expression of serum CA199, CA125 and CEA levels in patients with inflammatory bowel disease and rectal cancer and their clinical significance-Li Ma (2019)-Data incompleteness (in China).

417、Diagnostic Value of Peripheral Blood Neutrophil/Lymphocyte Ratio and Combined Serum Carcinoembryonic Antigen and Glycocalyx Antigen 199 Test for Colorectal Cancer-Xiao Ma (2021)-Data incompleteness (in China).

418、Preoperative combined detection of multiple serum tumor markers in the prediction of colorectal cancer liver metastases-Yunbin Ma (2018)-Exclusion criteria met; study participants followed for more than 1 year (in China).

419、Preoperative serum CEA, CA19-9 and CA50 combination test for predicting liver metastases in colorectal cancer-Yunbin Ma (2018)-Exclusion criteria met; study participants followed for more than 1 year (in China).

420、Evaluation of the diagnostic efficacy of tumor abnormal glycoprotein testing in colorectal cancer-Shuguang Ma (2023)-Exclusion criteria met; study participants followed for more than 1

year (in China).

421、A study of the value of combined serum tumor marker testing in the diagnosis of colorectal cancer-Xiaoyan Mou (2018)-Exclusion criteria met; study participants followed for more than 1 year (in China).

422、Expression and clinical significance of serum CEA, CA125 and CA19-9 in patients with peritoneal metastases from gastrointestinal malignancies-Changsong Mu (2010.04-Data incompleteness (in China).

423、Expression and clinical significance of serum CEA and CA19-9 in colorectal cancer-Changsong Mu (2009)-Data incompleteness (in China).

424、The role of combined serum tumor marker and HSP60 testing in the diagnosis of colorectal cancer-Qingdong Nie (2012)-Exclusion criteria met; study participants followed for more than 1 year (in China).

425、Analysis of the application value of serum CA199, CEA and CA724 in the adjuvant diagnosis of patients with colorectal cancer-Yan Nie (2023)-Data incompleteness (in China).

426、Clinical detection of serum CA50, CA199, CA724 and CEA in patients with colorectal cancer and the significance of their expression-Yuhui Niu (2017)-Exclusion criteria met; no tumor marker thresholds specified (in China).

427、Diagnostic value of AFP, CEA and CA19-9 in the diagnosis of digestive malignancies-Yan Ouyang (2004)-Data incompleteness (in China).

428、A study of the clinical value of combined serum-associated tumor markers in gastrointestinal malignancies-Guilan Pan (2015)-Data incompleteness (in China).

429、Diagnostic value of combined testing for CEA, CA1999 and CA125 in colorectal cancer-Huijuan Pan (2011)-Data incompleteness (in China).

430、Clinical use of combined serum tumor-associated substance assays for common malignancies-Jiwen Pan (2009)-Data incompleteness (in China).

431、Clinical significance of combined testing of gastrointestinal tumor markers-Weihong Pang (2011)-Data incompleteness (in China).

432、Diagnostic value of erythrocyte distribution width in colorectal cancer-Guoze Peng (2020)-Meets exclusion criteria; sample size less than 30 (in China).

433、Correlation analysis between liver metastasis and serum tumor marker expression in colorectal cancer-Hongfei Peng (2014)-Data incompleteness (in China).

434、The value of five preoperative serum tumor markers in the management of rectal cancer-Lei Peng (2013)-Exclusion met; no test performed (in China).

435、Evaluation of the value of applying ROC curves for the use of tumor markers CEA, CA199 and AFP in the diagnosis of rectocolonic tumors-Hua Niu (2005)-Exclusion criteria met; study participants followed for more than 1 year (in China).

436、Clinical application of the combined serum CEA, CA199 and CA242 test in the diagnosis and follow-up of colorectal cancer-Li Peng (2006)-Exclusion criteria met; study participants followed for more than 1 year (in China).

437、Clinical value analysis and prognostic monitoring of a combined test for multiple tumor markers in the diagnosis of colorectal cancer-Xiaolu Peng (2019)-Data incompleteness (in China).

438、Diagnostic value of serum carcinoembryonic antigen, glycan antigen 125 and glycan antigen 19-9 tests for colorectal cancer-Ziqain Peng (2020)-Exclusion criteria met; study participants followed for more than 1 year (in China).

439、Impact of combined serum CEA and CA199 and TuM2-PK levels on diagnostic accuracy in colorectal cancer-Rongrong Qi (2021)-Exclusion criteria met; study participants followed for more than 1 year (in China).
440、Evaluation of TSGF in combination with CEA and CA19-9 in the diagnosis of colorectal cancer-Xinliang Qi (2010)-Exclusion criteria met; no tumor marker thresholds specified (in China).
441、Significance of serum TSGF, CEA and CA199 expression in patients with colorectal cancer-Jun Qian (2008)-Data incompleteness (in China).
442、Value of NLR with FIB and CEA and CA19-9 in colorectal cancer-Xiang Qian (2023)-Exclusion met; no test performed (in China).
443、Diagnostic value of serum serine peptidase inhibitor Kazal type 4 in combination with carcinoembryonic antigen and glycan antigen 199 in colorectal cancer-Chaofeng Qiao (2023)-Exclusion criteria met; study participants followed for more than 1 year (in China).
444、A study of the correlation of serum amyloid A with C-reactive protein, carcinoembryonic antigen and glycoprotein antigen and their value for preoperative staging of patients with colorectal tumors-Haijun Qiao (2016)-Data incompleteness (in China).
445、Significance of combined serum CEA, CA199 and CA724 in the diagnosis of colorectal cancer-Yan Xi (2012)-Data incompleteness (in China).
446、A study of the value of faecal FCP and serum CEA, CA125 and CA19-9 tests in colorectal cancer screening-Lijing Qing (2021)-Exclusion criteria met; no tumor marker thresholds specified (in China).
447、Combined detection of CEA, CA19-9 and CA72-4 in the diagnosis of gastric and colorectal cancer-Bing Qiu (2008)-Exclusion criteria met; study participants followed for more than 1 year (in China).
448、A study of the correlation between preoperative circulating tumor cells and combined serum CEA, CA19-9 and CA125 detection and lymph node metastasis in colorectal cancer-Guisheng Qiu (2023)-Exclusion criteria met; study participants followed for more than 1 year (in China).
449、The value of serum CA125 in combination with CA19-9 and CEA testing in patients with colorectal cancer-Fangzheng Qu (2014)-Data incompleteness (in China).
450、Serum tumor markers in the diagnosis of rectal cancer-Caihong Ren (2014)-Exclusion criteria met; no tumor marker thresholds specified (in China).
451、The value of faecal calreticulin in combination with serum CEA, CA19-9 and CA125 tests for colorectal cancer screening-Chao Ren (2021)-Exclusion criteria met; no tumor marker thresholds specified (in China).
452、The value of combined detection of serum tumor markers in the diagnosis of colorectal cancer-Jindong Ren (2012)-Data incompleteness (in China).
453、The value of faecal and tumor marker testing in the diagnosis and management of colorectal cancer-Jinlan Ruan (2020)-Data incompleteness (in China).
454、Clinical significance of combined detection of CA19-9 and CA72-4 and CEA in serum of patients with colorectal cancer in Qinghai region-Chunxiang Shang (2012)-Meets exclusion criteria; sample size less than 30 (in China).
455、Diagnostic value of combined serum CEA, CA19-9 and CA724 testing in colorectal cancer-Yingyue Sheng (2017)-Data incompleteness (in China).
456、Clinical significance of combined detection of plasma D-dimer, CEA and CA199 in

colorectal cancer patients-Lei Shi (2016)-Exclusion criteria met; study participants followed for more than 1 year (in China).

457、Diagnostic value of AFP, CEA and CA19-9 co-assay in digestive tract malignancies-Min Shi (2001)-Data incompleteness (in China).

458、The value of serum CA19-9 and CEA co-testing in the diagnosis of gastrointestinal tumors-Gong Shi (2005)-Data incompleteness (in China).

459、Detection and clinical significance of CEA, CA199 and CA242 in colorectal cancer patients-Bo Song (2014)-Data incompleteness (in China).

460、The significance of the CA199 test alone or in combination in the diagnosis of colorectal cancer when analysing different expression levels of the tumor marker CEA-Jiachun Song (2019)-Data incompleteness (in China).

461、Study on the use of multi-tumor marker protein microarrays in the diagnosis of colorectal cancer-Yingming Song (2012)-The content of this literature is not relevant to the diagnosis of colorectal cancer (in China).

462、Clinical value of serum CEA, CA19-9 and CA242 in the diagnosis of patients with colorectal cancer-Hongjie Shun (2008)-The content of this literature is not relevant to the diagnosis of colorectal cancer (in China).

463、Clinical value of serum TSGF, CEA and CA199 in the diagnosis of colorectal cancer-Li Shun (2009)-Meets exclusion criteria; sample size less than 30 (in China).

464、The value of CEA, CA19-9 and CA72-4 combination testing in the diagnosis of gastric and colorectal cancer-Libin Shun (2008)-Exclusion criteria met; study participants followed for more than 1 year (in China).

465、The value of combined faecal occult blood testing and tumor markers in colorectal cancer screening-Qiuyue Shun (2022)-Exclusion criteria met; study participants followed for more than 1 year (in China).

466、New research advances the use of tumor markers in the diagnosis of colorectal cancer-Yangbo Tan (2019)-Data incompleteness (in China).

467、Combined detection of four serum tumor markers and faecal occult blood in the diagnosis of Dukes stage B colon cancer-Jun Tang (2017)-Exclusion criteria met; study participants followed for more than 1 year (in China).

468、Expression and significance of CEA, AFP, CA50, CA199 and CA724 in the serum of elderly colorectal cancer patients-Jun Tang (2019)-Exclusion criteria met; study participants followed for more than 1 year (in China).

469、Value of the combined serum CEA, CA199, CA125 and CA724 test in the diagnosis of colorectal cancer-Leliu Tang (2020)-Data incompleteness (in China).

470、Combined detection of serum CGA, CA19-9 and CEA in colorectal cancer-Jing Tang (2007)-Data incompleteness (in China).

471、Clinical significance of lipid metabolism indices in combination with serum CEA, CA72-4 and CA19-9 tests in the diagnosis of colorectal cancer-Xujun Tang (2016)-Exclusion criteria met; study participants followed for more than 1 year (in China).

472、Serum and faecal CEA, CA199 and AFP in the adjuvant diagnosis of colorectal cancer-Yang Tang (2021)-Exclusion criteria met; study participants followed for more than 1 year (in China).

473、Expression and clinical significance of LncRNA SNHG11 in serum of colorectal cancer patients-Shanneng Tao (2020)-Exclusion criteria met; no tumor marker thresholds specified (in

China).

474、The value of magnetic resonance imaging combined with serum CEA and CA125 detection in the diagnosis of colorectal cancer-Enzhao Tian (2018)-Other tumor markers described in the literature (in China).

475、Study of the diagnostic value of combined CEA, CA199, CA242 and CA724 testing in colorectal cancer-Hua Tian (2013)-Exclusion criteria met; study participants followed for more than 1 year (in China).

476、Value and significance of combined CA19-9 and CEA testing in patients with gastrointestinal malignancies-Xuezhi Tian (2000)-Data incompleteness (in China).

477、Study of the clinical diagnostic value of the combined detection of different tumor markers in malignant tumors-Wenzhou Tan (2010)-Data incompleteness (in China).

478、Clinical significance of combined detection of multiple tumor markers in colorectal cancer sera-Zhijun Tian (2011)-Data incompleteness (in China).

479、Diagnostic value of combined CEA, CA724 and CA153 testing in colorectal cancer-Meihui Tong (2018)-Exclusion criteria met; study participants followed for more than 1 year (in China).

480、Changes and clinical significance of serum CEA, CA199 and CA125 before and after surgery for colorectal malignancies-Biyu Wang (2011)-Data incompleteness (in China).

481、The value of the serum multi-tumor marker protein chip detection system in the diagnosis of colorectal cancer-Bingji Wang (2010)-Exclusion criteria met; no tumor marker thresholds specified (in China).

482、The value of combined serum tumor marker testing in the diagnosis of colorectal cancer-Chunyang Wang (2019)-Data incompleteness (in China).

483、Significance of combined carcinoembryonic antigen and tumor antigen 19-9 testing in colorectal cancer-Chengmou Shun (2010)-Data incompleteness (in China).

484、Analysis of the value of serum CEA, CA19-9 and CA242 in the diagnosis of colorectal cancer-Fei Wang (2018)-Exclusion criteria met; study participants followed for more than 1 year (in China).

485、The value of the tumor markers CEA, AFP, CA50, CA19-9 and CA72-4 in the diagnosis of gastrointestinal malignancies-Gang Wang (2012)-Exclusion criteria met; study participants followed for more than 1 year (in China).

486、Diagnostic value of combined CA19-9, CEA, β_2-m and SF RIA in the diagnosis of gastrointestinal malignancies-Guangxian Wang (1995)-Data incompleteness (in China).

487、Diagnostic value of combined tumor marker testing in gastrointestinal cancers-Guijuan Wang (2007)-No data available (in China).

488、Clinical significance of plasma heat shock protein 90α in the diagnosis of colorectal cancer and monitoring of chemotherapy response-Haixia Wang ((2020)-Exclusion criteria met; study participants followed for more than 1 year (in China).

489、Combined serum CRP, CA19-9 and CEA test for the preoperative diagnosis of colorectal cancer-Haiyan Wang (2022)-Exclusion criteria met; no tumor marker thresholds specified (in China).

490、Analysis of the expression and clinical significance of serum CEA and CA19-9 in colorectal cancer-Hao Wang (2011)-Exclusion criteria met; study participants followed for more than 1 year (in China).

491、Clinical value of cyclic RNA HIPK3, 001569 in combination with CEA and CA19-9 in

colorectal cancer-Hong Wang (2019)-Data incompleteness (in China).

492、Expression of protomyosin-4 in combination with CEA and CA19-9 in colorectal cancer and its clinical significance-Huaguo Wang (2019)-Exclusion criteria met; no tumor marker thresholds specified (in China).

493、Evaluation of serum CEA, CA242 measurement in the diagnosis of colorectal cancer-Huaizhi Wang (1998)-Other tumor markers described in the literature (in China).

494、Clinical significance of gastrointestinal tumor markers in the diagnosis of colorectal cancer-Hui Wang (2018)-Data incompleteness (in China).

495、The value of combined detection of serum CEA, CA19-9 and CA72-4 in the diagnosis of colorectal cancer in the elderly-Jian Wang (2014)-Exclusion criteria met; study participants followed for more than 1 year (in China).

496、Clinical value of combined serum CEA, CA199, CA242 and CA724 testing in the diagnosis of colorectal cancer-Kexue Wang (2016)-Exclusion criteria met; study participants followed for more than 1 year (in China).

497、The value of combined stool and tumor marker testing in the diagnosis and management of colorectal cancer-Lei Wang (2018)-Data incompleteness (in China).

498、Diagnostic value of the combination of CEA, CA199, cytokeratin fragment 19 and CK in colorectal cancer-Li Wang (2013)-Data incompleteness (in China).

499、Significance of combined serum CEA, CA19-9 and CA724 testing in patients with colorectal cancer-Liangyi Wang (2013)-Data incompleteness (in China).

500、Serum CEA, AFP, CA19-9, CA72-4 coupling assay for the differential diagnosis of benign and malignant diseases of the digestive tract. -Linglong Wang (2011)-Data incompleteness (in China).

501、Clinical value of combined AFP, CEA, CA199 and CA724 testing in gastrointestinal malignancies-Mei Wang (2011)-Data incompleteness (in China).

502、The value of TK1, CEA and CA199 tests in the diagnosis of colorectal cancer-Min Wang (2017)-Data incompleteness (in China).

503、Predictive Value of Preoperative Serum CEA, CA19-9, CA50, AFP and TPS Tests for Liver Metastases in Colorectal Cancer-Na Wang (2021)-Data incompleteness (in China).

504、The value of combined tumor marker testing in the diagnosis of malignant gastrointestinal tumors-Neng Wang (2022)-Exclusion criteria met; study participants followed for more than 1 year (in China).

505、The value of serum CEA and CA199 combined with ESM-1 test in the diagnosis of colorectal cancer-Ping Wang (2022)-Exclusion criteria met; no tumor marker thresholds specified (in China).

506、Correlation of ~ (18)F-deoxyglucose PET/CT metabolic parameters with clinical features and serum CEA, CA199 and VEGF in colorectal cancer-Quan Wang (2020)-Data incompleteness (in China).

507、The role of serum CA19-9, CEA and CA50 in the diagnosis of colorectal cancer-Renhua Wang (1998)-Data incompleteness (in China).

508、Significance of soluble cytotoxic T-lymphocyte-associated antigen-4 in combination with carcinoembryonic antigen and glycoconjugate antigen 19-9 assays in the diagnosis of colorectal cancer-Ruiping Wang (2017)-Exclusion criteria met; study participants followed for more than 1 year (in China).

509、Clinical analysis of serum carcinoembryonic antigen and glycoconjugate antigen 199 and glycoconjugate antigen 724 levels for the diagnosis of colorectal cancer-Shanshan Wang (2020)-The content of this literature is not relevant to the diagnosis of colorectal cancer (in China).

510、Diagnostic value of combined detection of multiple tumor markers in colorectal cancer-Shaoliang Wang (2022)-Exclusion criteria met; study participants followed for more than 1 year (in China).

511、A study of the correlation between D-dimer and tumor markers in colorectal cancer-Wenjun Wang (2018)-Data incompleteness (in China).

512、Clinical significance of CEAmRNA expression and serum CEA and related protein detection in colorectal cancer patients-Wenyuan Wang (2011)-No data available (in China).

513、A study of the clinical value of elevated macrophage inhibitory factor-1 in the serum of patients with colorectal cancer-Xiaobing Wang (2011)-Exclusion criteria met; no tumor marker thresholds specified (in China).

514、Clinical diagnostic value of ~ (18)F-FDG PET-CT, CEA, CA199 combined test in colorectal cancer-Xiaolin Wang (2019)-Exclusion criteria met; study participants followed for more than 1 year (in China).

515、Relationship between serum TSGF levels and diagnosis and postoperative outcome in colorectal cancer-Xin Wang (2013)-Data incompleteness (in China).

516、Analysis of the value of serum tumor marker association testing in the diagnosis of colorectal cancer-Xinmei Wang (2019)-Data incompleteness (in China).

517、Diagnostic value of circulating fibrinogen to albumin ratio (FAR) and circulating fibrinogen to prealbumin ratio (FPR) in combination with carcinoembryonic antigen (CEA) in colorectal cancer-Yan Wang (2022)-Exclusion criteria met; no tumor marker thresholds specified (in China).

518、Diagnostic value of combined plasma FIB, CEA and CA199 testing in colorectal cancer and its relationship to TNM staging-Yanmin Wang (2023)-Exclusion criteria met; study participants followed for more than 1 year (in China).

519、Diagnostic study of colorectal cancer using combined faecal occult blood and tumor marker testing-Yang Wang (2020)-Data incompleteness (in China).

520、Clinical use of serum CEA, CA199 and CA242 co-assay in the diagnosis of colorectal cancer-Ying Wang (2008)-Data incompleteness (in China).

521、The value of serum tumor markers detected by autoluminescence in the diagnosis of colorectal cancer-Yongqing Wang (2019)-No data available (in China).

522、The Value of the C-12 Multi-Tumor Marker Protein Chip Detection System in the Diagnosis and Disease Monitoring of Colorectal Cancer-Yong Wang (2011)-Data incompleteness (in China).

523、Value of CEA, CA199 and CA242 testing in colorectal cancer patients-Yong Wang (2005)-Meets exclusion criteria; sample size less than 30 (in China).

524、Detection and clinical significance of serum CA72-4, CA199 and CEA in patients with colorectal cancer-Yong Wang (2015)-Exclusion criteria met; study participants followed for more than 1 year (in China).

525、Clinical significance and diagnostic value of combined testing of 4 coagulation and tumor markers in colorectal cancer-Yuying Wang (2019)-Exclusion criteria met; no tumor marker thresholds specified (in China).

526、Diagnostic value of carcinoembryonic antigen and glycoprotein antigen 19-9 in patients with colorectal cancer-Zhaopu Wang (2016)-Exclusion criteria met; no tumor marker thresholds

specified (in China).

527、Early diagnostic value of diffusion-weighted magnetic resonance imaging in combination with tumor markers for preoperative liver metastases in colorectal cancer-Renrui Wei (2021)-No data available (in China).

528、Detection and clinical significance of CEA, CA199 and CA242 in colorectal cancer patients-Bo Wei (2020)-Data incompleteness (in China).

529、Analysis of the value of the combined CEA, AFP, CA125 and CA199 test for the early detection of colorectal cancer-Li Wei (2022)-Exclusion criteria met; study participants followed for more than 1 year (in China).

530、Early diagnostic value of combined detection of multiple tumor markers for gastrointestinal malignancies in a healthy population-Shuangqin Wei (2018)-Meets exclusion criteria; sample size less than 30 (in China).

531、The value of tumor-specific growth factor in combination with CA19-9 and CEA to improve the diagnosis of colorectal cancer-Xiangsong Wei (2004)-Exclusion criteria met; study participants followed for more than 1 year (in China).

532、Value of combined serum tumor marker tests in the diagnosis of colorectal cancer-Yaping Wei (2022)-Data incompleteness (in China).

533、Serum miR-92a-1 expression levels in colorectal cancer patients and their diagnostic significance-Xiaoyu Wu (2021)-Exclusion criteria met; no tumor marker thresholds specified (in China).

534、Clinical use of four tumor markers in the diagnosis of rectal cancer-Cailan Wu (2009)-Data incompleteness (in China).

535、Diagnostic value of 5 tumor marker tests-Cailan Wu (2004)-Data incompleteness (in China).

536、Analysis of the clinical use of serum tumor markers in elderly patients with digestive malignancies-Daohong Wu (2004)-Data incompleteness (in China).

537、Relationship between serum CEA, CA19-9 and CA724 testing and the degree of differentiation and clinical stage of colorectal cancer-Daohong Wu (2004)-Data incompleteness (in China).

538、The value of combined testing for multiple tumor markers in the diagnosis of gastrointestinal tumors-Juan Wu (2020)-Exclusion criteria met; study participants followed for more than 1 year (in China).

539、A study of the value of colorectal cancer tumor markers combined with multislice spiral CT in the diagnosis of colorectal cancer in the elderly-Ming Wu (2018)-Data incompleteness (in China).

540、Diagnostic value of CEA, CA19-9 and CA72-4 combination testing in digestive tract cancers-Yanfang Wu (2011)-Exclusion criteria met; study participants followed for more than 1 yea r(in China).

541、The value of serum tumor markers in the diagnosis of five gastrointestinal tumors-Weiqing Wu (2016)-Exclusion criteria met; study participants followed for more than 1 year (in China).

542、Analysis of the diagnostic value of miR-92a-1 in combination with serum CEA and CA-199 detection in colorectal cancer-Xiaozhen Wu (2023)-Exclusion criteria met; no tumor marker thresholds specified (in China).

543、Diagnostic and differential diagnostic value of combined D-dimer, CEA, CA242 and CA50 tests in colorectal cancer-Yuchen Wu (2019)-No data available (in China).

544、Diagnostic value of serum DR-70 in combination with CEA and CA199 in colorectal cancer-Zilin Wu (2022)-Exclusion criteria met; no tumor marker thresholds specified (in China).

545、Diagnostic value of CEA, CA199 and CA125 tests in patients with colorectal cancer-Hongmei Wu (2014)-Data incompleteness (in China).

546、Prognostic value of combined detection of multiple preoperative serum tumor marker levels for clinical staging of colorectal cancer-Longjie Xia (2016)-The content of this literature is not relevant to the diagnosis of colorectal cancer (in China).

547、Analysis of the diagnostic value of combined serum CEA, CA125, CA19-9 and CYFRA-21 testing in colorectal cancer-Yingying Xia (2021)-Exclusion criteria met; study participants followed for more than 1 year (in China).

548、The value of detecting serum CEA, CA19-9, CA242 and CA72-4 for the clinical diagnosis of colorectal cancer-Jiaqiang Xiao (2014)-Exclusion criteria met; study participants followed for more than 1 year (in China).

549、The role of serum tumor markers in the diagnosis of colorectal cancer-Ran Xiao (2019)-Data incompleteness (in China).

550、Clinical significance of the combined measurement of serum CEA, CA19-9 and CA242 in patients with colorectal cancer-Yapin Xiao (2006)-Data incompleteness (in China).

551、Diagnostic value of sIL-33 in combination with CEA and CA19-9 testing for colorectal cancer-Quanqin Xie (2015)-Exclusion criteria met; study participants followed for more than 1 year (in China).

552、Diagnostic value of sCTLA-4 in combination with CEA and CA19-9 testing for colorectal cancer-Quanqin Xie (2015)-Exclusion criteria met; no tumor marker thresholds specified (in China).

553、Expression and significance of serum AFP, CEA and CA19-9 in patients with colorectal cancer-Rongbing Xie (2020)-Data incompleteness (in China).

554、Clinical value of serum tumor markers and HSP90α combined detection in the diagnosis of colorectal cancer-Yuling Xie (2020)-Exclusion criteria met; study participants followed for more than 1 year (in China).

555、Analysis of the diagnostic value of serum CA125 in combination with CA19-9 and CEA tests for colorectal cancer-Chunrong Xiong (2016)-Exclusion criteria met; study participants followed for more than 1 year (in China).

556、Investigating the diagnostic value of combined tumor marker testing (CA153, CA199, CA125, CEA) for breast, ovarian and colorectal cancer-Juan Xiong (2017)-Data incompleteness (in China).

557、Diagnostic value of combined testing for CEA, CA199 and CA125 in colorectal cancer-Ailei Xu (2014)-Data incompleteness (in China).

558、Expression and clinical value of combined CEA and CA199 testing in colorectal cancer-Jiawei Xu (2019)-Data incompleteness s(in China).

559、Clinical significance of combined serum CEA, CA199 and CA125 testing in patients with colorectal cancer-Lan Xu (2006)-Exclusion criteria met; no tumor marker thresholds specified (in China).

560、Analysis of the value of 4 serum tumor marker levels in different stages of colorectal cancer-Lu Xu (2019)-Exclusion criteria met; no tumor marker thresholds specified (in China).

561、Changes in serum apolipoprotein expression in patients with colorectal cancer and clinical significance-Runhao Xu (2023)-Exclusion criteria met; no tumor marker thresholds specified (in

China).

562、Evaluation of the diagnostic sensitivity of four tumor glycan antigens for colon and rectal cancer-Xiaohong Xu (1998)-Data incompleteness (in China).

563、CEA, CA724 and CA199 for the diagnosis of gastrointestinal malignancies-Yuehua Xu (2006)-Data incompleteness (in China).

564、The value of combined CEA and CA19-9 testing in the diagnosis of colorectal cancer-Zhiyun Xu (2011)-Exclusion criteria met; study participants followed for more than 1 year (in China).

565、Preoperative diagnostic value of combined serum CRP, CA19-9 and CEA testing in colorectal cancer-Jiayi Xu (2017)-Exclusion criteria met; no tumor marker thresholds specified (in China).

566、The Value of Multi-Tumor Marker Protein Microarrays for the Detection of CA242 in the Diagnosis of Malignant Tumors-Mingfang Xu (2012)-The content of this literature is not relevant to the diagnosis of colorectal cancer (in China).

567、Diagnostic Value of Combined Serum AFP, CEA, CA125 and CA199 Tests for Malignant Tumors of the Digestive System-Jinfang Xue (2014)-Data incompleteness (in China).

568、The value of routine blood and coagulation tetrad in combination with CEA and CA19-9 in the diagnosis of colorectal cancer-Zhifeng Xue (2022)-Exclusion criteria met; no tumor marker thresholds specified (in China).

569、Diagnostic value of combined serum CEA, CA125 and AFP testing in colorectal cancer-Zhengwen Yan (2021)-Other tumor markers described in the literature (in China).

570、Clinical value of serum CA125, CA19-9 and CEA tests in the diagnosis of colorectal cancer-Shide Yan (2011)-Exclusion criteria met; study participants followed for more than 1 year (in China).

571、Diagnostic value of MSCT in combination with serum CAA199 and CEA tests for colorectal cancer and metastasis-Jindou Yang (2022)-Exclusion criteria met; study participants followed for more than 1 year (in China).

572、Clinical value of combined CTC, CEA and CA125 testing in colorectal cancer-Chaomei Yang (2021)-Other tumor markers described in the literature (in China).

573、Clinical significance of heat shock protein 90α in chemotherapy monitoring in colorectal cancer-Chaoxia Yang (2021)-The content of this literature is not relevant to the diagnosis of colorectal cancer (in China).

574、The value of combined testing of blood lipid levels and serum tumor markers in the diagnosis of colorectal cancer-Chuangjie Yang (2018)-Exclusion criteria met; study participants followed for more than 1 year (in China).

575、Clinical significance of combined serum CEA, CA-125, CA-153, CA-199 and CA242 assays for the diagnosis of gastrointestinal tumors-Dehui Yang (2007)-Data incompleteness (in China).

576、Gastrointestinal tumor markers for the diagnosis of colorectal cancer in clinical practice-Fang Yang (2014)-Data incompleteness (in China).

577、The value of combined serum tumor marker testing in the diagnosis of colorectal cancer-Fubiao Yang (2011)-Data incompleteness (in China).

578、The screening value of serum T uM 2-PK, CEA, CA19-9 and CA72-4 for colorectal cancer-Gang Yang (2017)-Exclusion criteria met; study participants followed for more than 1 year (in China).

579、Diagnostic value of combined serum AFP, CEA and CA19-9 measurement in gastrointestinal

malignancies-Hao Yang (2005)-Data incompleteness (in China).

580、Diagnostic and prognostic value of CEA and CA19-9 in patients with colorectal cancer-Hua Yang (2017)-Data incompletenesss (in China).

581、Clinical significance of combined serum CA242, CEA and CA199 testing in the diagnosis and management of pancreatic and rectal cancer-Liting Yang (2008)-Data incompleteness (in China).

582、Tumor markers combined with pathology for prognosis of peritoneal metastases in colorectal cancer-Mingrui Yang (2018)-Exclusion criteria met; study participants followed for more than 1 year (in China).

583、Diagnostic value of serum CEA, CA19-9 and faecal occult blood tests in colorectal cancer-Tengjian Yang (2021)-Data incompleteness (in China).

584、The value of combined detection of serum CEA, VEGF and CA19-9 in the diagnosis and management of colorectal cancer-Xiaowen Yang (2009)-Exclusion criteria met; study participants followed for more than 1 year (in China).

585、Clinical significance of serum CA125 in combination with CA19-9 and CEA testing in the diagnosis of colorectal cancer-Xiuzhen Yang (2021)-Exclusion criteria met; study participants followed for more than 1 year (in China).

586、Multi-tumor marker C12 protein microarray valuable in diagnosing advanced gastrointestinal cancers-Xueqin Yang (2008)-Data incompleteness (in China).

587、The value of serum tumor marker co-testing in the diagnosis of colorectal cancer-Aiping Yao (2010)-Exclusion criteria met; study participants followed for more than 1 year (in China).

588、Analysis of serum carcinoembryonic antigen and cancer-associated glycoantigen 125 and 199 levels in patients with tumors-Huisheng Yao (2012)-Data incompleteness (in China).

589、Clinical significance of serum tumor marker testing in patients with colorectal cancer-Pingjiang Ye (2016)-Exclusion criteria met; study participants followed for more than 1 year (in China).

590、Investigating the value of serum tumor markers in the clinical diagnosis of colorectal cancer-Honglei Yin (2012)-Exclusion criteria met; study participants followed for more than 1 year (in China).

591、Significance of combined detection of CEA and CA19-9 in malignant tumors of the digestive system-Caiming Yu (2016)-Exclusion criteria met; study participants followed for more than 1 year (in China).

592、The Significance of Serum CEA, CA199 and CA125 Levels in the Diagnosis of Malignant Tumors by Chemiluminescence Immunoassay-Jianying Yu (2004)-Data incompleteness (in China).

593、The value of combined detection of multiple serological tumor markers in the diagnosis and management of gastrointestinal tumors-Miaomiao Yu (2014)-Data incompleteness (in China).

594、Diagnostic value of glycan antigen CA125 CA199 CA724 carcinoembryonic antigen in colorectal cancer-Xiaofu Yu (2019)-Exclusion criteria met; study participants followed for more than 1 year (in China).

595、Clinical study of the combined serum CEA, CA19-9 and CA72-4 test for the diagnosis of colorectal cancer-Lin Yue (2006)-Exclusion criteria met; no tumor marker thresholds specified (in China).

596、The value of combined serum CEA, CA19-9 and IL-8 testing in the diagnosis of colorectal cancer-Chunyan Zhang (2014)-Exclusion criteria met; study participants followed for more than 1

year (in China).

597、Clinical analysis of combined serum tumor marker tests for the diagnosis of colorectal cancer-Guiyang Zhang (2012)-Exclusion criteria met; study participants followed for more than 1 year (in China).

598、Combined serum CEA, CA19-9, CA-50 test for the diagnosis of colorectal cancer-Yaoyong Yang (2011)-Exclusion criteria met; study participants followed for more than 1 year (in China).

599、The value of serum CA125, CA19-9 and CEA tests in the diagnosis of colorectal cancer-Hailin Zhang (2012)-Data incompleteness (in China).

600、Clinical significance of tumor protein markers in the detection of colorectal cancer-Haiqing Zhang (2007)-Exclusion criteria met; study participants followed for more than 1 year (in China).

601、Analysis of the application value of serum tumor marker detection in the diagnosis of malignant tumors of the digestive tract-Hongxia Zhang (2013)-Data incompleteness (in China).

602、Expression of five tumor markers in gastric and colorectal cancer-Jian Zhang (2010)-Data incompleteness (in China).

603、The value of CA19-9 in conjunction with CEA and β2-MG in the diagnosis of gastric and rectal/colon cancer-Jinchi Zhang (2005)-Data incompleteness (in China).

604、The Value of Combined CEA and CA19-9 Testing in the Diagnosis and Response to Treatment of Rectal Cancer-Jing Zhang (2016)-Data incompleteness (in China).

605、Tumor marker co-testing in the diagnosis of malignant tumors-Guoqing Zhang (2012)-Data incompleteness (in China).

606、Combined serum CEA, CA19-9, CA-50 test for the diagnosis of colorectal cancer-Junhe Zhang (2009)-Data incompleteness (in China).

607、Analysis of the diagnostic value of four serum tumor markers in patients with colorectal cancer-Jun Zhang (2019)-Exclusion criteria met; study participants followed for more than 1 year (in China).

608、Serum VEGF, CA19-9 and OPN levels in the clinical diagnosis of colorectal cancer and analysis of surgical outcomes-Kai Zhang (2015)-Other tumor markers described in the literature (in China).

609、The value of combined serum CEA, CA19-9 and CA125 testing for the diagnosis of gastrointestinal malignancies-Lidong Zhang (2003)-Exclusion criteria met; study participants followed for more than 1 year (in China).

610、Expression of serum Nup88, CEA and CA19-9 in colorectal cancer and its clinical significance-Lijing Zhang (2011)-Data incompletenesss (in China).

611、Significance of combined serum CEA, CA19-9 and CA72-4 testing in the early detection and diagnosis of colorectal cancer-Liping Zhang (2020)-Exclusion criteria met; study participants followed for more than 1 year (in China).

612、Analysis of serum glycoconjugate antigen 125, glycoconjugate antigen 19-9 and carcinoembryonic antigen test results in patients with colorectal cancer-Min Zhang (2018)-Data incompleteness (in China).

613、Clinical use of tumor markers in colorectal cancer-Mingjian Zhang (2016)-Data incompleteness (in China).

614、Significance of changes in faecal tests and serum levels of five tumor markers including CEA, CA199, CA242, CA724 and TPA in patients with colorectal cancer-Naidan Zhang (2018)-Data incompleteness (in China).

615、Clinical significance of serum CA125 in combination with CA19-9 and CEA testing in the diagnosis of colorectal cancer-Ning Zhang (2014)-Data incompleteness (in China).

616、Diagnostic value of combined tumor marker tests in elderly patients with colorectal cancer-Qing Zhang (2012)-Exclusion criteria met; study participants followed for more than 1 year (in China).

617、The Value of Combined Serum Glycan Antigen and Carcinoembryonic Antigen Testing in the Diagnosis of Malignant Tumors-Sha Zhang (2014)-Data incompleteness (in China).

618、Diagnostic value of combined serum tumor marker testing in rectal cancer-Wei Zhang (2017)-Data incompleteness (in China).

619、The value of the tumor markers CEA, CA50 and CA19-9 in the diagnosis of colorectal cancer-Weili Zhang (2017)-Exclusion criteria met; study participants followed for more than 1 year (in China).

620、Study of the diagnostic value of serum tumor markers in colorectal cancer-Xuelan Zhang (2014)-Data incompleteness (in China).

621、Combined detection of CEA and CA19-9 in the diagnosis of colorectal cancer-Zegang Zhang (2007)-Exclusion criteria met; study participants followed for more than 1 year (in China).

622、Clinical significance of combined serum tumor marker testing in rectal cancer-Zhen Zhang (2015)-Data incompleteness (in China).

623、Evaluation of the diagnostic value of CEA, CA19-9 and CA72-4 in colorectal cancer-Lijiu Zhang (2005)-Data incompleteness (in China).

624、Analysis of the association between tumor marker levels and patient prognosis after radical surgery for colorectal cancer-Jia Zhao (2023)-Data incompleteness (in China).

625、Diagnostic value of tumor markers CEA and CA19-9 in colorectal cancer-Xuemei Zhao (2013)-Data incompleteness (in China).

626、Clinical use of three tumor markers (CEA, CA199, CA724) in the diagnosis of rectal cancer-Youliang Zhao (2015)-Exclusion criteria met; study participants followed for more than 1 year (in China).

627、To investigate the value of combined CA-50, CEA, AFP and β_2-MG testing in improving the detection rate of gastrointestinal malignancies. -Wenyou Zheng (1999)-Data incompleteness (in China).

628、The value of tumor markers and combined faecal tests in the diagnosis and management of colorectal cancer-Lei Zhong (2019)-Exclusion criteria met; no method of detection (in China).

629、Diagnostic value of CEA, CA199, CA724 and CA242 for colorectal cancer-Maiwen Zhong (2022)-Exclusion criteria met; study participants followed for more than 1 year (in China).

630、Clinical significance of combined CEA and CA199 testing in colorectal cancer-Qifen Zhong (2009)-Exclusion criteria met; study participants followed for more than 1 year (in China).

631、A study of the clinical significance of different combinations of CEA, CA19-9, CA125 and CA72-4 in the diagnosis of colorectal cancer-Wa Zhong (2014)-Data incompleteness (in China).

632、Combination testing of tumor markers in patients with colorectal cancer and its diagnostic value-Fufeng Zhao (2011)-Exclusion criteria met; study participants followed for more than 1 year (in China).

633、Diagnostic utility of multiple tumor markers in combination in colorectal cancer-Haihua Zhou (2017)-Data incompleteness (in China).

634、Analysis of the value of a serum tumor marker combination test in the clinical diagnosis of

colorectal cancer-Lifang Zhou (2018)-Data incompleteness (in China).

635、Diagnostic value of combined detection of serum CEA_CA19-9_CA72-4 in digestive tract cancers-Lili Zhou (2013)-Data incompleteness (in China).

636、To investigate the value of CEA, CA19-9, CA242 and CA72-4 in the early detection of rectal cancer-Yongling Zhou (2015)-Data incompleteness (in China).

637、The value of CEA, CA19-9, CA72-4 and CA242 co-testing in the diagnosis and management of rectal cancer-Chuanjin Zhu (2007)-Exclusion criteria met; study participants followed for more than 1 year (in China).

638、The value of serum CEA and CA19-9 detection in colorectal cancer-Huidong Zhu (2013)-Data incompleteness (in China).

639、Exploring the application value of serum tumor marker combination testing in the clinical diagnosis of colorectal cancer-Jianfeng Zhu (2018)-Data incompleteness (in China).

640、Value of CA724, CA125, CA199 and CEA in the diagnosis of colorectal cancer-Shanling Zhu (2013)-Exclusion criteria met; study participants followed for more than 1 year (in China).

641、Diagnostic Value of Combined Serum AFP, CEA, CA125 and CA199 Tests in Malignant Tumors of the Digestive System-Weizheng Zhu (2020)-Data incompleteness (in China).

642、Clinical significance of serum CA242, CA19-9 and CEA levels in patients with colorectal cancer-Yukun Zhu (2004)-Exclusion criteria met; no tumor marker thresholds specified (in China).

643、Efficacy of cetuximab in combination with oxaliplatin and capecitabine in the treatment of advanced colorectal cancer and the effect on patients' immune function-Jiafen Zhuang (2023)-The content of this literature is not relevant to the diagnosis of colorectal cancer (in China).

644、Diagnostic value of serum CA199 and CEA tests for colorectal cancer-Shanshan Zhuang (2010)-Data incompleteness (in China).

645、Clinical value and prognostic surveillance study of combined detection of multiple tumor markers in the diagnosis of colorectal cancer-Chaoshi Zou (2018)-Exclusion criteria met; study participants followed for more than 1 year (in China).

646、Analysis of the diagnostic utility of combined serum tumor marker testing in colorectal cancer-Xia Zou (2019)-Data incompleteness (in China).

647、Analysis of the correlation between serum tumor marker tests and colorectal cancer-Xiaoyun Zhou (2012)-Data incompleteness (in China).

648、APPLICATION OF COMBINED DETECTION OF CEA, AFP, CA199 AND CA50 IN SCREENING FOR GASTROINTESTINAL TUMORS IN HEALTHY INDIVIDUALS-Chen Ping (2019)-Exclusion criteria met; no tumor marker thresholds specified.

649、Serum sAPRIL: a potential tumor-associated biomarker to colorectal cancer-Weifeng Ding (2013)-Exclusion criteria met; no tumor marker thresholds specified.

650、Diagnostic accuracy of the multi-target stool DNA test in detecting colorectal cancer: A hospital-based study-Han-Lu Gao (2023)-Exclusion criteria met; no tumor marker thresholds specified.

651、Identification of CD147-positive extracellular vesicles as novel non-invasive biomarkers for the diagnosis and prognosis of colorectal cancer-Chenzheng Gu (2023)-Exclusion criteria met; no tumor marker thresholds specified.

652、Diagnostic values of MMP-7, MMP-9, MMP-11, TIMP-1, TIMP-2, CEA, and CA19-9 in patients with colorectal cancer-Xiwen Huang (2021)-Exclusion criteria met; no tumor marker thresholds specified.

653、CD4(+) Memory Stem T Cell in Peripheral Blood: A Promising Immune Index for Early Screening and Auxiliary Diagnosis of Colorectal Cancer-Yan Lu (2021)-Exclusion criteria met; no tumor marker thresholds specified.

654、Clinical Significance of Serum Haptoglobin and Protein Disulfide-Isomerase A3 in the Screening, Diagnosis, and Staging of Colorectal Cancer-Yajin Niu (2022)-Exclusion criteria met; no tumor marker thresholds specified (in China).

655、[The value of NLR, FIB, CEA and CA19-9 in colorectal cancer]-X Qian (2021)-Exclusion criteria met; no tumor marker thresholds specified.

656、Prevalence of elevated Anti-p53 in Chinese patients with upper gastrointestinal or colorectal cancer-Min Wang (2022)-Exclusion criteria met; no tumor marker thresholds specified (in China).

657、Clinical significance and diagnostic value of serum NSE, CEA, CA19-9, CA125 and CA242 levels in colorectal cancer-Hai Luo (2020)-Exclusion criteria met; no method of detection (in China).

658、Correlation study between serum neuro-specific enolase and gastric and colorectal cancers-Hai Luo (2020)-Exclusion criteria met; no method of detection (in China).

659、The diagnostic value of serum carcino-embryonic antigen, alpha fetoprotein and carbohydrate antigen 19-9 for colorectal cancer-Yan-Rong Wang (2014)-Exclusion criteria met; no method of detection.

660、Zinc-α-2-glycoprotein: a candidate biomarker for colon cancer diagnosis in Chinese population-Yingming Xue (2014)-Exclusion criteria met; no method of detection (in China).

661、Diagnostic value of combined serum CEA, CA19-9 and CA242 testing for colorectal cancer-Zhiqiang Zhong (2008)-Data incompleteness (in China)

662、Value of serum carbohydrate antigen 19-9 for predicting extrahepatic metastasis in patients with liver metastasis from colorectal carcinoma-Atsushi Sasaki (2005)-Data not available.

663、Comparative analysis of CA 242 and CA 19-9 serum tumor markers in colorectal cancer patients. A longitudinal evaluation-A Spila (2001)-Data not available.

664、MRI and correlation between TNM and CEA, CA19.9, AFP in rectal cancer Experience of a single academic surgical center-Paolo Del Rio (2021)-Data not available.

665、Matrix metalloproteinase 2 and tissue inhibitor of matrix metalloproteinases 2 in the diagnosis of colorectal adenoma and cancer patients-Magdalena Groblewska (2010)-Data not available.

666、[Clinical application value of combined detection of serum miR-378 and miR-21 in gastric cancer]-S K Huang (2018)-Data not available.

667、Evaluation of serum CEA for the gastrointestinal cancer diagnosis using different cut-off values-D.-Z. Ji (2016)-Data not available.

668、Compare the Diagnostic and Prognostic Value of MLR, NLR and PLR in CRC Patients-Yanli Kang (2021)-Data not available.

669、Prognostic Role of Carcinoembryonic Antigen and Carbohydrate Antigen 19-9 in Stage IV Colorectal Cancer-Hidetaka Kawamura (2022)-Data not available.

670、Carbohydrate antigen 19-9 plus carcinoembryonic antigen for prognosis in colorectal cancer: An observational study-Jong O Lee (2022)-Data not available.

671、Tumor markers in patients with relapse of colorectal carcinoma-Ludmila Lipská (2007)-Data not available.

672、[Assay of serum carbohydrate antigen (CA) 19-9 in the diagnosis of gastric cancer]-K

Nishida (1986)-Data not available.
673、[Clinical evaluation of CA-50 in cases with colorectal cancer]-O Nishida (1989)-Data not available.
674、[Clinical evaluation of a combination assay of CEA, CA-19-9 and TPA in patients with colorectal cancer]-O Nishida (1988)-Data not available.
675、Clinical Value of Serum CEA, CA24-2 and CA19-9 in Patients with Colorectal Cancer-Hui Rao (2021)-Data not available.
676、The effect of colonoscopy on tumor markers-E Scapa (1997)-Data not available.
677、Improved sensitivity in the diagnosis of gastro-intestinal tumors by fuzzy logic-based tumor marker profiles including the tumor M2-PK-Joachim Schneider (2005)-Data not available.
678、Comparison of serum tumor associated material (TAM) with conventional biomarkers in cancer patients-Jian Shu (2012)-Data not available.
679、CEA and CA 19-9 are still valuable markers for the prognosis of colorectal and gastric cancer patients-Abdullah Sisik (2013)-Data not available.
680、[Detection of serum p53 antibodies in colorectal cancer patients and the clinical significance of postoperative monitoring]-A Takeda (1999)-Data not available.
681、The utility and prognostic value of CA 19-9 and CEA serum markers in the long-term follow up of patients with colorectal cancer. A single-center experience over 13 years-Volkan Tumay (2020)-Data not available.
682、[Prognostic study of preoperative serum levels of CEA and CA 19-9 in colorectal cancer]-Masahiro Uehara (2007)-Data not available.
683、[CEA, CA 19.9 and CA 195 in patients with colorectal carcinoma. ROC analysis]-J Vallejo (1999)-Data not available.
684、[Expression and significance of serum microRNA-135a-5p level in colorectal cancer]-Q J Wang (2016)-Data not available.
685、The Prognostic Value of Preoperative Serum CEA and CA19-9 Values in Stage I-III Colorectal Cancer-Ren-fie Wang (2014)-Data not available.
686、Adjuvant radiochemotherapy of stage II and III rectal adenocarcinoma: role of CEA and CA 19-9-Christian Weissenberger (2005)-Data not available.
687、Clinical Application of Serum Tumor Abnormal Protein (TAP) in Colorectal Cancer Patients-Xue-Yan Wu (2015)-Data not available.
688、Is frequent measurement of tumor markers beneficial for postoperative surveillance of colorectal cancer? - Mitsuru Yokota-Mitsuru Yokota (2023)-Data not available.
689、[The optimal combination of serum tumor markers with bioinformatics in diagnosis of colorectal carcinoma]-Jie-kai Yu (2004)-Data not available.
690、Diagnostic value of the combined serum CEA, CA199, CA724 and EMA test for colorectal cancer-Chao Bi (2008)-Data not available (in China).
691、Analysis of the value of serum TSGF, CEA and CA199 in the diagnosis of colorectal cancer-Wenshui Du (2013)-Data incompleteness (in China).
692、To investigate the diagnostic value of the combined detection of serum AFP, CA72-4, CEA and CA199 on the molecular mechanism of colorectal carcinogenesis and the diagnosis of gastrointestinal malignancies-Yonghong Fan (2013)-Data incompleteness (in China).
693、Analysis of the clinical validity of combined serum tumor marker tests for the diagnosis of colorectal cancer -Gang Gao (2021)-Data not available (in China).

694、Analysis of the diagnostic value of a combination of 4 tumor markers in gastrointestinal diseases-Huiquan Gao (2017)-Data incompleteness (in China).

695、A study of the clinical significance of five preoperative serum tumor markers in the detection of rectal cancer-Shu Gao (2015)-Data not available (in China).

696、Expression of CEA and CA19-9 in colorectal cancer tissue and its clinical significance-Guo zhong Gu (2017)-Data not available (in China).

697、Value of NLR with FIB and CEA and CA19-9 in colorectal cancer-Qiang Li (2022)-Data not available (in China).

698、Diagnostic value of combined serum tumor marker testing in digestive malignancies-Ruijun Li (1997)-Data not available (in China).

699、The value of serum tumor markers in the diagnosis of colorectal cancer-Shui Li (2022)-Data not available (in China).

700、Exploring the diagnostic application of combined serum tumor marker testing in colorectal cancer-Weiwei Li (2021)-Data not available (in China).

701、The significance of combining serum CEA, CA724 and CA199 in the diagnosis of colorectal cancer-Shudong Ma (2013)-Data not available (in China).

702、Diagnostic value of combined detection of glycoantigen 199, carcinoembryonic antigen CEA and glycoantigen 242 in colorectal cancer-Shenglan Meng (2023)-Data not available (in China).

703、Significance of combined CEA, CA199 and CA242 testing in colorectal cancer-Yandong Shen (2021)-Data not available (in China).

704、Analysis of pre- and post-operative changes in serum tumor markers and their clinical significance in patients with colorectal cancer-Guangyong Shi (2008)-Data not available (in China).

705、Analysis of the value of combined faecal occult blood testing and tumor markers in colorectal cancer-Kun Su (2023)-Data not available (in China).

706、Diagnostic value of combined serum alpha-fetoprotein, glycoconjugate antigen 19-9, carcinoembryonic antigen and ferritin in colorectal cancer liver metastases-Wei Sun (2022)-Data not available (in China).

707、Early diagnostic value of three serum tumor marker panel tests for colorectal cancer-Yijun Sun (2008)-Data not available (in China).

708、The value of tumor marker testing in the diagnosis of gastrointestinal tumors-Yuling Tian (2007)-Data not available (in China).

709、Clinical value of eight tumor markers combined with chemiluminescence assay in the diagnosis of malignant tumors-Li Wang (2011)-Data incompleteness (in China).

710、Analysis of the effect of using combined serum tumor marker testing in colorectal cancer-Yahui Wang (2021)-Data not available (in China).

711、The value of CK19mRNA, CEA and CA199 for the prognostic assessment of colorectal cancer and its significance in follow-up diagnosis-Jixu Gao (2014)-The content of this literature is not relevant to the diagnosis of colorectal cancer (in China).

712、Clinical significance of CA19-9 and CEA in the preoperative diagnosis of colorectal cancer and detection of postoperative recurrence-Jianqin Hong (2011)-No data available (in China).

713、Combined CEA, CA199, CA724, AFP, CA125 test for the diagnosis of colorectal cancer and its targeted therapy research-Shaoying Wu (2022)-Data not available (in China).

714、Clinical study of combined serum CEA, CA199 and CA242 testing in patients with colorectal

cancer-Hui Xiang (2020)-Data not available (in China).
715、Analysis of the clinical impact and value of gastrointestinal tumor markers in the diagnosis of colorectal cancer-Yingzhu Xiao (2018)-Data not available (in China).
716、Tumor markers CEA and CA199 in the diagnosis and monitoring of colorectal cancer-Biao Xiong (2009)-Data not available (in China).
717、Analysis of the practical value of combined tumor marker testing for the diagnostic sensitivity of colorectal cancer-Ailei Xu (2010)-Data not available (in China).
718、The value of combined CEA, CA199 and CA125 testing for the clinical diagnosis of colorectal cancer-Bi Yao (2011)-Data not available (in China).
719、The value of serum tumor marker combination testing in the clinical diagnosis of colorectal cancer-Yun Zhang (2022)-Data not available (in China).

Data S2 :Exclusion List of Studies and Search

The literature search strategy of SinoMed Database were made as follows: (CA19-9 OR CA199 OR CA-19-9 OR gastrointestinal tumor antigen) AND (carcinoembryonic antigen OR CEA OR CD66e antigen) AND (colorectal tumor OR colorectal cancer OR colon tumor OR rectal tumor OR rectal cancer OR colon cancer OR colon cancer OR colon cancer associated tumor OR colon cancer associated tumor OR colon cancer associated tumor OR colon cancer associated tumor OR Lynch's syndrome shaped cancer OR Lynch's disease familial syndrome OR Lynch's syndrome OR Adenomatous polyposis coli OR Colus polyposis OR Familial polyposis syndrome OR Adenomatous polyposis coli OR Familial multiple adenocarcinoma of the colon OR Familial polyposis coli OR Familial polyposis coli OR Adenomatous polyposis coli OR Gardner's syndrome OR Rectal tumor or Anal tumor or Anal cancer or Anal gland tumor or Perianal gland tumor)

The literature search strategy of CNKI Database were made as follows:(SU=CA19-9 OR SU=CA199 OR SU=CA-19 9 OR SU=Gastrointestinal tumor antigen) AND (SU=Carcinoembryonic antigen OR SU=CEA OR SU=CD66e antigen) AND (SU=Colorectal cancer OR SU=Colorectal cancer OR SU=Colorectal cancer OR SU=Colorectal cancer OR SU=Colorectal cancer OR SU=Colorectal cancer or SU=Rectal Cancer OR SU=Rectal Cancer OR SU=Colorectal Cancer OR SU=Colorectal Tumor OR SU=Colon Cancer OR SU=Colon Rectal Tumor OR SU=Colon Cancer OR SU=Colonitis Associated Tumor OR SU=Cancer OR SU=Familial Lynch Syndrome disease OR SU=Lynch's syndrome OR SU=Colonic adenomatous polyposis OR SU=Colonic polyposis syndrome OR SU=Familial polyposis syndrome OR SU=Colonic adenomatous polyposis or SU=Familial multiple adenocarcinoma of the colon or SU=Familial polyposis polyposis or SU=familial intestinal polyposis or SU=adenomatous polyposis intestinalis or SU=Gardner's syndrome or SU=rectal tumor or SU=anal tumor or SU=anal cancer or SU=cancer of the anal glands or SU=cancer of the perianal glands) WANFON: Subject. (CEA OR carcinoembryonic antigen OR CA19-9 OR glycan antigen 19-9) AND (colorectal cancer OR rectal cancer)

The literature search strategy of Pubmed Database were made as follows:(CD66e Antigen OR CD66e Antigens OR Carcinoembryonic Antigen OR Carcinoembryonic Antigens OR CEA)and)CA19-9 OR CA199 OR CA-19-9 OR CA-19-9 Antigen OR CA-19-9 Antigens) and (Colon cancer or colon cancer OR colorectal cancer OR colon cancer OR colorectal cancer or colitis-related cancer or type S cancer or Lynch syndrome family syndrome or Lynch syndrome Colonic adenomatous polyposis or Colus polyposis or familial polyposis syndrome or colonic adenomatous polyposis or familial multiple polyposis of the colon or familial intestinal polyposis or intestinal adenomatous polyposis or Gardner syndrome or Rectal tumor or anal tumor or anal cancer or anal gland tumor or perianal gland tumor)

The literature search strategy of Web of science Database were made as follow:(TS=CD66e Antigen OR TS=CD66e Antigens OR TS=Carcinoembryonic Antigen OR TS=Carcinoembryonic Antigens OR TS=CEA)and(TS=CA19-9 OR TS=CA199 OR TS=CA-19-9 OR TS=CA-19-9 Antigen OR TS=CA-19-9 Antigens) and (TS=Colon cancer or TS=colon cancer OR TS=colorectal cancer OR TS=colon cancer OR TS=colorectal cancer or TS=colitis-related cancer or TS=type S cancer or TS=Lynch syndrome family syndrome or TS=Lynch syndrome Colonic adenomatous

polyposis or TS=Colus polyposis or TS=familial polyposis syndrome or TS=colonic adenomatous polyposis or TS=familial multiple polyposis of the colon or TS=familial intestinal polyposis or TS=intestinal adenomatous polyposis or TS=Gardner syndrome or TS=Rectal tumor or TS=anal tumor or TS=anal cancer or TS=anal gland tumor or TS=perianal gland tumor)

The literature search strategy of Wan Fang Database were made as follow: Subject: (("CA19-9" OR "CA199" OR "CA-19 9" OR "gastrointestinal tumor antigen")and ("carcinoembryonic antigen" OR "CEA" OR "CD66e antigen")and("colorectal tumor" OR "colorectal cancer" OR "colon cancer" OR "rectal tumor" OR "rectal cancer" OR "colorectal cancer" OR "colorectal neoplasms" OR "colorectal tumors" OR "colorectal cancer" OR "colon-associated neoplasms" OR "colitis associated neoplasms" OR "S shaped cancers" OR "familial Lynch syndrome" OR "Lynch syndrome" OR "colonic adenomatous polyposis" OR "colonic polyposis" OR "familial polyposis syndrome" OR "colonic adenomatous polyposis" OR "colonic familial polyposis adenocarcinoma" or "familial polyposis" or "familial adenomatous polyposis of the colon" or "familial adenomatous polyposis of the bowel" or "Gardner's syndrome" or "rectal tumor" or "anal tumor" or "anal carcinoma" or "tumor of the anal glands" or "tumor of the perianal glands"))))
