# Supplementary material for: Diagnostic value of combined serum CEA and CA19-9 in colorectal cancer A meta-analysis
Source: iScience. 2026 May 4;29(5):115639. doi: 10.1016/j.isci.2026.115639 (PMC13157001; doi:10.1016/j.isci.2026.115639)
Supplement: Data S3. PRISMA 2020 checklist: where to report each item in a systematic review and relevant descriptions [file mmc2.pdf]

PRISMA

# PRISMA 2020 Checklist

DATA S3:PRISMA 2020 Checklist: Where to Report Each Item in a Systematic Review and Relevant Descriptions

| Section and Topic | Item # | Checklist item | Location where item is reported |
|---|---|---|---|
| **TITLE** | | | |
| Title | 1 | Identify the report as a systematic review. | Summary (p.1) |
| **ABSTRACT** | | | |
| Abstract | 2 | See the PRISMA 2020 for Abstracts checklist. | Summary (p.1) |
| **INTRODUCTION** | | | |
| Rationale | 3 | Describe the rationale for the review in the context of existing knowledge. | Introduction, paragraphs 1–4 |
| Objectives | 4 | Provide an explicit statement of the objective(s) or question(s) the review addresses. | Introduction, final paragraph |
| **METHODS** | | | |
| Eligibility criteria | 5 | Specify the inclusion and exclusion criteria for the review and how studies were grouped for the syntheses. | STAR Methods: Inclusion and Exclusion Criteria |
| Information sources | 6 | Specify all databases, registers, websites, organisations, reference lists and other sources searched or consulted to identify studies. Specify the date when each source was last searched or consulted. | STAR Methods: Search Strategy and Data Sources — 6 databases (CNKI, Wanfang, CMCC, SinoMed, Web of Science, PubMed); searched up to January 20, 2024 |
| Search strategy | 7 | Present the full search strategies for all databases, registers and websites, including any filters and limits used. | STAR Methods: Search Strategy and Data Sources — MeSH terms and Boolean operators (AND/OR) described; full search strategy available from lead contact |
| Selection process | 8 | Specify the methods used to decide whether a study met the inclusion criteria of the review, including how many reviewers screened each record and each report retrieved, whether they worked independently, and if applicable, details of automation tools used in the process. | STAR Methods: Data Extraction — two independent reviewers screened records and extracted data; discrepancies resolved by a third reviewer |
| Data collection process | 9 | Specify the methods used to collect data from reports, including how many reviewers collected data from each report, whether they worked independently, any processes for obtaining or confirming data from study investigators, and if applicable, details of automation tools used in the process. | STAR Methods: Data Extraction — standardized extraction form used; two independent reviewers; no automation tools used |
| Data items | 10a | List and define all outcomes for which data were sought. Specify whether all results that were compatible with each outcome domain in each study were sought (e.g. for all measures, time points, analyses), and if not, the methods used to decide which results to collect. | STAR Methods: Quantification and Statistical Analysis — outcomes: sensitivity, specificity, PLR, NLR, DOR, AUC for CEA, CA19-9, and combined testing |
| | 10b | List and define all other variables for which data were sought (e.g. participant and intervention characteristics, funding sources). Describe any assumptions made about any missing or unclear information. | STAR Methods: Data Extraction — variables: author, year, study design, detection method, threshold, TP/FP/TN/FN; missing |

DATA S3:PRISMA 2020 Checklist: Where to Report Each Item in a Systematic Review and Relevant Descriptions

| Section and Topic | Item # | Checklist item | Location where item is reported |
|---|---|---|---|
| | | | data assumed absent if not reported |
| Study risk of bias assessment | 11 | Specify the methods used to assess risk of bias in the included studies, including details of the tool(s) used, how many reviewers assessed each study and whether they worked independently, and if applicable, details of automation tools used in the process. | STAR Methods: Quality Assessment — QUADAS-2 tool; two independent reviewers; four domains assessed; disagreements resolved by consensus |
| Effect measures | 12 | Specify for each outcome the effect measure(s) (e.g. risk ratio, mean difference) used in the synthesis or presentation of results. | STAR Methods: Quantification and Statistical Analysis — sensitivity, specificity, PLR, NLR, DOR, and AUC reported with 95% CI for all three testing strategies |
| Synthesis methods | 13a | Describe the processes used to decide which studies were eligible for each synthesis (e.g. tabulating the study intervention characteristics and comparing against the planned groups for each synthesis (item #5)). | STAR Methods: Quantification and Statistical Analysis — bivariate random-effects model; STATA 12; studies grouped by testing strategy (CEA alone, CA19-9 alone, combined) |
| | 13b | Describe any methods required to prepare the data for presentation or synthesis, such as handling of missing summary statistics, or data conversions. | STAR Methods: Quantification and Statistical Analysis — 2×2 contingency data extracted directly or calculated from reported Se/Sp; no imputation performed |
| | 13c | Describe any methods used to tabulate or visually display results of individual studies and syntheses. | Results, Sections 2.2–2.9; Tables 1–4; Figures 1–11 — forest plots, SROC curves, Fagan's nomogram, likelihood ratio scatter plots, bivariate boxplots |
| | 13d | Describe any methods used to synthesize results and provide a rationale for the choice(s). If meta-analysis was performed, describe the model(s), method(s) to identify the presence and extent of statistical heterogeneity, and software package(s) used. | STAR Methods: Quantification and Statistical Analysis — bivariate random-effects model (STATA 12); HSROC model for SROC curves; $I^2$ statistic for heterogeneity |
| | 13e | Describe any methods used to explore possible causes of heterogeneity among study results (e.g. subgroup analysis, meta-regression). | Discussion, Section 3 — heterogeneity sources discussed narratively (study design, detection platforms, control definitions); formal subgroup analysis or meta-regression not performed; acknowledged as a limitation |
| | 13f | Describe any sensitivity analyses conducted to assess robustness of the synthesized results. | Results, Section 2.9; Figures |

DATA S3:PRISMA 2020 Checklist: Where to Report Each Item in a Systematic Review and Relevant Descriptions

| Section and Topic | Item # | Checklist item | Location where item is reported |
|---|---|---|---|
| | | | 10–11 — leave-one-out sensitivity analysis conducted for all three testing strategies |
| Reporting bias assessment | 14 | Describe any methods used to assess risk of bias due to missing results in a synthesis (arising from reporting biases). | STAR Methods: Quantification and Statistical Analysis; Results, Section 2.8; Figure 9 — Deeks' funnel plot asymmetry test; slope test P-value reported |
| Certainty assessment | 15 | Describe any methods used to assess certainty (or confidence) in the body of evidence for an outcome. | Not formally assessed — GRADE or equivalent certainty-of-evidence assessment was not conducted; acknowledged as a study limitation |
| **RESULTS** | | | |
| Study selection | 16a | Describe the results of the search and selection process, from the number of records identified in the search to the number of studies included in the review, ideally using a flow diagram. | Results, Section 2.1; Figure 1 — PRISMA flow diagram: 7,024 records identified; 95 studies included |
| | 16b | Cite studies that might appear to meet the inclusion criteria, but which were excluded, and explain why they were excluded. | Not separately reported — exclusion reasons not listed study-by-study; overall exclusion rationale summarized in STAR Methods: Inclusion and Exclusion Criteria and Figure 1 (PRISMA flow diagram) |
| Study characteristics | 17 | Cite each included study and present its characteristics. | Results, Section 2.2; Tables 1–2 — 95 studies, 22,821 subjects; study design, publication year, detection method, threshold, and 2×2 data presented |
| Risk of bias in studies | 18 | Present assessments of risk of bias for each included study. | Results, Section 2.3; Table 3 — QUADAS-2 risk of bias assessment across four domains for all 95 included studies |
| Results of individual studies | 19 | For all outcomes, present, for each study: (a) summary statistics for each group (where appropriate) and (b) an effect estimate and its precision (e.g. confidence/credible interval), ideally using structured tables or plots. | Results, Section 2.4; Table 4; Figures 3–4 — pooled Se, Sp, PLR, NLR, DOR with 95% CI presented per testing strategy; individual study forest plots provided |
| Results of | 20a | For each synthesis, briefly summarise the characteristics and risk of bias among contributing studies. | Results, Section 2.4 — sample sizes (cases/controls) and $I^2$ values |

DATA S3:PRISMA 2020 Checklist: Where to Report Each Item in a Systematic Review and Relevant Descriptions

| Section and Topic | Item # | Checklist item | Location where item is reported |
|---|---|---|---|
| syntheses | | | summarized for each of the three testing strategies |
| | 20b | Present results of all statistical syntheses conducted. If meta-analysis was done, present for each the summary estimate and its precision (e.g. confidence/credible interval) and measures of statistical heterogeneity. If comparing groups, describe the direction of the effect. | Results, Sections 2.4–2.6; Table 4; Figure 5 — pooled Se, Sp, DOR, AUC with 95% CI; SROC curves; $I^2$ values (91.2%, 91.4%, 91.2%) reported |
| | 20c | Present results of all investigations of possible causes of heterogeneity among study results. | Discussion, Section 3 — heterogeneity sources discussed narratively; formal statistical investigation (meta-regression/subgroup analysis) not conducted |
| | 20d | Present results of all sensitivity analyses conducted to assess the robustness of the synthesized results. | Results, Section 2.9; Figures 10–11 — leave-one-out sensitivity analysis for all three strategies; variation ranges of pooled estimates reported |
| Reporting biases | 21 | Present assessments of risk of bias due to missing results (arising from reporting biases) for each synthesis assessed. | Results, Section 2.8; Figure 9 — Deeks' funnel plot with slope test P-value for all three testing strategies |
| Certainty of evidence | 22 | Present assessments of certainty (or confidence) in the body of evidence for each outcome assessed. | Not applicable — formal certainty-of-evidence (e.g., GRADE) assessment was not conducted; acknowledged as a study limitation |
| **DISCUSSION** | | | |
| Discussion | 23a | Provide a general interpretation of the results in the context of other evidence. | Discussion, Section 3, paragraphs 1–5 — results interpreted in context of prior meta-analyses and clinical diagnostic practice |
| | 23b | Discuss any limitations of the evidence included in the review. | Limitations of the Study — five limitations discussed: high $I^2$, limited combined-test study number (n=36), Asian-predominant population, spectrum bias from variable control definitions, possible residual publication bias |
| | 23c | Discuss any limitations of the review processes used. | Limitations of the Study — limitations of review process: no |

DATA S3:PRISMA 2020 Checklist: Where to Report Each Item in a Systematic Review and Relevant Descriptions

| Section and Topic | Item # | Checklist item | Location where item is reported |
|---|---|---|---|
| | | | subgroup/meta-regression analysis; no GRADE assessment; Chinese-language literature predominance may introduce retrieval bias |
| | 23d | Discuss implications of the results for practice, policy, and future research. | Discussion, final paragraph; Limitations of the Study — combined CEA+CA19-9 recommended as preferred clinical strategy; standardized prospective multicenter studies called for |
| **OTHER INFORMATION** | | | |
| Registration and protocol | 24a | Provide registration information for the review, including register name and registration number, or state that the review was not registered. | STAR Methods: Additional Resources — PROSPERO registration number: CRD420251101973 |
| | 24b | Indicate where the review protocol can be accessed, or state that a protocol was not prepared. | No separate protocol document prepared or published; PROSPERO registration serves as the pre-registered protocol |
| | 24c | Describe and explain any amendments to information provided at registration or in the protocol. | No amendments were made to the registered protocol |
| Support | 25 | Describe sources of financial or non-financial support for the review, and the role of the funders or sponsors in the review. | Acknowledgments — funded by Natural Science Foundation of Guizhou Province (QiankehehJichuZK[2024]yiban295) and Program for High-Level Innovative Thousand-Level Talents in Guizhou Province; funders had no role in study design or reporting |
| Competing interests | 26 | Declare any competing interests of review authors. | Declaration of Interests — all authors declare no competing interests; Declaration of Interest form submitted per iScience/Cell Press requirements |
| Availability of data, code and other materials | 27 | Report which of the following are publicly available and where they can be found: template data collection forms; data extracted from included studies; data used for all analyses; analytic code; any other materials used in the review. | STAR Methods: Data and Code Availability — 2×2 contingency data provided in Tables 1–2; STATA 12 used for all analyses; no custom code generated; additional data |

PRISMA 2020 Checklist

DATA S3:PRISMA 2020 Checklist: Where to Report Each Item in a Systematic Review and Relevant Descriptions

| Section and Topic | Item # | Checklist item | Location where item is reported |
|---|---|---|---|
| | | | available from lead contact (372422022@qq.com) |

*From:* Page MJ, McKenzie JE, Bossuyt PM, Boutron I, Hoffmann TC, Mulrow CD, et al. The PRISMA 2020 statement: an updated guideline for reporting systematic reviews. BMJ 2021;372:n71. doi: 10.1136/bmj.n71.
This work is licensed under CC BY 4.0. To view a copy of this license, visit https://creativecommons.org/licenses/by/4.0/
